# Machine Learning Predicts the Presence of 2,4,6-Trinitrotoluene in Sediments of a Baltic Sea Munitions Dumpsite Using Microbial Community Compositions

**DOI:** 10.3389/fmicb.2021.626048

**Published:** 2021-09-29

**Authors:** René Janßen, Aaron J. Beck, Johannes Werner, Olaf Dellwig, Johannes Alneberg, Bernd Kreikemeyer, Edmund Maser, Claus Böttcher, Eric P. Achterberg, Anders F. Andersson, Matthias Labrenz

**Affiliations:** ^1^Biological Oceanography, Leibniz Institute for Baltic Sea Research Warnemünde, Rostock, Germany; ^2^Marine Biogeochemistry, GEOMAR Helmholtz Centre for Ocean Research Kiel, Kiel, Germany; ^3^Marine Geology, Leibniz Institute for Baltic Sea Research Warnemünde, Rostock, Germany; ^4^Science for Life Laboratory, Department of Gene Technology, School of Engineering Sciences in Chemistry, Biotechnology and Health, KTH Royal Institute of Technology, Solna, Sweden; ^5^Institute of Medical Microbiology, Virology and Hygiene, University of Rostock, Rostock, Germany; ^6^Institute of Toxicology and Pharmacology for Natural Scientists, University Medical School Schleswig−Holstein, Kiel, Germany; ^7^State Ministry of Energy, Agriculture, The Environment, Nature and Digitization, Kiel, Germany

**Keywords:** munition compounds, Kolberger Heide, mercury, random forest, 16S rRNA gene amplicon sequencing, monitoring, fingerprint, TNT

## Abstract

Bacteria are ubiquitous and live in complex microbial communities. Due to differences in physiological properties and niche preferences among community members, microbial communities respond in specific ways to environmental drivers, potentially resulting in distinct microbial fingerprints for a given environmental state. As proof of the principle, our goal was to assess the opportunities and limitations of machine learning to detect microbial fingerprints indicating the presence of the munition compound 2,4,6-trinitrotoluene (TNT) in southwestern Baltic Sea sediments. Over 40 environmental variables including grain size distribution, elemental composition, and concentration of munition compounds (mostly at pmol⋅g^–1^ levels) from 150 sediments collected at the near-to-shore munition dumpsite Kolberger Heide by the German city of Kiel were combined with 16S rRNA gene amplicon sequencing libraries. Prediction was achieved using Random Forests (RFs); the robustness of predictions was validated using Artificial Neural Networks (ANN). To facilitate machine learning with microbiome data we developed the R package phyloseq2ML. Using the most classification-relevant 25 bacterial genera exclusively, potentially representing a TNT-indicative fingerprint, TNT was predicted correctly with up to 81.5% balanced accuracy. False positive classifications indicated that this approach also has the potential to identify samples where the original TNT contamination was no longer detectable. The fact that TNT presence was not among the main drivers of the microbial community composition demonstrates the sensitivity of the approach. Moreover, environmental variables resulted in poorer prediction rates than using microbial fingerprints. Our results suggest that microbial communities can predict even minor influencing factors in complex environments, demonstrating the potential of this approach for the discovery of contamination events over an integrated period of time. Proven for a distinct environment future studies should assess the ability of this approach for environmental monitoring in general.

## Introduction

Microbes are the most diverse, abundant, and ubiquitous life forms on Earth. They live in complex microbial communities, which can react rapidly to environmental changes, a result of consistent evolutionary pressures applied by fluctuating conditions (Lindh and Pinhassi, 2018). The developed variety of physiologies enables communities to respond in specific ways to environmental drivers, hence functioning as indicators for surrounding conditions. This principle was demonstrated for very different habitats: it was possible to match individual human skin microbiomes with those on the occupant’s household surfaces (Wilkins et al., 2017), to associate subway microbiomes to the major cities they were located in Ryan (2019) or to distinguish microbial communities in the brackish Baltic Sea along the salinity gradient (Herlemann et al., 2011) and its anoxic regions (Thureborn et al., 2016). However, relevant indicative fractions of the communities, conceivably acting as microbial fingerprints, may only emerge by analyzing a sufficiently large number of communities. Next generation sequencing allows for processing such larger amounts of samples to extract this information, but it might be accompanied by a large portion of irrelevant data with regard to the particular indication.

The ensemble classifier Random Forest (RF) is capable of identifying such potential fingerprints—even if they include non-linear relations—in large and complex data sets (Breiman, 2001). RF is among the most popular machine learning methods and has frequently been used in biological sciences (Fernández-Delgado et al., 2014). The features relevant for the model’s decisions can be assumed equivalent to an indicative fingerprint and the RF variable importance measure readily identifies them (e.g., Altmann et al., 2010; Janitza et al., 2018). Fingerprints related to community-shaping drivers are revealed by performing unsupervised classification, whereas specific influences can be targeted by the application of supervised machine learning. In microbiological studies, RF has been deployed to predict various geochemical features as well as to detect oil spills (Smith et al., 2015) and to localize the geographic origin of port water across three continents based on dominant bacterial phyla (Ghannam et al., 2020). Moitinho-Silva et al. (2017) used RF among other classifiers to separate between sponges of high and low microbial abundance. Thompson et al. (2019) used RF and artificial neural networks (ANN) to identify important taxa for the prediction of dissolved organic carbon concentrations. In a previous study we demonstrated the identification of glyphosate-impacted free-living community compositions by ANN and RF after a 82.45 nmol mL^–1^ glyphosate pulse in a lab microcosm experiment (Janßen et al., 2019b).

In this study, we are particularly interested in to what extent environmental microbial communities can reliably predict anthropogenic pollutants using the above algorithms. As a proof of principle, we tested this approach for a munitions dumpsite in the southwestern Baltic Sea, where sediments are contaminated with explosive compounds such as 2,4,6-trinitrotoluene (TNT). The munitions dumpsite Kolberger Heide in the Kiel Bight (Germany) is an approximately 1,260 ha large area of 10–15 m water depth. Conventional munition, mostly incomplete or unfused was disposed of at this site after World War II (Kampmeier et al., 2020). About 30,000 tons are estimated to be still on site, containing mainly TNT and 1,3,5-trinitroperhydro-1,3,5-triazine (RDX) as munition compounds (Böttcher et al., 2011). The containments such as mines, shells and torpedo heads display various states of corrosion (Kampmeier et al., 2020), resulting in the leakage of munition compounds (Beck et al., 2019). In addition, bare munition chunks are scattered across the sediment bed, potentially due to low-order, or incomplete detonation during blow-in-place clearance activities (Pfeiffer, 2009; Maser and Strehse, 2020). Dissolved TNT can be rapidly dissipated or metabolized in direct proximity to its source, complicating the quantification of TNT released into the environment (Elovitz and Weber, 1999; Beck et al., 2019). However, the presence of munition compounds including TNT and its transformation products in the Kolberger Heide water column samples (ca. 1–15 ng⋅L^–1^) and biota (1–24,000 ng⋅g^–1^) has been reported (Gledhill et al., 2019). Little is known about the munition compounds’ concentrations in accordant sediments.

Sediment in the Kolberger Heide is contaminated by TNT at pmol⋅g^–1^ levels. It was our aim (a) to investigate if machine learning is capable of predicting TNT in these sediments and identifying indicative microbial fingerprints; (b) to assess how robust the predictions are and which factors influenced the model’s performance; and (c) to evaluate whether a microbial fingerprint is sufficiently persistent to detect a history of TNT, indicated by TNT transformation products. Finally, we discuss how the described approach could supplement and be integrated into regular monitoring activities.

## Materials and Methods

### Collection of Sediments and Determination of Munition Compounds

One hundred sixty-seven sediment samples were collected within the Kolberger Heide munitions dumpsite and its surroundings during the course of the Umweltmonitoring für die DElaboration von Munition im Meer (UDEMM) (Environmental monitoring for the delaboration of munitions on the seabed, Greinert, 2019) project. Samples were obtained during several cruises and individual sampling events. Additional sampling took place at defined distances from mines and at a site of a controlled detonation. Sediment samples within the dumpsite were collected manually by scientific divers or using an remotely operated underwater vehicle (ROV). Outside the dumpsite’s restriction zone, surface sediments were collected using a Van Veen grab. Duplicate sediment cores were collected using a multi-corer at two sites east and west of the dumpsite (map provided in Supplementary Figure 1). Sampling was conducted in December 2016 and from June to December 2017. Supplementary Figure 2 details contextual data such as position of sample collection, cruises, and experiments as well as measured parameters. “Experiments” refer to the goal of a sampling, e.g., investigating a spatial munition compounds gradient in cardinal directions around a mine, analyzing the munition compounds distribution across a mine mound or along a sediment profile. Sediments were stored in sealable plastic bags (Whirl-paks; Nasco, Madison, WI, United States) at −20°C for subsequent munition compounds analysis using an ultra-high performance liquid chromatographic system coupled to a heated electrospray ionization source and a high resolution quadrupole/orbitrap mass analyzer (UHPLC-HESI-MS, Q Exactive, Thermo Fisher Scientific) detection after thawing and extraction using liquid chromatography-mass spectrometry (LCMS)-grade acetonitrile (Fisher). Munition compounds were measured according to Gledhill et al. (2019) including TNT, RDX, 2-amino-4,6-dinitrotoluene (2-ADNT), 4-amino-2,6-dinitrotoluene (4-ADNT), 2,4-dinitrotoluene (2,4-DNT), 2,6-dinitrotoluene (2,6-DNT), 1,3-dinitrobenzene (DNB), 1,3,5-trinitrobenzene (TNB), octahydro-1,3,5,7-tetranitro-1,3,5,7-tetrazocine (HMX), and tetryl (*N*-methyl-*N*-2,4,6-tetranitrolaniline). The TNT transformation products, 2,4-diamino-6-nitrotoluene (2,4-DANT), and 2,6-diamino-4-nitrotoluene (2,6-DANT) are not included in the Gledhill et al. (2019) suite of compounds, but were analyzed using the same method, and quantified after standardization using single-compound standards (AccuStandard, New Haven, CT, United States). For geological and molecular biology analyses sediments were slowly thawed, homogenized under a clean bench, and split into two 15 mL aliquots. The aliquots were stored at −80°C.

### Geochemical and Sedimentological Analyses

#### Sample Preparation

The frozen (−20°C) sediment samples were freeze-dried (Christ LOC-1M Alpha 1-4 and Christ Delta 1-24 LSCplus, Osterode am Harz, Germany) for 60–72 h. Except for the grain size analyses, the dried samples were homogenized in an agate ball mill (Fritsch Pulverisette, Idar-Oberstein, Germany) at 200 rpm for 10 min.

#### Carbon, Nitrogen, and Sulfur

About 10–17 mg of the sediments were weighted into tin crucibles, a spatula tip of vanadium(V) oxide (Alpha Resources, Stevensville, MI, United States) was added as catalyzer and total C, total N, and total S were determined by an elemental analyzer (EuroEA, HEKAtech, Wegberg, Germany). For total inorganic carbon, 50–70 mg of sediment was treated with 40% orthophosphoric acid and analyzed with an elemental analyzer (multiEA 4000, Analytik Jena, Jena, Germany). Total organic carbon was calculated by subtracting total inorganic carbon from total carbon. Precision and trueness were checked with in-house standards [Mecklenburg Bay Sediment Standard (MBSS), Oder Bay Sediment Standard (OBSS)] and were <3.5% (Häusler et al., 2018).

#### Mercury

The sedimentary mercury content was determined by a direct mercury analyzer (DMA 80, Milestone Srl, Italy) using 100–120 mg per analysis (50 mg for sample “Udemm1277,” which exceeded the calibration range). Precision and trueness were checked with the certified reference material BCR-142R (Community Bureau of Reference) and an in-house standard comprising Baltic Sea sediments (MBSS) and were <3 and <10%, respectively (Häusler et al., 2018). Sediments exceeding 1,000 μg Hg⋅kg^–1^ were measured three times and averaged.

#### Reactive Iron and Trace Element Contents

For determination of reactive element contents, about 200 mg of sediment material was weighed into pre-cleaned 11.5 mL polystyrene tubes and 10 mL of 0.5 M HCl was added. The tubes were shaken for 60 min at 175 rpm, followed by 6 min of centrifugation at 4,000 × *g* and filtration of the solutions with 0.45 μm syringe filters. Three procedural blanks were analyzed together with the samples. The contents of Fe, P, and trace metals in the 0.5 M HCl extracts were determined by Q-ICP-MS (iCAP Q; Thermo Fisher Scientific, Germany) after automated 50-fold dilution with 2 vol% HNO_3_ *via* a prepFAST module (Elemental Scientific, Omaha, NE, United States) and external calibration. Helium was used as collision gas (KED mode) to minimize polyatomic interferences and a Rh and Ir containing solution added online by the prepFAST module served as internal standard to compensate for matrix effects and instrument fluctuations. The calibration was checked with the international reference material SGR-1b (USGS), which underwent total acid digestion in closed PTFE vessels using a HNO_3_–HF–HClO_4_ mixture (Dellwig et al., 2019). For stable ^206/207^Pb isotope ratios the NIST SRM-981 was used as reference material (Dellwig et al., 2018). Precision and trueness of the measurements of the reference materials were <4.4 and 8.1%, respectively.

#### Grain Size Distribution

The grain size of the <2 mm sediment faction was measured using a Hydro EV accessory to the Mastersizer 3000 (Malvern Panalytical GmbH, Herrenberg, Germany). The samples were stirred at 2,500 rpm and sonicated for 10 s. Eight measurements were performed per sample, followed by purging steps with distilled water. Outliers (values exceeding 1.5 times the interquartile range) were removed and the remaining values per sediment were averaged.

### Molecular Biology and Bioinformatics

The methods described in the following were applied to the molecular biology aliquots of each sediment sample.

#### Extraction of Nucleic Acids

The sediments were collected using the appropriate collection and storage procedures for the determination of munition compounds. To retrieve the best possible results in subsequent molecular biological analyses and due to the long term presence of TNT in the Kolberger Heide, the more robust 16S rRNA gene was preferred over the more sensitive 16S rRNA as sequencing target. DNA was extracted from 250 mg wet sediment using the Qiagen PowerSoil DNA Kits or from 2,000 mg wet sediment using the Mobio PowerSoil RNA kit with the DNA elution kit (Hilden, Germany). For each kit an extraction control without sediment was processed along with regular samples.

#### Sequencing 16S rRNA Gene Amplicons

The V4 region of the 16S rRNA gene was targeted with the universal prokaryotic primer set 515f-806r (forward 5′ GTGCCAGCMGCCGCGGTAA 3′, reverse 5′ GGACTACHV GGGTWTCTAAT 3′, Caporaso et al., 2011). Indexed amplicon libraries were pooled to a concentration of 4 μM. As usual for low diversity libraries, the PhiX control was spiked into the library pools at a concentration of 40 pM (10%). Each final library pool (4 pM) was subjected to 1 of 3 consecutive individual paired-end sequencing runs using 500 cycle V2 chemistry kits on an Illumina MiSeq (Berlin, Germany). Additional information with regard to the 16S rRNA gene libraries is provided in Supplementary Figure 3.

#### Processing 16S rRNA Gene Amplicon Sequences

Amplicon read processing—including the removal of primer and two-parent chimera sequences, the quality filtering step and the taxonomic annotation—was conducted using the DADA2 pipeline v. 1.10.0 (Callahan et al., 2016) with R v. 3.5.1 (R Core Team, 2017). DADA2 corrected for sequencing errors during the generation of amplicon sequence variants (ASVs). As recommended, such a correction was applied separately for each sequencing run. The individual tables were merged afterward. Only ASVs of length from 231 to 272 bp were kept according to the expected amplicon lengths reported in Ziesemer et al. (2015).

Taxonomic annotation of herein presented data was accomplished using the Silva release 132 (Yilmaz et al., 2014), including the taxonomic changes that were proposed by Parks et al. (2018). The ASV and taxonomy table were imported to and analyzed with phyloseq v. 1.30.0 (McMurdie and Holmes, 2013) accelerated by speedyseq v. 0.1.1 (McLaren, 2020). Plots were generated using ggplot2 v. 3.3.1 (Wickham, 2016).

Amplicon sequence variants which were present in negative PCR or extraction controls and also found abundantly in actual samples were individually checked due to potential cross contamination directed from samples toward the controls. ASVs with more than 35 reads in controls were removed from the dataset. ASV00001 was excluded from this rule because it was much more abundant in actual samples (extraction control: 75 reads, samples: >10,000 reads). ASVs which were present in controls and less abundant in samples were removed. Subsequently, it was checked if any of the as important detected taxa were also present in control samples. ASV00063 belonged to the important genus *Maribacter* (4 reads in positive PCR control) and ASV00074 to *Cobetia* (5 reads in negative PCR control). As no reads were found in the extraction control and they were as abundant as up to 3,000 reads in sediments, these ASVs were left unaltered.

### Machine Learning Analyses

Machine learning analyses were conducted to evaluate whether microbial community compositions contain sufficient information about their environment and are sensitive enough to contamination to act as a proxy for in this case sea-dumped munitions. Such approach would allow to detect a variety of substances per community data set, once trained models for those substances are available. It allows for analyzing and understanding the mutual effects of TNT and bacteria on each other and thereby investigating the larger ecological context, leading to a potential application in environmental monitoring. If the goal is solely to measure TNT, traditional instrumental analyses should be conducted.

Analyses were carried out on six virtual machines provided by the German Network for Bioinformatics Infrastructure (de.NBI Cloud). The virtual machines ran Ubuntu 18.04.4 LTS as operating system on 28 Intel Xeon Gold 6140s cores with 256–512 GB memory available. RF analyses were performed utilizing R package ranger v. 0.12.1 (Wright and Ziegler, 2017). ANNs were generated with the R Keras framework v. 2.3.0.0 (Allaire and Chollet, 2020) and the TensorFlow back end v. 2.2.0 (Allaire and Tang, 2020). Our efforts to extract abundance, taxonomical and contextual data from phyloseq objects and subject those to machine learning led to the development of the R package phyloseq2ML v. 0.5.1.^1^ It facilitates modification and combining such data sets as needed—using objects of class “phyloseq” as source—and formats the data for the above mentioned machine learning implementations in R.

#### Challenges of a Small Biological Data Set

The presented data set consists of contextual subsets (e.g., by specific transects or sampled by a given method) which are likely to contain samples more similar to each other than to those of other subsets. To ensure that the model’s decision making was based on the presence of TNT rather than to a particular cruise or experiment, we developed guidelines to assess which samples were appropriate for machine-learning (ML) analyses. First, the technical replicates were averaged. Then, if for a given subset of samples all of the following questions could be answered with yes, samples had to be removed from the subset to prevent potential spurious relationships between the presence of TNT and the prediction accuracy:

For all samples from the same cruise (including biological replicates) → do they originate from the same experiment? → and the same area? → and do the sediment sampling positions have horizontal distance of <20 m → and do they only contain one class (TNT present or TNT absent) emphasized “or” (OR) is there a strong imbalance (e.g., 20 × TNT absent, 1 × TNT present)?

Following this guideline led to a removal of 17 of the original 167 sediments (Supplementary Figure 2). No samples were removed based on other criteria such as low read counts.

As about half of the sediment samples did not contain TNT and the TNT concentration within the other half of the samples was unevenly distributed (see section “TNT Contamination of Kolberger Heide Sediments”) and also given the rather small sample size, it was considered unreasonable to perform regression analyses to predict the concentration of TNT. It was decided to investigate whether TNT has an effect at all on the microbial community composition, thus the samples were categorized as “TNT present” and “TNT absent.”

#### Machine Learning Workflow

The remaining 150 samples were split into a training-validation set (in short: training set) consisting of 112 samples (75%) and a holdout test set of 38 samples (25%). This procedure was repeated to yield six different, pseudo-random splits of training and test sets. Using a random seed, the splits were reproducible.

In supervised learning, training and validation data for a model contain the independent variables and the corresponding continuous or discrete response variable. The measured TNT concentrations were categorized as response classes “absent” for concentrations below the detection limit (0.01 ng⋅g^–1^ or 0.044 pmol⋅g^–1^ wet sediment) and “present.”

Settings automatically derived from the learning process are called parameters, such as the weights between ANN nodes. Hyperparameters, instead, are model settings chosen before training has started. RFs are controlled *via* two main hyperparameters: the number of trees per forest and the number of variables “mtry” to consider for sample separation at each tree node. The default value for mtry for classification tasks is the square root of the total number of independent variables. As this default value might not be optimal for sparse data such as ASV abundance tables, a factor multiplying this number of variables was used instead and will be referred to as “mtry factor” (Hastie et al., 2009).

Random Forest models were trained on various combinations of hyperparameter values and input data to find the best combination. This process is called a grid search and combinations were compared using the out-of-bag validation error, i.e., only using the training sets and not the holdout sets. A confusion matrix was generated to calculate performance metrics. Balanced accuracy was used as score. It corrects for imbalanced response variables and allowed comparisons across training set splits, which displayed class ratios of 43–48% “TNT present” (Brodersen et al., 2010). The validation results of the six data splits were averaged to select the best performing hyperparameter values and input sets. When predicting the holdout set, the model was trained on the full training-validation set. The holdout predictions for the various input data sets took place after all hyperparameter values were determined. This is required to prevent data leakage.

#### TNT Presence Prediction Based on Random Forest Grid Search

Data sets designed as model input were threefold: (a) community data: describing data deriving from 16S rRNA gene amplicon sequencing; (b) sediment data: sediment parameters derived from geochemical and sedimentological analyses; and (c) combined, a combination of both aforementioned input sets.

The grid search with community data was performed as follows: All combinations of relative abundance thresholds, the number of trees and the mtry factor were investigated. ASVs had to be more abundant than a given threshold in at least one sample. If so, the ASVs remains without change, otherwise it was filtered out. Thresholds were: 0.02, 0.04, 0.06, 0.08, 0.1, 0.2, 0.4, 0.6, 0.8, and 1%. Each of the resulting input sets was provided to models consisting of 100, 500, 1,000, 5,000, 10,000, and 20,000 trees along with mtry factors ranging from 1 to 13 by 2. For each combination 50 models were trained and validated.

Subsequently, the filtered relative ASV abundances were accumulated by taxonomic ranks genus through phylum to train 200 models with the previously identified hyperparameter values of 10,000 trees, an mtry factor of 5, and a threshold of 0.08%.

The sediment data contained 41 independent variables including reactive element contents, sum parameters such as total nitrogen, and the grain size distribution. Hundred models were trained with 1,000, 5,000, 10,000 trees and mtry factors 1, 3, and 5. For combined input data it was found sufficient to apply the same hyperparameters as were applied to the community data.

Validation and holdout scores were tested separately for significant differences between input data sets. Equal means were tested with unequal variance and one-way analysis of variance. The results of the analysis of variance were further subjected to the Tukey’s multiple comparisons of means with 95% family-wise confidence level to identify the pairwise significances.

#### Selection of Most Important Variables

The most important variables for classification were retrieved from models trained with community, sediment and combined data. Importance for community data (0.08% threshold, genus rank) and combined data was calculated utilizing the corrected Gini impurity (Nembrini et al., 2018), followed by *p*-value estimation after Janitza et al. (2018). A 100 models using 10,000 trees and an mtry factor of 5 were trained and the results averaged. Variable importance and associated *p*-value for sediment data required the permutation-based approach by Altmann et al. (2010). A 1,000 permutations with mtry factor 1 and 10,000 trees were applied. The analysis involved elements Zr, which likely was not soluble by HCl extraction as well as Ca and Sn, where the measurement by ICP-MS was later identified as unreliable. The elements were still included in the training data, but were not reported as important and removed for other analysis such as the Spearman rank correlation.

The variables were ordered by average importance over all splits. The number of variables for further analyses were selected based on decreasing decline in importance, meaning if the variables became more similarly important to each other, the cutoff was set. Thus, 25 genera were selected with Janitza importance > 0.25, *p* < 0.01 and 9 sediment parameters with Altmann importance > 0.001 and *p* < 0.05. The most important 50 combined variables (equal to Janitza importance > 0.15 and *p* < 0.01) were compared to the 25 community and 9 sediment variables.

#### Random Forest’s Proximity Matrix for PCA Ordination and Correlation

Ordination methods are useful to explore multivariate data sets such as microbial community compositions by displaying their similarities. The proximity matrix generated by RFs is a measure of (dis-) similarity, as well. The proximity between two samples is calculated by measuring the number of times they end up in the same terminal node of the same tree of the RF, divided by the number of trees in the forest.

It can be used with unsupervised classification: a synthetic data set is added to the original data set. This consists of shuffled columns of the actual data, thus breaking all relationships between variables. The model (10,000 trees, mtry factor 1) tries to distinguish between permuted and original data and thereby identifies correlations and clusters in the actual data set. For supervised classification, the actual classes were used and no synthetic data set was required.

Principal component analysis (PCA) was performed based on the proximity matrix for the most important 25 genera. To identify microbial community shaping influences for the unsupervised classification, the sediment parameters were correlated with the PCA ordination. The function *envfit()* from R package vegan v. 2.5-6 (Oksanen et al., 2019) with 9,999 permutations was used to achieve this. Correlating parameters with *p* < 0.001 and *R*^2^ > 0.3 were displayed. The PCA ordination was performed for sediment data as described above, except the *envfit()* step. Complementary, Spearman’s rank-order correlations between sediment variables were investigated. The results were hierarchically clustered and variables with *p* < 0.01 were marked significant.

#### Assessing Robustness of Classification With Random Forest and Artificial Neural Nets

The classification consistency was examined to increase the understanding of the predictions. All 150 samples were used as training and validation set for 1,000 models (10,000 trees, mtry factor 1). Mean prediction errors <0.5 or >99.5% accuracy were rounded to 0 and 100%, respectively.

Artificial neural networks were additionally deployed to measure classification robustness across algorithms. The input data for ANNs required additional steps including the one-hot encoding of categorical variables and scaling of the independent variables: the mean of each variable was subtracted, and it was divided by the standard deviation. This yielded values centered around 0 with a standard deviation of 1. ANN grid searches were performed complementary to what is described for RF above. Results suggested that 50 nodes in the first hidden layer and 40 nodes in the second hidden layer were appropriate values, along a mini-batch training size of 4. No regularization was applied. The optimizer function Adaptive Moment Estimation outperformed Root Mean Square Propagation. Binary cross entropy was set as loss function, with accuracy as metric. Learning took a maximum of 100 epochs, stopped by an early callback if the validation loss did not decrease for two ongoing epochs. The node within the hidden layers were rectified linear unit-activated whereas the output nodes’ activation function was sigmoid. Further hyperparameters and settings were default values of the keras R package.

Performance assessment was achieved by splitting the training data into three different, non-overlapping equally proportioned subsets. Two partitions were used for training and the remaining one for validation. These three subsets were composed differently for each of the conducted 333 runs. This 333 times repeated threefold cross validation yielded a total of 999 predictions.

### Data Availability

Code, scripts and files are available under GitHub.^2^ The R package phyloseq2ML is available at https://github.com/RJ333/phyloseq2ML. Sequences were deposited in the NCBI database under BioProject ID PRJNA632711 and SRA accessions SAMN14917999–SAMN14918370. The count tables, taxonomy tables and sample data as well as the thereby generated phyloseq objects and the machine learning classification result tables were lodged at https://zenodo.org/record/4062263. Geochemical data is included in Supplementary Figure 2.

## Results

### TNT Contamination of Kolberger Heide Sediments

Of those 150 samples selected for ML, 137 contained munition compounds: 2-ADNT (127), 4-ADNT (133), 2,4-DANT (67), 2,6-DANT (52). None of the other munition compounds (2,4-DNT, 2,6-DNT, DNB, TNB, HMX, RDX, Tetryl) were detected in more than eight sediments (Supplementary Figure 2).

2,4,6-Trinitrotoluene concentration showed a median of 0 and a mean of 16.29 pmol⋅g^–1^ wet sediment among the 150 samples. It was detected in 68 samples or 45.3% of the samples; TNT was determined at <25 pmol⋅g^–1^ in 65 samples. Therefore, a binary classification approach was adopted. Notably, the highest value of 1,587 pmol⋅g^–1^ was found in sediments retrieved from a detonation site, where exposed munition chunks were spread over the sea floor.

The heavy metals mercury and lead were used as proxies for primary explosive compounds in conventional ammunition, which potentially could be present at the dumpsite; chemical warfare agents can contain arsenic. Mercury contents ranged in Kolberger Heide sediments from 3.7 to 4,503 μg Hg⋅kg^–1^ dry sediment, with a median of 21 μg Hg⋅kg^–1^ and 15 samples exceeding 450 μg Hg⋅kg^–1^. The maximal content of 4,503 μg Hg⋅kg^–1^ was found during a line transect, where samples were taken every 20 m. The neighboring samples to the maximal value contained 8 and 12 μg Hg⋅kg^–1^, demarcating a precise area of elevated Hg presence. Arsenic appeared on level between 0.4 and 4.8 mg⋅kg^–1^ with a median of 0.8 mg⋅kg^–1^ and lead ranged from 1 to 75 mg⋅kg^–1^ with a median of 2 mg⋅kg^–1^.

### Community Data Predicts TNT Presence More Accurately Than Sediment Data

The microbial community composition of the sediments was investigated for measurable effects caused by the TNT. A total of 259 16S rRNA gene libraries were selected to be appropriate for ML purposes. The selected libraries had a mean size of 82,219 reads, with the 95% confidence intervals being 78,115 and 86,322 reads (Supplementary Figure 3). About 97.02% of the reads were annotated as Bacteria, 2.43% as Archaea, and 0.39% as Eukaryota. Averaging across libraries from the same sediment ultimately yielded 150 community tables comprising 66,230 ASVs, 1,703 genera, and 78 phyla available for machine learning. Optimization of the hyperparameters was performed using the validation set with taxa at ASV rank. The achieved validation and prediction scores were averaged over the six training/test splits for each data set.

**FIGURE 1:**
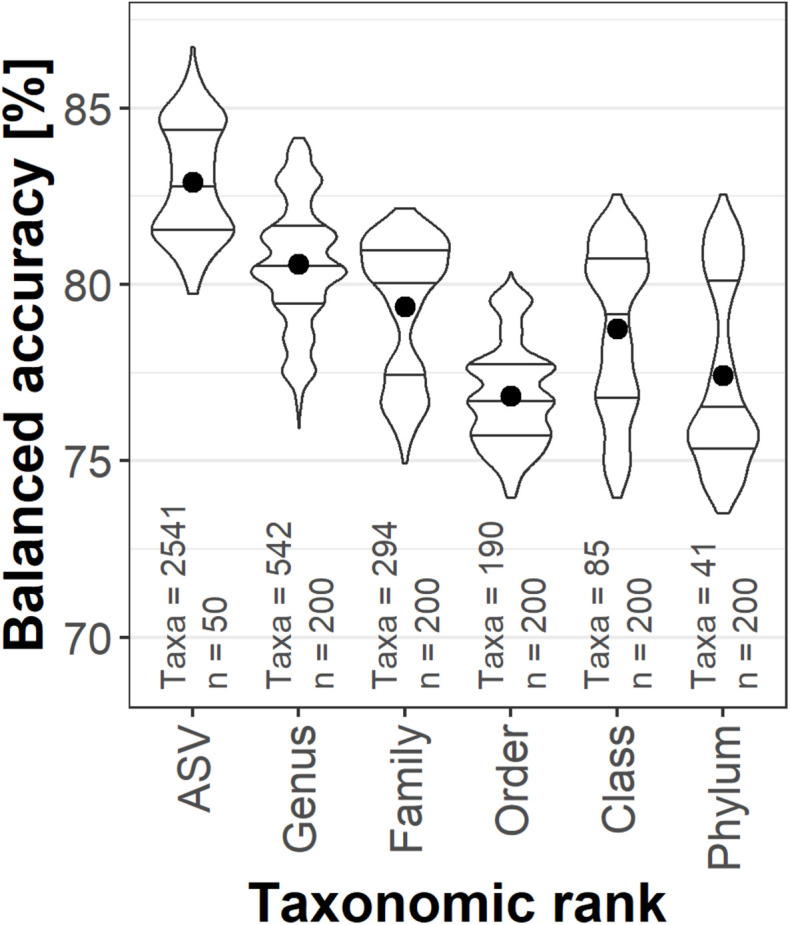
Violin plots with median and interquartile range of correct TNT classifications of the validation set. The relative abundances were agglomerated on the taxonomic ranks. The dot represents the mean balanced accuracy; the classification results of the six different data set splits were averaged. n indicates the number of models calculated, Taxa represents the number of variables for each rank.

Taxonomic ranks were then compared for their potential to predict the presence of TNT. The hierarchical structure of the taxonomic annotation allowed investigating the influence of pooling the relative abundance by taxonomic ranks to identify the best compromise between the number of taxa and the information contained in inter-taxa abundance variability (Figure 1). The highest mean balanced accuracy was achieved by ASV (82.9%) and decreased toward the broader order rank (74.9%). Training with relative abundance per class (78.8%) and phylum (76.9%), however, still resulted in acceptable predictions, yielding more accurate classifications than on order rank. The genus rank (80.6%) was chosen for further analyses; a compromise between the best accuracy, reduced number of variables and the possibility to add community compositions from other sources, as ASVs are unique to this data set.

**FIGURE 2:**
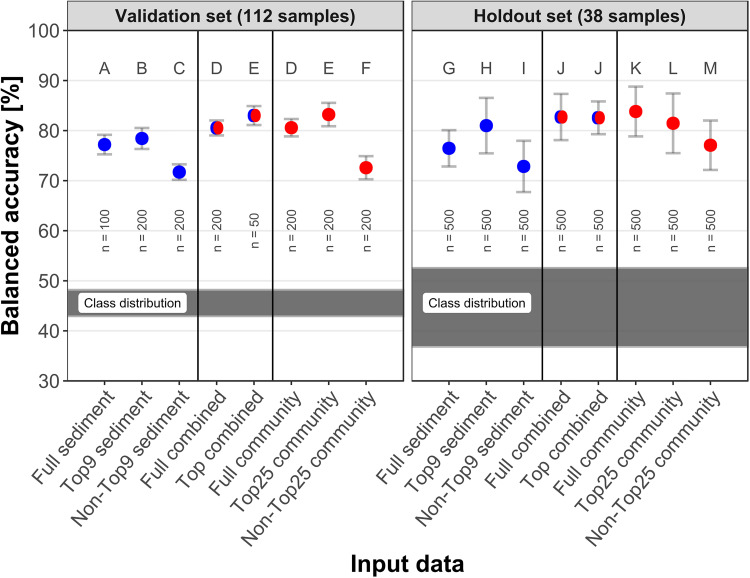
Correct TNT classifications per input data in the validation and holdout test set. Red indicates community data, blue symbolizes sediment data and red-blue combined variables. Of each data type, either all variables were utilized by the model (“Full”), or only the best variables based on variable importance (“Top”) or all variables except Top (“Non-Top”). Classification performance is displayed as mean and standard deviation of balanced accuracy, the classification results of the six different data set splits were averaged. The validation values are out-of-bag estimates. The letters indicate which groups were significantly (adjusted *p* < 0.005) different to all other groups within the data set. The shaded area indicates the distribution of samples containing TNT among the six data set splits. n indicates the number of models calculated.

A selection of eight input data sets was utilized for TNT prediction (Figure 2). “Full sediment” contained 41 independent environmental variables and “Full community” included 542 genera (applying a 0.08% relative abundance threshold). The mtry factor 5 allowed for 115 genera being considered at each node. The 0.08% threshold yielded the second highest mean balanced accuracy among the examined threshold values, and showed a more distinct classification distribution (Supplementary Figure 4), therefore it was applied to all community data sets presented here.

With reference to the validation set, selections of either the most important 25 genera or 9 sediment parameters yielded more accurate classifications than using all variables; the lowest scores were achieved by using the remaining non-important variables. In this order, the mean balanced accuracy for sediment data decreased from 78.4 over 77.2 to 71.7% and for the community data from 83.2 over 80.6 to 72.6%. Using the most important variables from both data sets combined also improved the classification from 80.5 to 83.0%. The “Top25 community” represents 4.6% of the genera and increased the balanced accuracy, whereas the other 517 genera significantly reduced it. For each variable selection (Full, Top, and Non-Top), the community data performed better than the corresponding sediment data. The combined input data achieved classifications similar to community data alone.

2,4,6-Trinitrotoluene was present in 44–48% of the samples in the six training data sets. The holdout set contained fewer samples; consequentially one sample’s classification represented >2.5% accuracy. This led to more widespread class ratios, from 36 to 52%, and thus a higher standard deviation. Best predictions reached 83.8% with “Full community” and 82.7 and 82.6% with “Full combined” and “Top combined”, respectively. Predictions on the holdout set were slightly better than the corresponding validation scores, except for “Top combined” and “Top genus”. The largest difference between validation and holdout scores was an increase of 4% for “Non-Top25 community”. Validation and holdout scores met the same range from 70 to 85%.

The means of the balanced accuracies in the validation set were significantly different from each other (adjusted *p* < 0.005) except “Full community” to “Full combined” (D) and “Top25 community” to “Top combined” (E) in the validation set. This extended to all groups in the holdout set except for “Full combined” to “Top combined” (J).

**FIGURE 3:**
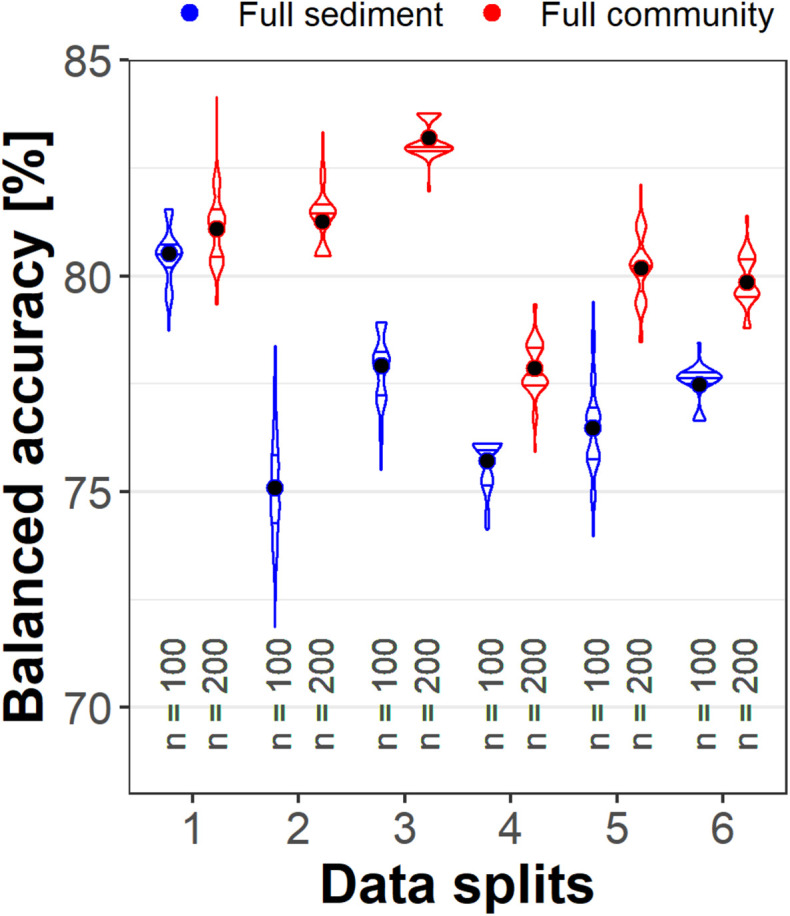
Violin plots with median and interquartile range of correct TNT classifications for six different validation sets. Full community (red) always performed better than Full sediment (blue) and their performances changed independent of each other toward the different validation set compositions. The dot represents the mean balanced accuracy; n indicates the number of models calculated.

The distribution of information among samples was then assessed by comparing the validation scores for the six training data sets. The results showed that “Full community” was more accurate for each set (Figure 3). Scores varied by up to 5% between the datasets for “Full sediment” (75.1–80.5% mean balanced accuracy) and “Full community” (77.9–83.2%), but the variation was not well correlated between sediment and community data. For example, comparing data set 1 and 2, the “Full sediment” classification performance dropped whereas the “Full community” balanced accuracy was maintained. These findings signal that the available sediment parameters and taxa abundances did not supply equivalent information.

### Grain Size Distribution as the Major Driver of Community Composition

**FIGURE 4:**
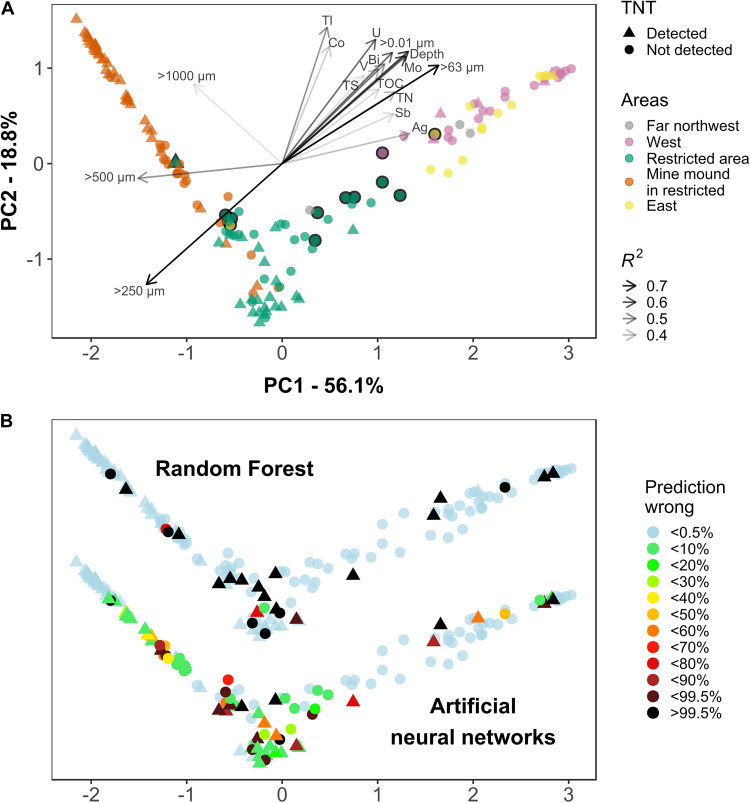
Principal component analysis (PCA) ordination based on the abundance of the most important 25 genera. Dissimilarity between samples was calculated using the proximity matrix of an unsupervised random forest. **(A)** The microbial communities were colored by sample area and shaped to indicate the presence of TNT. The length and shade of correlating sediment parameters (*p* < 0.001, *R*^2^ > 0.3) represents the goodness of fit. The black outline marks East (yellow) and West (purple) samples which were not multicorer samples. Similarly, the outline marks Restricted Area samples that were not part of a transect. **(B)** Using the same ordination as in panel **(A)**, the fraction of misclassifications per 1,000 (RF, top) and 999 (ANN, bottom) predictions is displayed for each sample. Light blue colored samples were always correctly predicted, black displays consistently misclassified samples. Please note: the *y*-axis [PC2 in panel **(A)**] was stretched to accommodate the results from both methods.

After successful classifications were achieved using community information, TNT was investigated with regard to its potential as important driver of the microbial community composition; as such influence would facilitate the process of prediction. PCA ordination of the Top25 community was performed using the sample proximity obtained by an unsupervised RF classification. PC1 explained 56.1% variation. Along PC1, the grain size fractions <125 μm were separated from those >250 μm (Figure 4A). The latter spread along PC2, which explained 18.8% variation. The former fractions co-correlated with further sediment parameters; some of those were important variables for RF when using Full sediment (vanadium, cobalt, and total nitrogen). Significant correlations with munition compounds were not found. The highest accordance among munition compounds with the community composition ordination was shown by 2,6-DNT with *R*^2^ of 0.033 and *p* of 0.07. TNT (*R*^2^: 0.014, *p*: 0.38) was detected across all clusters, but predominantly present in mine mound samples. Only a few core samples contained TNT.

The multicorer samples comprised smaller sized particles than most surface sediments. They were sliced at 2 cm, from the sediment-water interface to 22 cm depth (Figure 4A, West and East areas, no black outline) and formed a prominent cluster, with communities driven by the grain size distribution and presumably the redox potential declining with depth. The region did not play a role for clustering, as cores were collected kilometers east and west of the mine mound, which itself is centrally located in the restricted area (Supplementary Figure 1).

The samples from the mine mound area (a cluster of about 70 mines) were mostly taken within a defined distance of 0–5 m to a mine. Although this is a part within the restricted area, the communities mostly grouped together. Several transects with sampling intervals of 20 m were conducted across the restricted area, surrounding the mine mound (Figure 4A, Restricted Area, no black outline). The corresponding communities formed a distinct cluster, too. Three more samples with no detected TNT were collected multiple kilometers away toward northwest. To validate these results using a more traditional approach, the same data was subjected to a non-metric multidimensional scaling (nMDS) using Bray–Curtis dissimilarity, which lead to the same conclusions (Supplementary Figure 5).

An ordination based on only the sediment parameters including the munition compounds was generated to compare with the microbial community ordination. Again, no separation based on TNT presence was observed (Supplementary Figure 6). Furthermore, the mine mound and the overall restricted area sediments clustered alongside, with eastern samples placed in proximity. In this ordination the multicorer samples to the east and west were clearly separated, with west and far northwest samples forming a remote cluster.

The seasonal conditions during sampling should be mentioned, as they might have influenced the community composition more strongly than the sediment parameters. The restricted area was sampled mostly manually in June and September 2017 at the sediment surface by divers; three more sediments were obtained using Van Veen grab samplers. The mine mound samplings by divers took place in December 2016 and November and December 2017, which could explain the division between mine mound and restricted area microbial communities. The cores were collected on 1 day in October 2017.

Random Forest was able to predict TNT using only sediment parameters, although no driving influence by munition compounds were detected in the ordinations. Therefore, Spearman rank-order correlation was performed to investigate which variables significantly (*p* < 0.01) correlated with TNT. A cluster of munition compounds consisting of TNT and its metabolites 2-ADNT, 4-ADNT, 2,4-DANT, and 2,6-DANT was identified, which also showed a loose positive correlation with RDX (Supplementary Figure 7). Another cluster consisted of DNB, HMX, TNB, 2,4-DNT, and 2,6-DNT. The latter two munition compounds are co-contaminants of TNT. However, the munition compounds were not part of the RF input data set. Furthermore, some weaker correlations with TNT were identified.

The results confirmed that community compositions were primarily controlled by factors other than the presence of TNT; therefore, supervised classification was applied to still extract such a potential impact. Both community and sediment data-based ordination demonstrated as well, that the distribution of TNT containing samples was appropriate to utilize machine learning.

### Community Information Important in Combined Data Sets

Foregoing results indicated that a potential impact of TNT was masked by stronger drivers. Therefore, it was essential to investigate the variables that enabled RF predictions. Potential microbial fingerprints (in case of community data) indicative for the presence of TNT were examined. The variable importances, extended by maximal relative abundances and taxonomic lineage of the genera are provided in Supplementary Figure 8.

**FIGURE 5:**
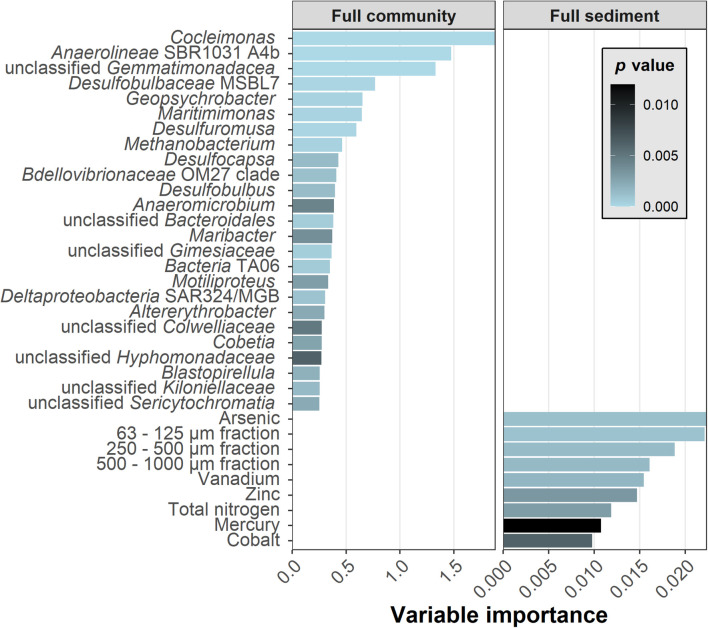
The variable importance and *p*-values for the classification of TNT presence. Twenty-five genera of the Full community and nine sediment parameters of Full sediment were selected. The most detailed taxonomic annotation was provided in case none was available at genus rank. Importance and *p*-values were generated after Altmann (Full sediment) and Janitza (Full community) for six data splits and subsequently averaged.

The most supportive genera (Figure 5) were *Cocleimonas* (1.65% maximal relative abundance), the unclassified Anaerolineae SBR1031 A4b (0.11%) and an unclassified Gemmatimonadaceae (0.38%). Relative abundances of the Top25 genera ranged from 5.65% for the unclassified Cyanobacterium Sericytochromatia to 0.09% for the unclassified Planctomycete Gimesiaceae. The important sediment variables contained grain size fractions, elemental contents, and total nitrogen as a sum parameter for various nitrogen compounds. Among these, arsenic and the 63–125 μm fraction were most important. This grain size fraction correlated with sum parameters of sulfur and carbon and element contents of e.g., molybdenum and uranium in direction of the multicorer samples.

The 50 most important Full combined variables were then compared against the foregoing top Full community and Full sediment variables. Interestingly, out of 50 variables only 6 were sediment parameters [arsenic (#9), zinc (#21), 63–125 μm fraction (#35), vanadium (#40), mercury (#45), and cobalt (#48)], all of them were part of the Top9 sediment. The achieved classification score of Full combined was as accurate as by Full community input (Figure 2). The 44 genera included all of the Top25 community genera. Further genera were related to them on family or order level, for example Flavobacteriaceae, Clostridiales, Sphingomonadaceae, and Desulfobulbaceae. Overall, recovered variables in the combined data set were as important as in individual data sets. Sediment importance ranking concurred, although they were calculated using two different methods for Full community and Full combined.

### Processing of All Samples Depends on a Combination of Important Variables

To understand the model’s approach to classify the samples and to validate a potential indicative fingerprint, the reasons for the determination of important variables had to be identified. By analyzing their relative abundances, it became clear that 23 of 25 important genera were in average more abundant in surface than core samples, the opposite was true for the clostridium *Anaeromicrobium* and TA06 (Supplementary Figures 9I,Y).

Although the abundance of the most important *Cocleimonas* could be very low in samples regardless of class, it mostly occurred in samples with TNT. Second most important *Anaerolineae* SBR1031 A4b proved to be more abundant overall in samples with TNT. Clade TA06, however, was found in as few as 12 samples, and was abundant in very similar sediments of both classes (Supplementary Figures 9H,P,Y). The presence of some genera was linked to grain sizes: *Cobetia* was present in medium to finer sediments, *Colwelliaceae* on the contrary in coarser samples (Supplementary Figures 9C,G). This goes along with the finding that in a combined data set the grain size information was not as important anymore. But other important genera such as the up to 4.1% abundant *Maribacter*, *Maritimonas* (3.5%), and *Blastopirellula* (4.6%) were present in 131–142 of 150 samples (Supplementary Figures 9B,D,E). In a similar fashion, the concentrations of sediment parameters were displayed in Supplementary Figure 10.

### RF Predictions Were Consistent, With Transect Samples Being Most Challenging

With achieved classification scores for the presence of TNT well above 80% the inner works of the model for the important variables became understandable, but additional information on misclassified samples was required. By recording the mean of 1,000 predictions, it was possible to identify consistently and/or incorrectly classified samples (Figure 4B).

Random Forest had cumulatively 24 of 150 samples misclassified (84% accuracy), including 5 of 35 core samples and 6 of 58 sediments near the mine mound. These predictions were robust; a classification was either wrong or correct, taken 0.5% tolerance into account. Only four samples showed varying classifications, being incorrectly classified 1.3, 71, 79, and 93% of the time.

A PCA ordination based on a TNT classifying model showed the attempt to cluster by class: clusters in top right and bottom center were predominantly TNT-present and in the top left mostly TNT-absent (Supplementary Figure 11). The center region displayed communities of both classes intermingled. Samples of all areas were observed there, but those from the restricted area were most present with both classes. The samples in the center region were more often misclassified, mostly predicted as TNT-absent. Finding two separate clusters for TNT-present samples indicated that two distinct groups of important variables contained in the model were required to achieve classifications of those samples.

The restricted area achieved the highest misclassification rate. Within a total of 51 sediments for this region, all 13 misclassifications could be attributed to 41 samples collected by four transects (Supplementary Figure 11, Restricted area, no black outline, see also Figure 4A). The 200 m long transects, each consisting of 9–11 sampling points, covered different sections of the restricted area.

In general, the less abundant class in a given region is prone to misclassification; however, minority class samples were also predicted correctly. The inconsistently classified samples can be imagined close to the decision boundaries between predominantly “present” and “absent” groups (Figure 4B, RF).

The robustness test utilizing an ANN gathered 70 wrongly predicted samples in 999 classifications. Sixty-four of those were not robust. More specifically, 30 samples were misclassified less than 10% of the time and another 11 samples were almost more frequently than 99.5% misclassified. Furthermore, all samples incorrectly classified by RF were misclassified by the ANN, too. Regarding the higher prediction variance of the ANN it should be noted that RF is an ensemble classifier (see section “Discussion”).

### TNT Metabolites Containing Samples More Likely to Be Classified False Positive

The presence of ADNTs or DANTs in sediments indicates that TNT had been present. It was hypothesized that such former TNT-containing sediments might harbor community compositions which “look like” TNT was still present after its dissipation due to resilience. In consequence such samples should be predicted falsely positive. A “clean” sample on the other hand contains neither TNT nor its metabolites, indicating that it was not contaminated with TNT for a longer time.

**FIGURE 6:**
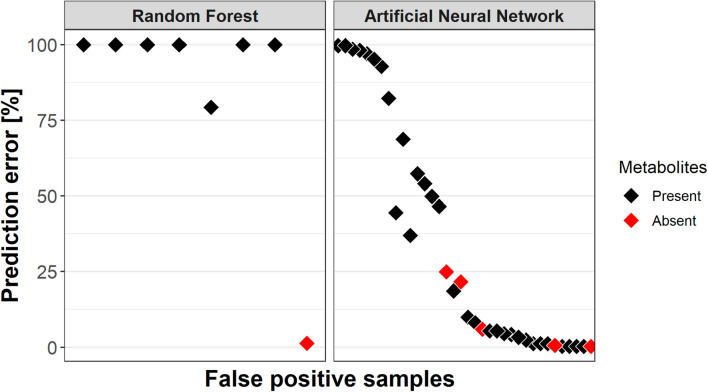
Misclassification rates of samples which were predicted “TNT present” but did not contain the explosive (False positive prediction). Red indicates whether a false positive samples contained TNT metabolites, i.e., ADNTs and DANTs. Samples containing metabolites were more likely to be misclassified as false positives.

The RF models predicted eight false positives; two of them were not consistently misclassified (Figure 6). Interestingly, seven of the false positives actually contained TNT metabolites and the one “clean” sample was only 1.3% times incorrectly classified. The ANN predicted 36 false positives, 5 of those without metabolites. Their prediction errors ranged from 0.3 to 25% with an average of 10.6%, compared to a mean prediction error of 38.3% for the remaining false positives with metabolites. Furthermore, prediction rates for false positives did not correlate with the individual or summed concentration of TNT metabolites.

It was additionally verified whether a higher TNT concentration goes together with a stronger impact on the community composition, thereby decreasing the probability of a false negative prediction. However, the RF predictions contained only two suitable false negative samples. For ANN a higher TNT concentration did not lead to better prediction rates of the sample.

## Discussion

In this study, microbial communities were used to predict the presence of TNT in sediments (at pmol⋅g^–1^ levels) in and around a munitions dumpsite in the German Baltic Sea with about 84% balanced accuracy. Genera and sediment parameters being most important to reach this value, and the samples that were a challenge to the models, could be identified. Moreover, many TNT false-positive samples had traces of TNT metabolites, indicating that microbial community compositions may conserve information of former TNT presence for a longer period.

### Model-Relevant Genera Were Related to TNT-Degrading Taxa

A selection of 9 sediment parameters or 25 bacterial genera predicted TNT as well (holdout set) or even better (validation) compared to using all variables. This is a result similar to the results of Thompson et al. (2019), who conducted a study to predict concentrations of dissolved organic carbon using most effectively a subset of the microbial community compositions of a plant litter decomposition experiment. One reason for such improved performances could be a lower likelihood of overfitting.

The subset was identified by the variable importance metric, which indicates correlation with the response variable. A potential causation between TNT presence and identified important genera is attributable to TNT as a source for biomass generation, energy supply or toxic stress (George et al., 2009; Gallagher et al., 2010). The bacterial enzymatic degradation of TNT is mediated by nitroreductases. Nitroreductases and other common enzyme families have been reported as responsible for the reduction of nitro groups (Esteve-Núñez et al., 2001), which are among the first steps of microbial TNT transformation. Such enzymes are widely distributed among microorganisms, rendering microbial TNT metabolization possible in marine sediments (Roldán et al., 2008). In fact, TNT degradation products as ADNTs and DANTs were present in Kolberger Heide sediments. The ability to degrade TNT was specifically proven for more than 20 different genera, ranging from anaerobic members of the family Clostridiaceae to aerobic members of the family Pseudomonadaceae (Esteve-Núñez et al., 2001). Relatives of these organisms are important for the models of our study; for instance, the Top25 and Top50 members Anaeromicrobium and Clostridiaceae sensu stricto 13, respectively, are phylogenetic members of the Clostridiaceae. Top25 *Altererythrobacter* is also phylogenetically related to TNT-degrading *Sphingomonas sanguinis* (Habineza et al., 2017). However, deriving bacterial activities from phylogenetic relations has to be handled carefully as phylogeny can be an unreliable indicator of bacterial ecology, although tools like PICRUSt2 demonstrated the prediction of the functional potential of a 16S rRNA gene-derived community profile (Douglas et al., 2020). Thus, it is also possible that the obligate anaerobic *Anaeromicrobium* acted as redox indicator for reduced conditions in the investigated sediments or that the abundance of the identified taxa would also have correlated with the presence of naturally occurring aromatic compounds.

It was furthermore shown that multiple genera were required to separate classes in all samples, because some important taxa, such as clade TA06, were only detected in 12 samples. Consequently, their contribution to classification was limited. However, these genera were likely important, because they allowed classification of otherwise similar samples. In this regard, other variables could not replace this information.

The prediction of TNT was still successful using the available sediment information alone. We assume that, in this case, many samples were separated first and foremost by the grain size distribution, as the finer multicorer samples contained many TNT-free sediments, compared to the coarser mine mound samples, consisting of many TNT-contaminated sediments. The other parameters further on separated within those groups specifically. In order to supplement microbial community variables one might intuitively assume that at least grain size and, where appropriate, redox conditions should be measured as major proxies to inform the model. However, the combined usage of community compositions and sediment parameters did not lead to predictions more accurate than by using the community data on its own. It turned out that the second most important Full sediment variable (63–125 μm grain size fraction) was only the 35th most important Full combined variable and the other grain size fractions were not included in the Top50. These findings show that taxa abundances can replace the grain size information because it is reflected by the community data.

More information would be required to conclusively determine the reason why samples from the mine mound area, which is located in the center of the restricted area, formed a distinct cluster in the unsupervised PCA ordination (Figure 4A). This was noticeable, as the transect samples formed another distinct cluster, though the transects geographically encircled the mine mound. We suggest the sampling of the mine mound in a different season than the conduct of the transects as a main reason for varying assemblages (Meyer-Reil, 1983), but the proximity to mines as factor cannot be ruled out. Such an influence, however, was not displayed by the measured sediment variables (Supplementary Figure 6), where sediments from the mine mound and the restricted area clustered more similarly.

### The Microbial Fingerprint Requires Further Data to Become Indicative

A meaningful indicative microbial fingerprint is equivalent with the abundances of important variables per response class, if they are causally related. Yet the clade TA06 was detected in 12 of 150 samples, which increases the likelihood of being only coincidentally useful; in other words, the sample size is too small to know whether overfitting occurred (Dietterich, 1995). Thus, there is a need to reduce the potential of spurious relationships. To receive a reliable, generalizable and informative fingerprint we propose to: (a) maximize the sample to variable ratio by using a minimum number of taxa while still reaching acceptable predictions, e.g., using backward elimination (Guyon et al., 2002); (b) add samples of further targeted sites and conditions, which cover all response classes; and (c) perform regression instead of classification as long as the concentration of the response variable is appropriately distributed and covered. Regression yields a more informative relation between response and community composition and avoids arbitrary limits between response classes.

In our study, the 150 samples were split into six different training and test sets. The test set is usually the ultimate benchmark for the predictive potential of the model, but it was likely that not all samples in our data set were equally different from each other. Therefore, the hyperparameters as well as the important variables were based on averaged results from the six sample set compositions. This approach can be seen as extra layer of repeated cross validation and helps to maximize the generality of the fingerprint and the chosen settings. It also resulted in more reliable prediction accuracies, as for an individual sample split mean balanced accuracies >90% were achieved. Important is that by this approach a training sample of one split is also a test sample of another split. This results ultimately in information leakage, although in a rather indirect way (Kaufman et al., 2011). We argue that this approach is justifiable for our small data set, where the detection of a generalized TNT-indicative microbial fingerprint as proof of principle was the priority. But in larger data sets, or to compare different prediction methods, regular approaches with a fixed hold out test set should be applied. It should also be remembered that if such a model would be actually deployed, the data to be predicted, e.g., from the next sampling campaign, would not yet exist.

With regard to an indicative fingerprint, we conclude that the presented data set probably contains essential parts of it, but is not yet suited to distinguish accidentally valuable from truly influenced variables. However, we conclude that the first steps were successfully taken to determine a microbial fingerprint indicative of TNT contamination in Kolberger Heide.

### An Indicative Microbial Community Fingerprint May Differ Between Habitats

Given the existence of such a fingerprint, part of its value is to use it for other areas of interest. In this regard, the usage of microbial community compositions has both advantages and drawbacks. Advantageous is that the features were assigned at least a partial taxonomy; thus, are interpretable and relatable to literature or cultivation dependent complimentary investigations. Yet, using taxa infers using a proxy, depending on many influences such as nutrients, salinity, redox, pH, temperature (Lindh and Pinhassi, 2018), or as described in this study, grain size.

In order to create meaningful fingerprints, communities likely need to originate from a somewhat similar habitat under specific conditions. But, importantly, our models still could predict using data from various habitats—as from deeper multicorer and surface sediment samples—albeit the variable importance would be a mixture of habitat fingerprints and therefore less interpretable. Additionally, the important taxa might not occur everywhere. To address this issue, higher tax ranks can be used, which are more likely to be found in various areas. Ghannam et al. (2020), for instance, used phyla to differentiate geographic locations on global scale. In our spatially restricted samples the phylum rank also achieved 76.9% mean balanced accuracy, which is still well above coincidence. But the context of the response variable should be considered, as a higher taxonomic rank is reasonable to cover taxa globally. However, in a previous study we detected distinct reactions to the herbicide glyphosate at OTU-level for *Pseudomonas*, which were not distinguishable anymore on genus level (Janßen et al., 2019a). An alternative is to combine important variables from all taxonomic ranks and train with those. The usage of ASVs in this study would have led to slightly more accurate predictions, but the goal was to utilize a data set which could be amended by additional community compositions targeted with various primer sets or processed by other bioinformatic pipelines. The resulting additional taxa would be incompatible with the already generated ASVs, but could be combined on genus rank.

Furthermore, it is conceivable to target functions (genes or transcripts) directly by shotgun sequencing instead of using taxa as proxy. Alneberg et al. (2020) demonstrated that functional genes from metagenome assembled genomes predicted salinity and depth in Baltic Sea waters. For our study, metatranscriptomes would have been very helpful to identify the used (expressed) degradation pathways among the diverse functional potential of sediment communities. However, this approach would have required that the sediment samples were conserved for high-quality mRNA retrieval which, unfortunately, were non-existent.

### Misclassified Samples Define Further Sampling Campaigns

Two mechanistically different ML algorithms were able to predict the presence of TNT in Kolberger Heide sediments using 25 genera. The samples misclassified by RF were also misclassified by the ANN, indicating that the data were insufficient in that case, independent of the algorithm in use. RF worked directly with the relative abundances as input, a form of transformation or normalization could have had a positive effect on the prediction scores (Gloor et al., 2017). However, the performed z-score transformation for the ANN model input at least did not excel the RF predictions (Supplementary Figure 13). The more consistent predictions of RF stem in part from it being an ensemble classifier (Breiman, 2001). Thus, all the individual predictions of the tree models are not published, as they are for ANNs, but reduced to a single prediction based on a majority vote. As ANNs do not have this leveling mechanism by default, more variance in cumulated classifications was observed.

It seems reasonable to explore the microbial community composition by proximity matrix-based ordinations, using the same distance metric that is used for the supervised classification. It allows correlating environmental variables, the addition of context data and provides an understanding on the data set dynamics. Combined with the classification robustness it becomes a powerful approach to determine model limitations as well as their overcoming (e.g., more transect samples, Supplementary Figure 11). It can be compared to the supervised ordination, which indicates the separation by TNT presence or absence and confirmed that many of the samples consistently misclassified were not well separated. For more insights, decision boundaries can be added [for one model at a time (Hastie et al., 2009)].

### Resilience of TNT Presence as a Tool to Detect Historical Contaminations

In addition to investigating whether the composition of microbial communities can indicate TNT-contaminated sediments, it was of interest to us whether these indications could be maintained for a longer period of time, even if the sediment only contained TNT metabolites or was already TNT-free again. In this case, samples would be characterized as being false-positive. Indeed, based on our approach it became apparent that especially samples containing no metabolites at all had a lower chance of a false positive prediction. Unfortunately, the sample size did not allow a meaningful test of significance yet. The possible implications are relevant though, as shown by Smith et al. (2015), who successfully classified microbial communities affected by the Deepwater Horizon oil spill. Their RF models classified samples falsely positive, which were once contaminated, yet subsequently the hydrocarbon concentrations had returned to background levels.

To investigate such a phenomenon based on ecological resilience (Shade et al., 2012) at Kolberger Heide, it should be considered whether TNT and its metabolites result in similar variable importance and fingerprints due to their structural similarity as nitroaromatic compounds. In such a case, a test of true resilience after a TNT contamination—and therefore the time span to detect such—would require to work with once contaminated samples then free of TNT and its metabolites. Another reason for a prolonged taxa detection after TNT has been degraded could be a metabolic shift toward other carbon and energy sources. Such a shift from denitrification to fermentation was described by Orsi et al. (2017). Methodologically, it should also be ensured that the TNT metabolites were not formed e.g., in the water column and subsequently adsorbed to the sediment.

### Importance of Microbiological Surveys as a Key Component in Environmental Monitoring

The Kolberger Heide munitions dumpsite is a stressor to blue mussels (*Mytilus edulis*, Strehse et al., 2017; Appel et al., 2018) and dab (*Limanda limanda*, Koske et al., 2020); our study verified the presence of explosives and their transformation products in sediments as well. Furthermore, mines at Kolberger Heide have been proposed as point sources of mercury due to, e.g., mercury(II) fulminate fuses (Bełdowski et al., 2019). However, despite spottily occurring concentrations up to 4,503 μg Hg⋅kg^–1^ dry sediment, no correlation with the distance to mines was detected (Supplementary Figure 12). Additionally, most mines on-site are registered as discarded munition material (Kampmeier et al., 2020). In comparison to unexploded ordnance, those were not fused and therefore should not contain mercury(II) fulminate.

2,4,6-Trinitrotoluene was found strongly correlated with DANTs and ADNTs, though (Supplementary Figure 7). The presence of TNT metabolites proves that Kolberger Heide also represents a disturbance toward the microbial community, as it reacted to the explosives. But it is not clear yet to which extent the community is affected. A potential impact of TNT was surpassed by the main driving grain size distribution and correlating factors, which is expected for such low levels of TNT. Wikström et al. (2000) reported small amounts of degradation and increased microbial growth following the addition of TNT to lake microcosms. However, they did not find a permanent alteration of microbial communities based on random amplified polymorphic DNA analysis. In a study evaluating the toxicity of Harz soil extracts containing TNT, the *Aliivibrio fischeri* luminescence test (EN ISO 11348) reported a long-term EC_20_ of 60–90 ng⋅g^–1^ or 264–396 pmol⋅g^–1^ [assuming 1 mL = 1 g (Frische, 2002)]. Such concentrations were met in the Kolberger Heide in exceptional cases, e.g., at the detonation site. A summary of various studies investigating a disturbing or toxic impact on soil microorganisms can be found in the article of Kuperman et al. (2009), although effects were only observed at soil TNT content 10^3^–10^6^-fold higher than measured in the current study.

The information of a potential munition compounds impact could have been recorded by microbial communities. Such information could be utilized in cases were direct measurements are problematic to realize: it was reported that TNT is hard to detect just in centimeters distance from containments because it slowly dissolves but is rapidly transformed or bound to sediment (Porter et al., 2011; Gledhill et al., 2019). In fact, TNT can be bio-transformed in minutes (Elovitz and Weber, 1999). Therefore, measured TNT concentrations may not fully capture the impact toward the environment and the microbial community specifically. Furthermore, it should be kept in mind that many more sediments contained munition compounds other than TNT; the impact on the environment has to be considered for all munition compounds in terms of combined effects and quantity, especially with the background of continuously corroding of metal housings. There is even an urgent demand to merely identify the actual munition compounds composition of the dumped ammunitions (Beck et al., 2019). The release of munition compounds might also be intermittent (“sudden release”), which emphasizes the advantages of a resilient indicative fingerprint.

We suggest that microbial community data should be included with monitoring strategies and could potentially act as an information repository to complement the snapshot which is generated by standard monitoring methods. In return, monitoring provides a standardized solution to retrieve more and even specifically required samples to overcome the most severe hindrance for ML: limited sample size. With sufficient data, supervised machine learning could identify impacts of contaminants without being main community drivers. Depending on available context information, the sequenced community data can be utilized to train for further variables as Smith et al. (2015) demonstrated, when they predicted 18 highly significant and 8 significant geochemical features such as element concentrations or conductivity of groundwater wells using community data. In this study, the munition compounds showed challenging and partly correlated distributions with strongly imbalanced classes, therefore, the prediction of other compounds than TNT led to overoptimistic results. However, the important consequence is that a single community composition can be utilized as source of information on potentially all relevant shaping environmental factors. This affects the cost/benefit ratio, where the costs for the applied munition compound detection method alone are in the same order of magnitude as those for 16S rRNA amplicon sequencing. An extensive discussion on the opportunities of including sequencing methods into monitoring strategies and a cost comparison for analytical and sequencing methods are presented in Janßen (2020).

It should be reminded, that the herein presented results were achieved using a Kolberger Heide site-specific data set. The models generated based on these data sets do not apply to other geographical areas, yet. However, this limitation is solely due to sample size and distribution and justifiable for a proof of principle. By producing more data from different geographic areas, target compounds and habitats, models can be trained for e.g., the detection of TNT in the Baltic Sea. These models then could be used for monitoring as described above. With regard to munitions, the problem of sea-dumped and leaking munition is not restricted to the Kolberger Heide, yet rather a global problem (Strehse et al., 2021) that already affects humans *via* incorporation into the marine food chain (Maser and Strehse, 2021).

## Conclusion

This study demonstrated successfully the prediction of TNT presence in Kolberger Heide sediments using microbial community information, and highlighted regions of the munitions dumpsite where further samples should be collected. A possible TNT indicative fingerprint on genus rank was identified as successful proof of principle. Finally, a potential for TNT-dissipation resilient community compositions was observed.

The importance of environmental monitoring including the implementation of the aforementioned approach was laid out, harnessing its predictive potential. In this regard, resilient microbial communities would allow to fill gaps between sporadic samplings; thus, to identify contamination events not measurable at all times. As surplus, each monitoring event would generate more training data for more accurate predictions. This may ultimately lead to a more fundamental monitoring of marine ecosystems; based on highly resolved biological variables and potentially automatable or autonomously operable.

## Data Availability Statement

The datasets presented in this study can be found in online repositories. The names of the repository/repositories and accession number(s) can be found in the article/Supplementary Material.

## Author Contributions

RJ performed the molecular and geological lab work (except munition compounds measuring), acquired funding, planned the data analysis, performed the bioinformatic processing, wrote the analysis scripts, wrote the R package, conducted the machine learning and analyzed the results, and wrote the first draft of the manuscript. AB and EA measured the munition compound concentrations, designed the sampling campaigns at Kolberger Heide, and commented on the manuscript. AB discussed the data and provided a great amount of munition compounds specific knowledge. JW supported the bioinformatics analysis, performed code review, supervised the R package development, provided the IT infrastructure, and commented on the manuscript. JA guided the initial bioinformatic analysis and provided statistical and general coding support, and commented on the manuscript. OD supervised the geochemical analysis and conducted ICP-MS measurements, interpreted the data, and commented on the manuscript. CB connected the various subprojects to make this study possible, provided historical and jurisdictional context, acquired funding, addressed the monitoring requirement, and commented on the manuscript. BK provided the 16S rRNA gene amplicon sequencing device and materials, and commented on the manuscript revision. EM acquired funds and samples and provided a toxicological assessment, and commented on the manuscript revision. AA came up with essential ideas regarding the analysis of microbial communities by machine learning in an ecologically meaningful way, supervised bioinformatics and statistical data analysis discussed the data, and commented on the manuscript. ML conceived the concept, supervised the analyses, discussed the data, acquired and provided funding, rewrote parts of the manuscript, and commented on the manuscript. All authors have read and approved the final version of the manuscript.

## Conflict of Interest

The authors declare that the research was conducted in the absence of any commercial or financial relationships that could be construed as a potential conflict of interest.

## Publisher’s Note

All claims expressed in this article are solely those of the authors and do not necessarily represent those of their affiliated organizations, or those of the publisher, the editors and the reviewers. Any product that may be evaluated in this article, or claim that may be made by its manufacturer, is not guaranteed or endorsed by the publisher.

## Footnotes

1
https://github.com/RJ333/phyloseq2ML

2
https://github.com/RJ333/Kolberger_Heide_manuscript
