# Supplementary material for: Machine Learning Predicts the Presence of 2,4,6-Trinitrotoluene in Sediments of a Baltic Sea Munitions Dumpsite Using Microbial Community Compositions
Source: Front Microbiol. 2021 Sep 29;12:626048. doi: 10.3389/fmicb.2021.626048 (PMC8513674; doi:10.3389/fmicb.2021.626048)
Supplement: Supplementary file 1 [file Data_Sheet_1.zip › Supplements_update_09_28/Supplementary_Figure_01_Map_KolbergerHeide_samplings_noline.docx]

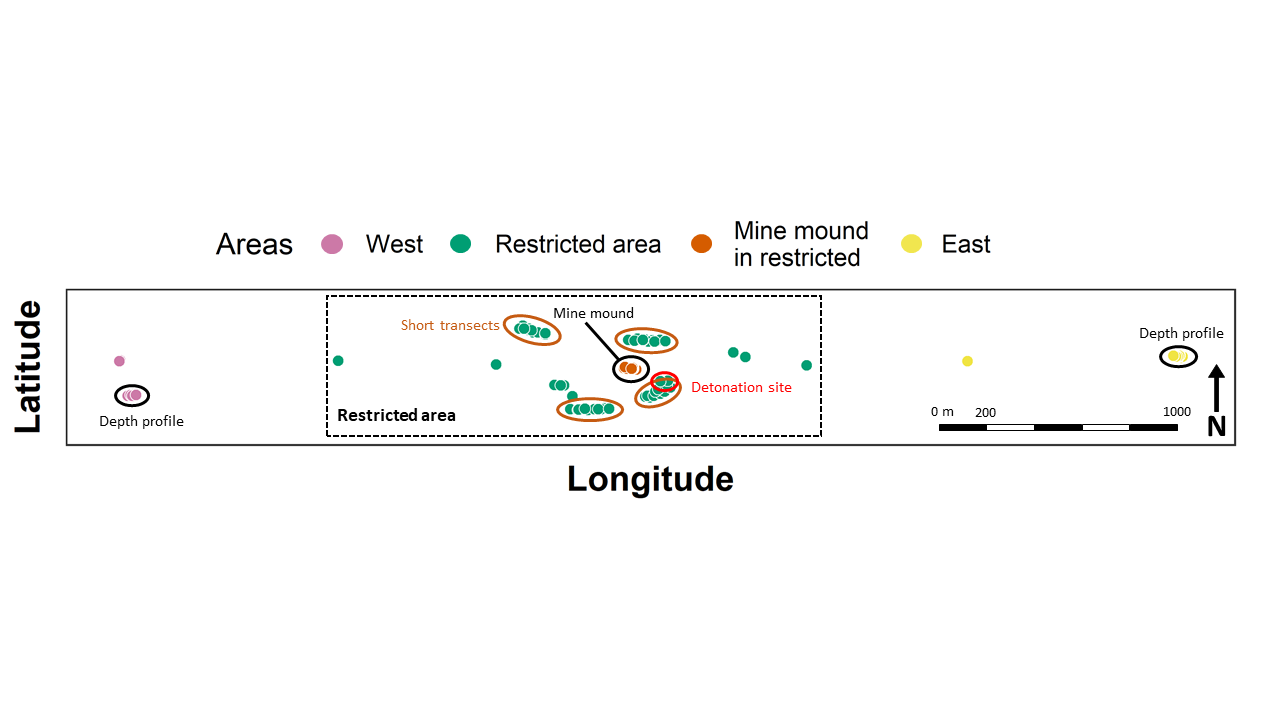


Supplementary Figure 1: A map of the sampling sites at Kolberger Heide munitions dumpsite, located in the Baltic Sea near the city of Kiel, Germany. The restricted area is demarked by a dashed box. The multicorer sampling took place at the sites named “Depth profile”. Sampling sites featured in the study within the restricted area were the short transects of 200 m total length, with samplings every 20 m around the mine mound. The mine mound was subject to several sampling campaigns, including the sampling in defined distances to an individual mine. Craters caused by detonation of munition are located at the “Detonation site”, munition compound concentrations at this site were about 1000 times higher than in average. For more details the reader is referred to Kampmeier et al., (2020).

Kampmeier, M., van der Lee, E. M., Wichert, U., and Greinert, J. (2020). Exploration of the munition dumpsite Kolberger Heide in Kiel Bay, Germany: Example for a standardised hydroacoustic and optic monitoring approach. *Cont. Shelf Res.* 198, 104108. doi:10.1016/j.csr.2020.104108.
