# Supplementary material for: Machine Learning Predicts the Presence of 2,4,6-Trinitrotoluene in Sediments of a Baltic Sea Munitions Dumpsite Using Microbial Community Compositions
Source: Front Microbiol. 2021 Sep 29;12:626048. doi: 10.3389/fmicb.2021.626048 (PMC8513674; doi:10.3389/fmicb.2021.626048)
Supplement: Supplementary file 1 [file Data_Sheet_1.zip › Supplements_update_09_28/Supplementary_Figure_04_ASV_Threshold_noline.docx]

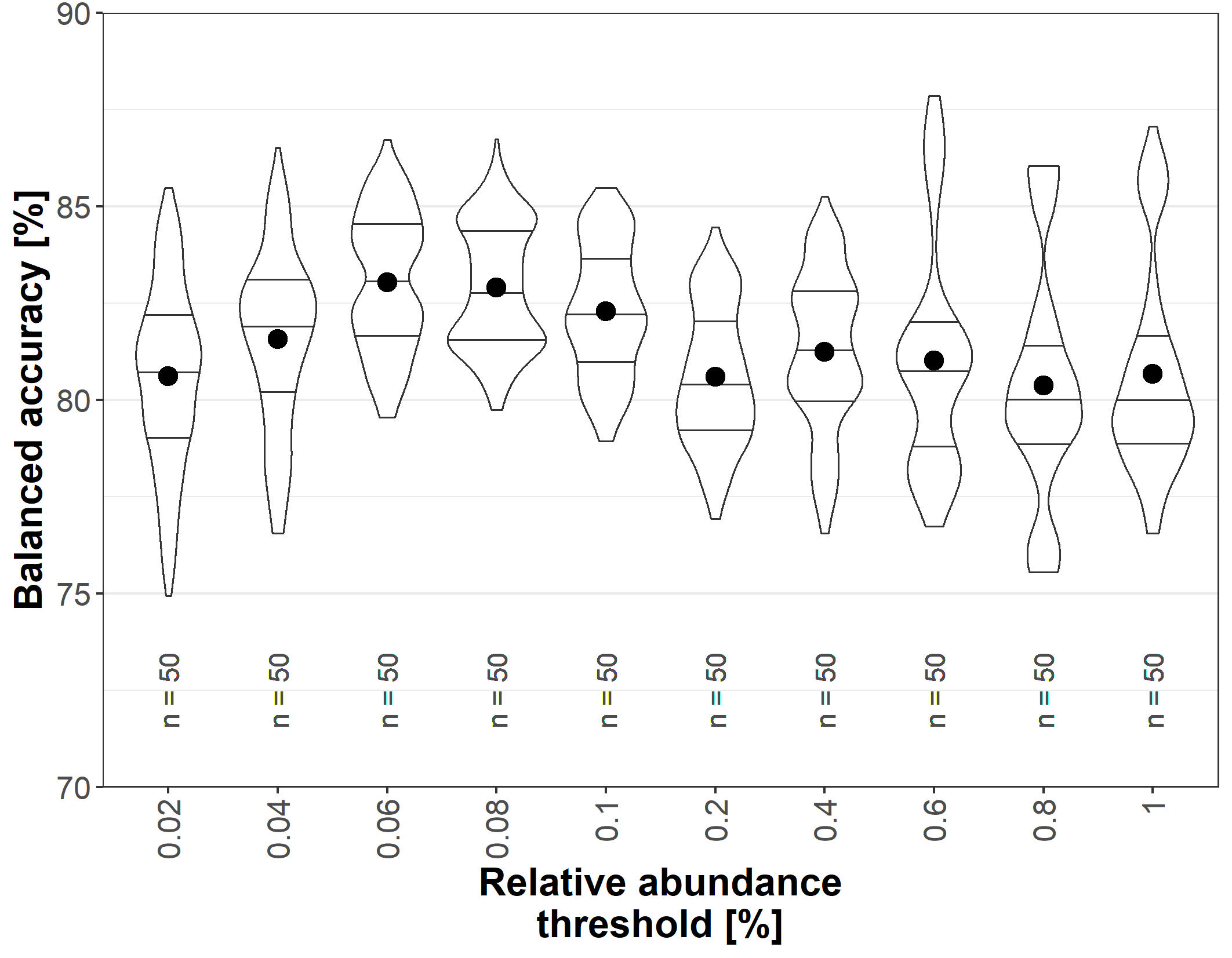


Supplementary Figure 4: Violin plots of correct TNT classifications using different thresholds on relative abundance per ASV for the validation set. The dot represents the mean balanced accuracy, averaged over six different data set splits. n indicates the number of models calculated. The random forest models consisted of 10000 trees with an mtry factor of 5. The mean balanced accuracy ranged from 80.4 – 83.0%. 0.08% was chosen because the distribution became more distinct compared to the slightly better performing 0.06% threshold.
