# Supplementary material for: Machine Learning Predicts the Presence of 2,4,6-Trinitrotoluene in Sediments of a Baltic Sea Munitions Dumpsite Using Microbial Community Compositions
Source: Front Microbiol. 2021 Sep 29;12:626048. doi: 10.3389/fmicb.2021.626048 (PMC8513674; doi:10.3389/fmicb.2021.626048)
Supplement: Supplementary file 1 [file Data_Sheet_1.zip › Supplements_update_09_28/Supplementary_Figure_05_nMDS_ordination_25_genera_noline.docx]

Supplementary Figure 5:

To compare the PCA ordination of a Random Forest-generated proximity matrix with a more traditional community compositions analysis, a non-metric multidimensional scaling (nMDS) analyses was performed based on Bray–Curtis dissimilarities. The ordination with the lowest stress was determined based on 50 runs. Relative abundances were used as input, square-root-transformed and Wisconsin double-standardized. To identify microbial community shaping influences in the nMDS ordination, the sediment parameters were correlated. The function *envfit()* from R package vegan v. 2.5-6 (Oksanen et al., 2019) with 9999 permutations was used to achieve this. Correlating parameters with *p* < 0.001 and *R*² > 0.3 were displayed.


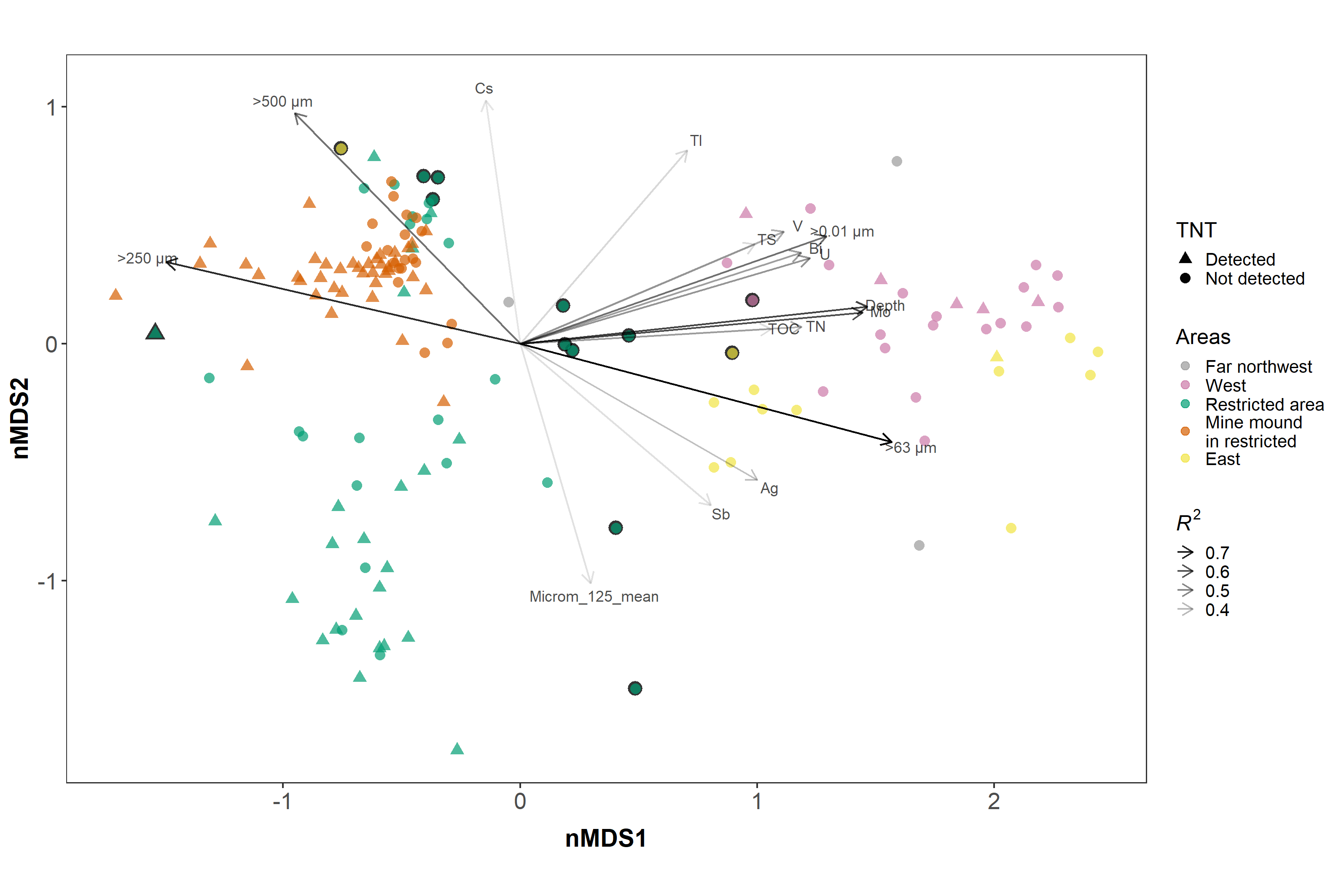


NMDS ordination based on the abundance of the most important 25 genera. Dissimilarity between samples was calculated using the proximity matrix of an unsupervised random forest. A) The microbial communities were colored by sample area and shaped to indicate the presence of TNT. The length and shade of correlating sediment parameters (p < 0.001, R^2^ > 0.3) represents the goodness of fit. The black outline marks East (yellow) and West (purple) samples which were not multicorer samples. Similarly, the outline marks Restricted Area samples that were not part of a transect.

nMDS (stress 0.11) shows a similar separation by area with an overlap of Mine mound and Restricted area samples. These areas were separated from East and West samples by the grain size fractions >250 µm. Most environmental factors correlate with the finer sediment fractions of the multicorer samples and no munition compounds were found to be significant. PCA showed weak correlation of Cobalt and 1000 µm grain size fraction, which was not true for nMDS. In conclusion, the nMDS ordination results displays the similarities of the community compositions similarly to the proximity matrix-based PCA ordination.
