# Supplementary material for: Machine Learning Predicts the Presence of 2,4,6-Trinitrotoluene in Sediments of a Baltic Sea Munitions Dumpsite Using Microbial Community Compositions
Source: Front Microbiol. 2021 Sep 29;12:626048. doi: 10.3389/fmicb.2021.626048 (PMC8513674; doi:10.3389/fmicb.2021.626048)
Supplement: Supplementary file 1 [file Data_Sheet_1.zip › Supplements_update_09_28/Supplementary_Figure_06_unsupervised_PCA_sediment_area_noline.docx]

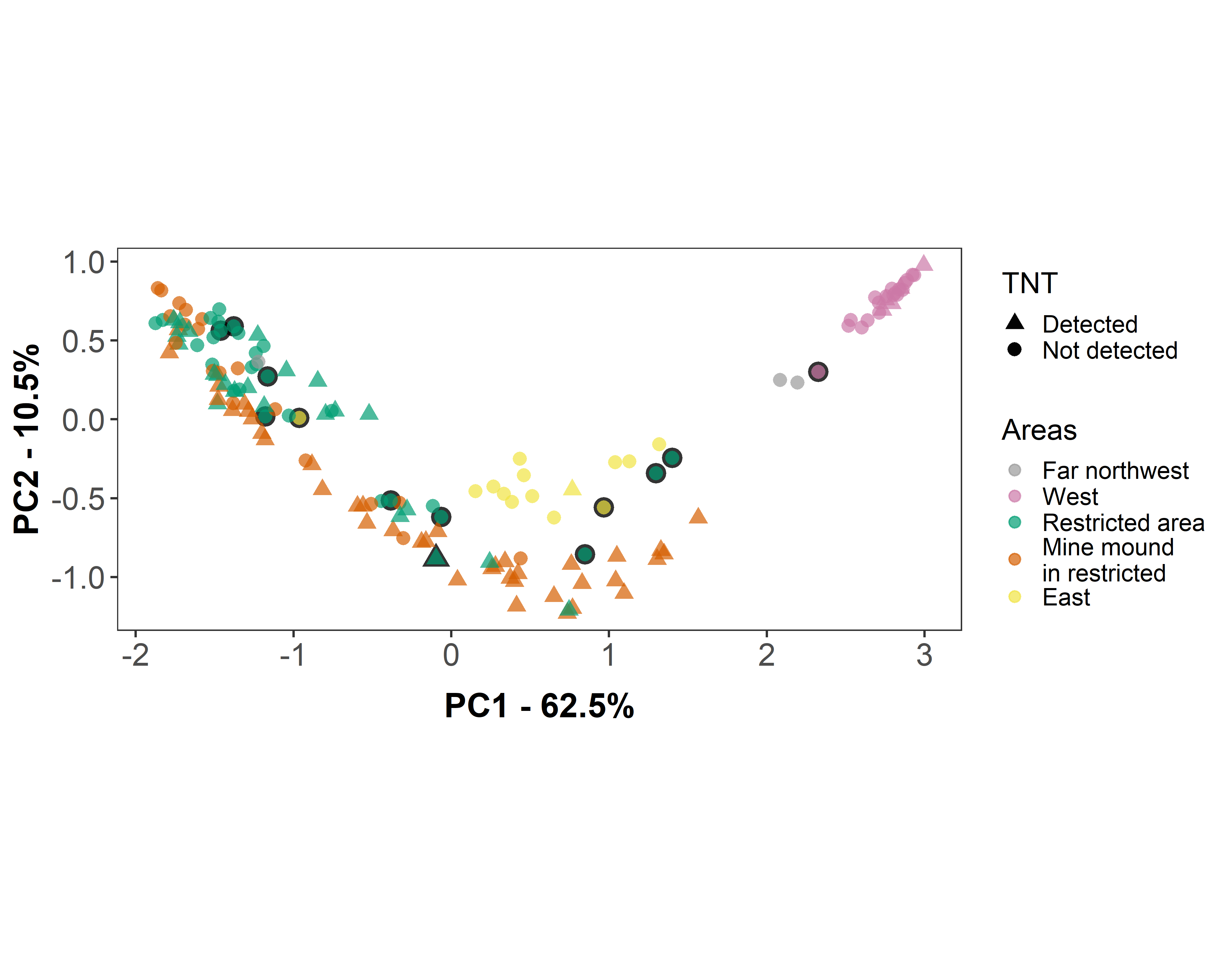


Supplementary Figure 6: PCA ordination for sediment data. The proximity matrix was generated by an unsupervised random forest classifying Full sediment data. In comparison to the PCA ordination based on the Top25 community, the core samples (West and East without black outline) were well separated herein. Furthermore, samples from the mine mound and the overall restricted area are more similar based on sediment parameters. PC1 explained 62.5% variation, which likely correlated mostly with grain size fractions, the coarser directed to the left and the finer towards the right. It is shown that samples with and without TNT were well intermixed, which might be a reason for the lower classification scores achieved by sediment data.
