# Supplementary material for: Machine Learning Predicts the Presence of 2,4,6-Trinitrotoluene in Sediments of a Baltic Sea Munitions Dumpsite Using Microbial Community Compositions
Source: Front Microbiol. 2021 Sep 29;12:626048. doi: 10.3389/fmicb.2021.626048 (PMC8513674; doi:10.3389/fmicb.2021.626048)
Supplement: Supplementary file 1 [file Data_Sheet_1.zip › Supplements_update_09_28/Supplementary_Figure_07_Sediment_spearman_rank_correlation_noline.docx]

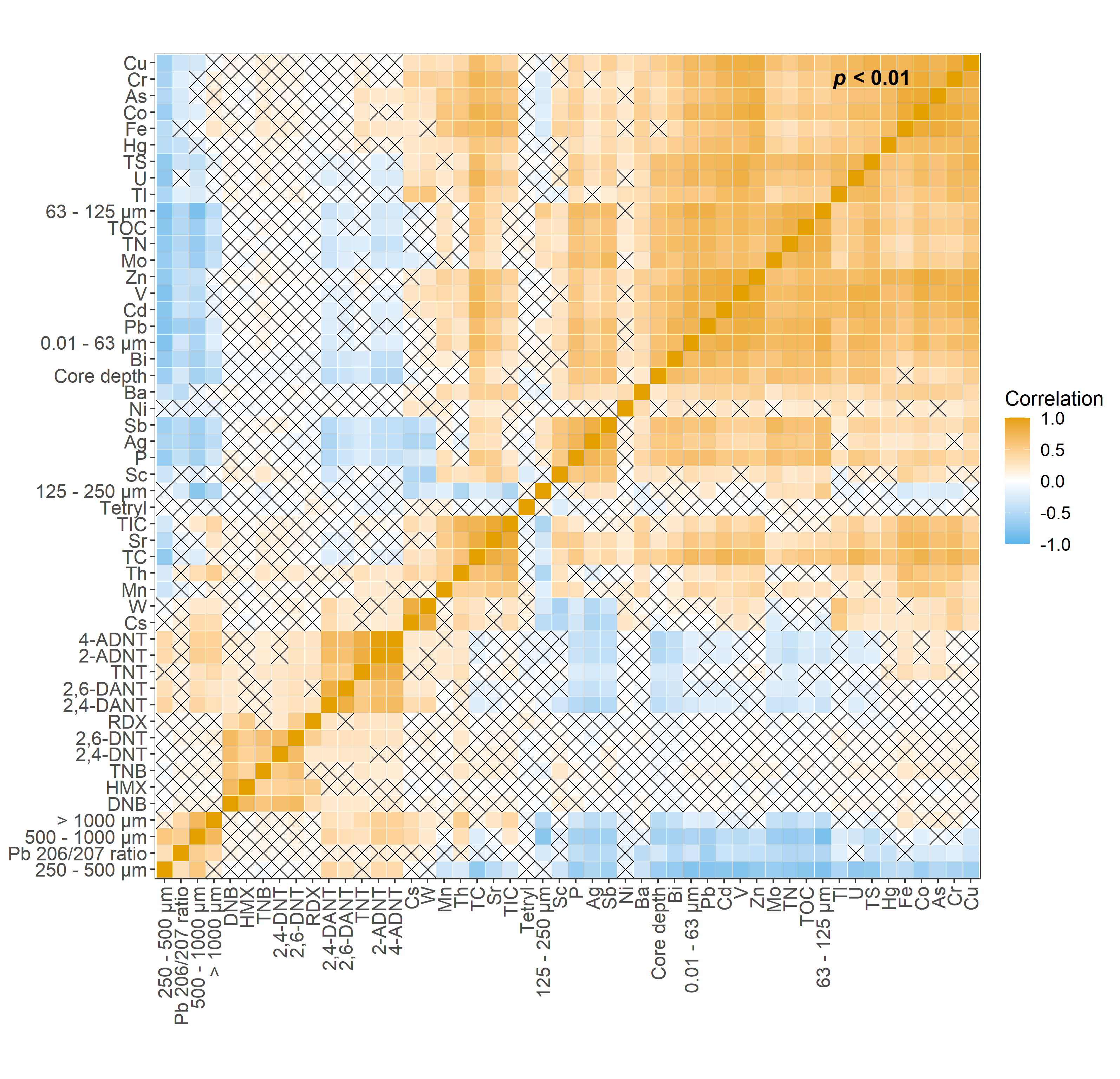


Supplementary Figure 7: Spearman rank correlation of sediment parameters. Correlations of *p* > 0.01 were signed as insignificant with an X. This analysis was performed to investigate which sediment parameters could be useful to predict TNT due to correlations. Positive correlations were found between TNT and its metabolites such as ADNTs and DANTs as well as iron, thallium, manganese, arsenic and cobalt. Furthermore, the grain size fractions 500 – 1000 µm and > 1000 µm, distinctive of the predominantly TNT-present mine mound samples, were identified. TNT was also negatively correlated with total nitrogen, antimony, silver, phosphorus, bismuth and sediment depth, hinting at the mostly TNT-absent multicorer samples. Arsenic, cobalt, total nitrogen and grain sizes were important variables for the random forest model. In further leading investigations, lead, the lead isotope ratio ^206^Pb/^207^Pb and mercury (proposed to leak from UXO) were not found to correlate with any munition compound except for a weak negative tie between 2,4-DANT and lead. Two groups of munition compounds were discernable: TNT and its metabolites and secondly, DNTs, TNB, HMX and DNB. RDX was loosely connected to both groups and Tetryl showed no correlation to any other munition compound. These results suggested that predicting TNT using other sediment parameter than its metabolites’ concentrations would turn out challenging.
