# Supplementary material for: Machine Learning Predicts the Presence of 2,4,6-Trinitrotoluene in Sediments of a Baltic Sea Munitions Dumpsite Using Microbial Community Compositions
Source: Front Microbiol. 2021 Sep 29;12:626048. doi: 10.3389/fmicb.2021.626048 (PMC8513674; doi:10.3389/fmicb.2021.626048)

Supplementary Figures 9: The following plots display the relative abundances of the 25 most important genera for TNT classification. The samples were arranged using a proximity matrix of a TNT-classifying random forest model. Each plot is identifiable via a letter in the top left, additionally the full lineage of each genus was provided. For additional context, these plots can be compared with the supervised PCA ordination colored by sampling area.

The samples were labelled with two values. The upper value indicates the amount of times this sample was wrongly predicted by the prediction robustness analysis. The lower number displays together with the color gradient the relative abundance, yellow being the highest value. The values were expressed as a percentage and rounded. Only samples containing a given genus were labelled.

Please note that it is possible to zoom into the pdf-files without loss of resolution.

A

Bacteria_Cyanobacteria_Sericytochromatia_NA_NA_NA

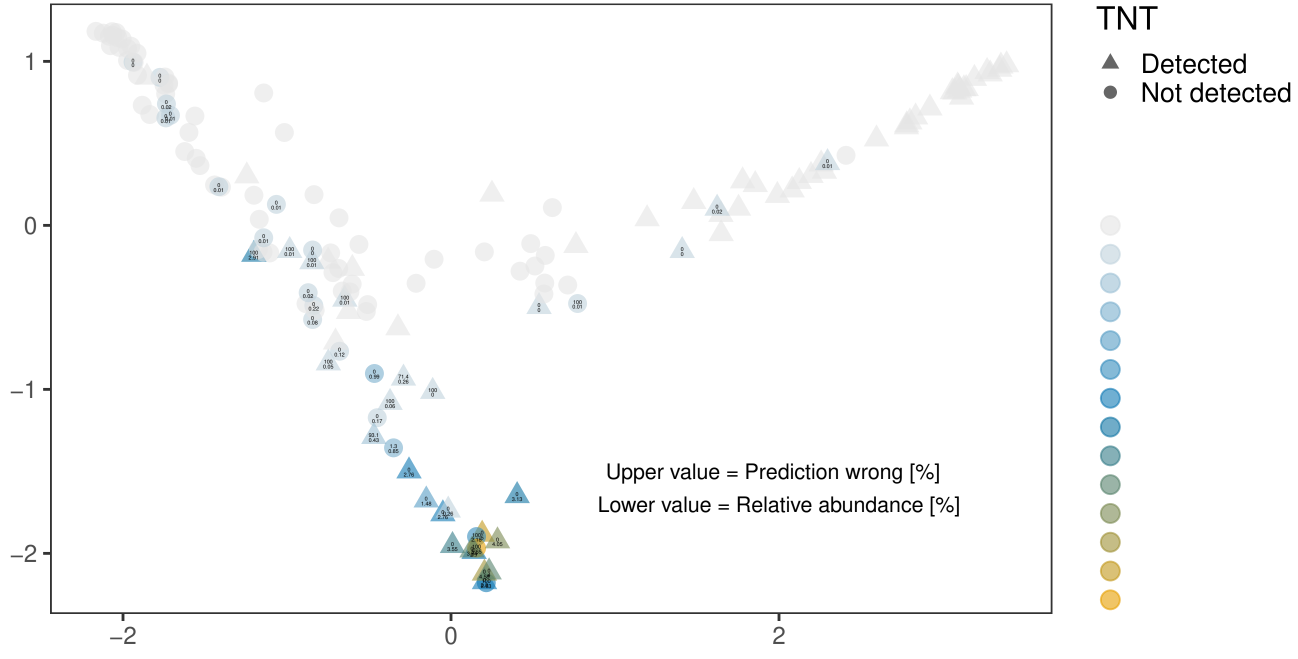

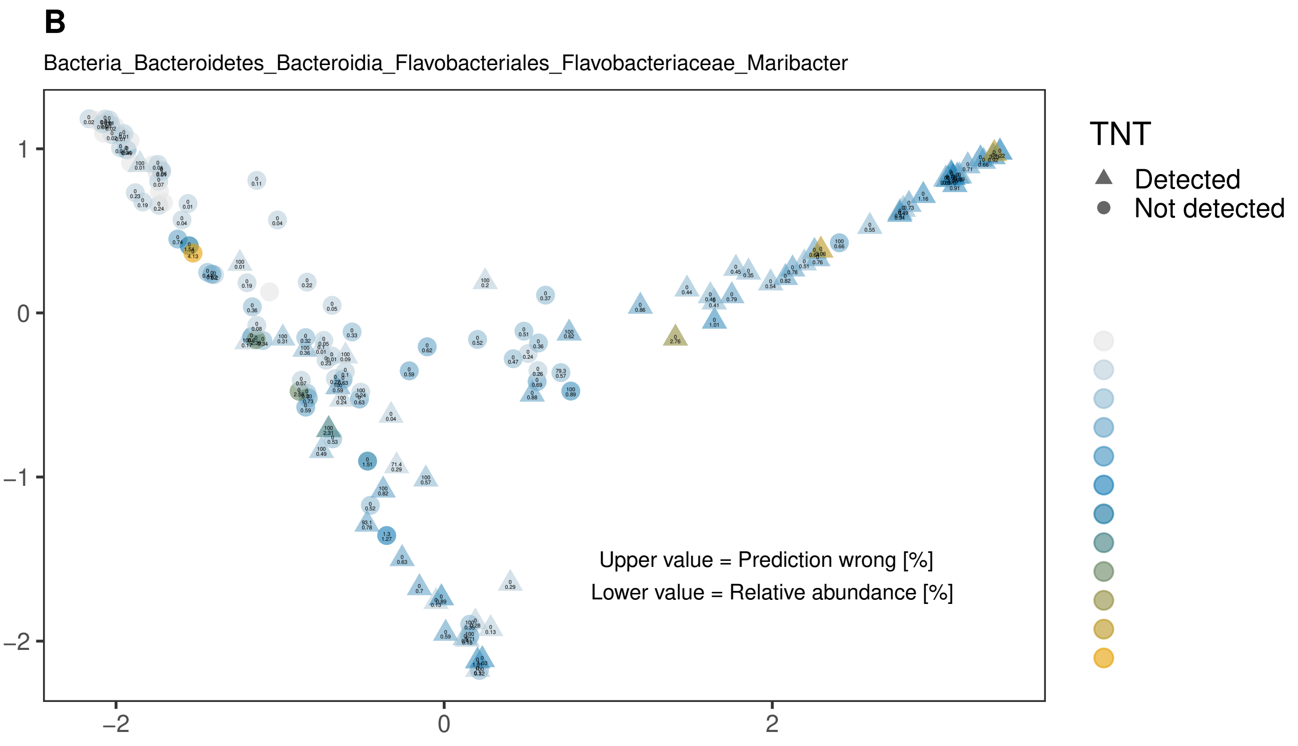
B
Bacteria_Bacteroidetes_Bacteroidia_Flavobacteriales_Flavobacteriaceae_Maribacter
TNT
Detected
Not detected
Upper value = Prediction wrong [%]
Lower value = Relative abundance [%]
1
0
−1
−2
−2
0
2

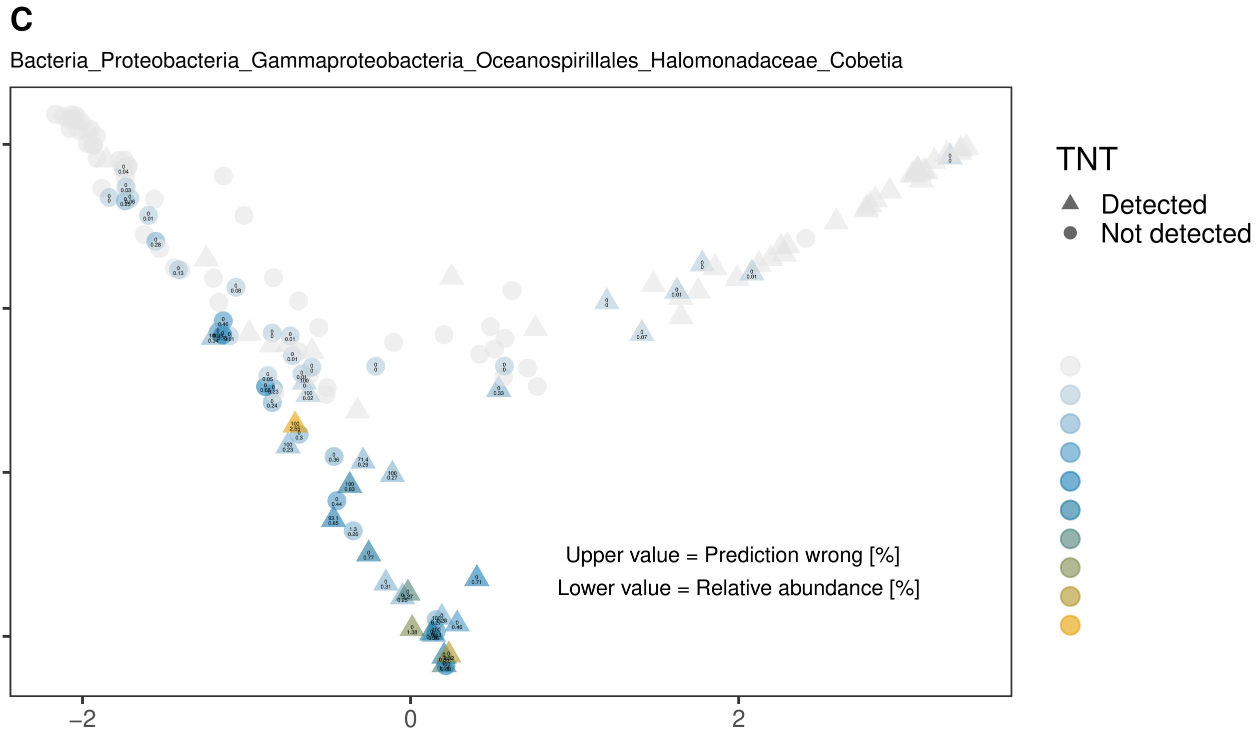
C
Bacteria_Proteobacteria_Gammaproteobacteria_Oceanospirillales_Halomonadaceae_Cobetia
TNT
Detected
Not detected
Upper value = Prediction wrong [%]
Lower value = Relative abundance [%]
1
0
−1
−2
−2
0
2

D

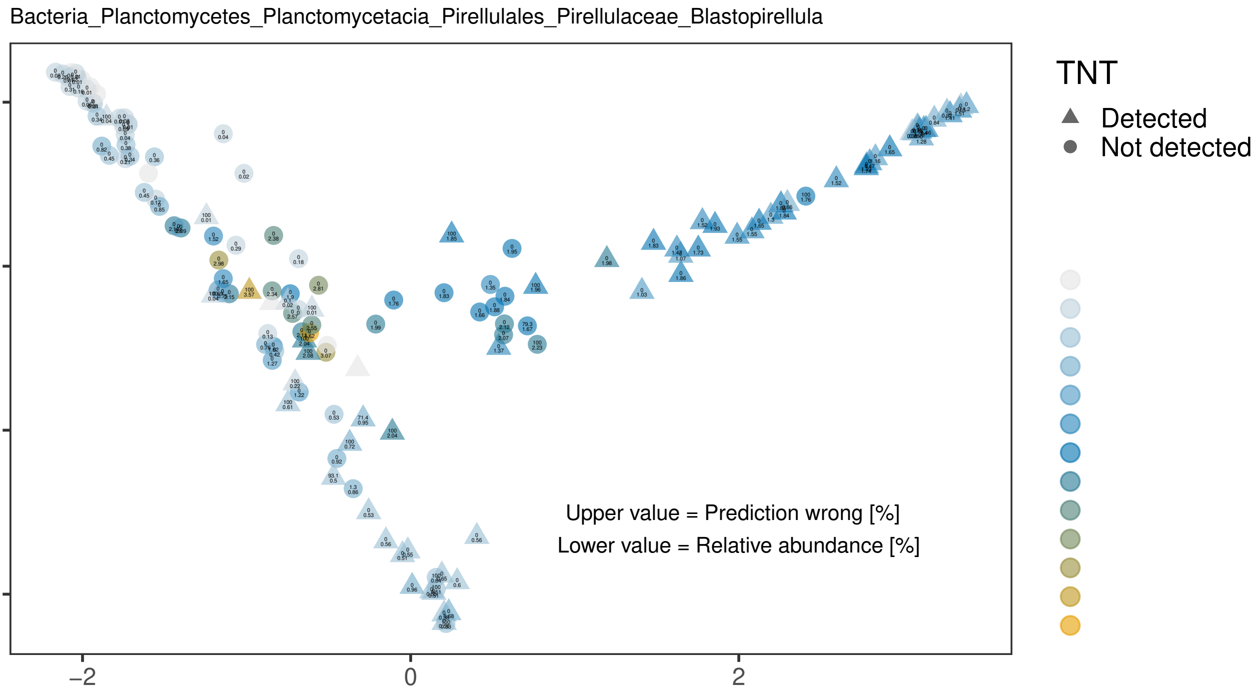

Bacteria_Planctomycetes_Planctomycetacia_Pirellulales_Pirellulaceae_Blastopirellula
TNT
Detected
Not detected
Upper value = Prediction wrong [%]
Lower value = Relative abundance [%]
1
0
−1
−2
−2
0
2

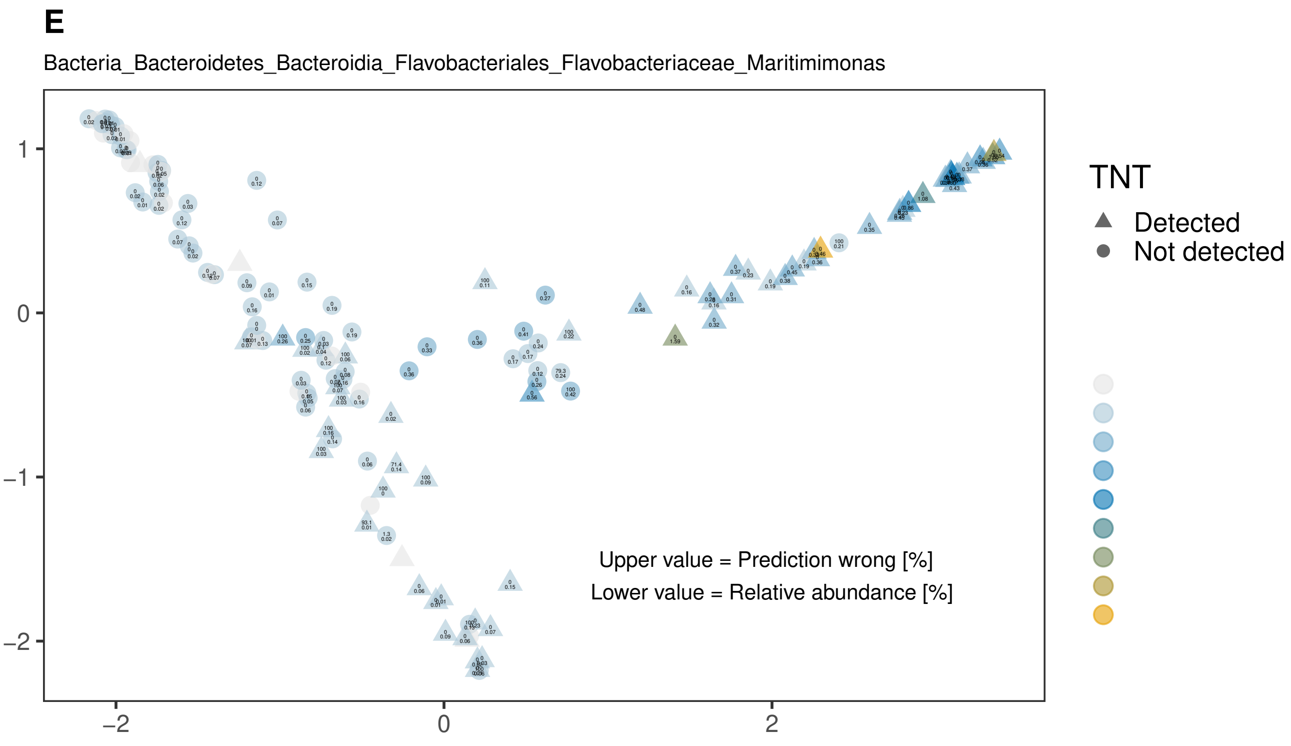
E
Bacteria_Bacteroidetes_Bacteroidia_Flavobacteriales_Flavobacteriaceae_Maritimimonas
1
0
−1
−2
−2
0
2
Upper value = Prediction wrong [%]
Lower value = Relative abundance [%]
TNT
Detected
Not detected

F

Bacteria_Proteobacteria_Gammaproteobacteria_Oceanospirillales_Nitrincolaceae_Motiliproteus

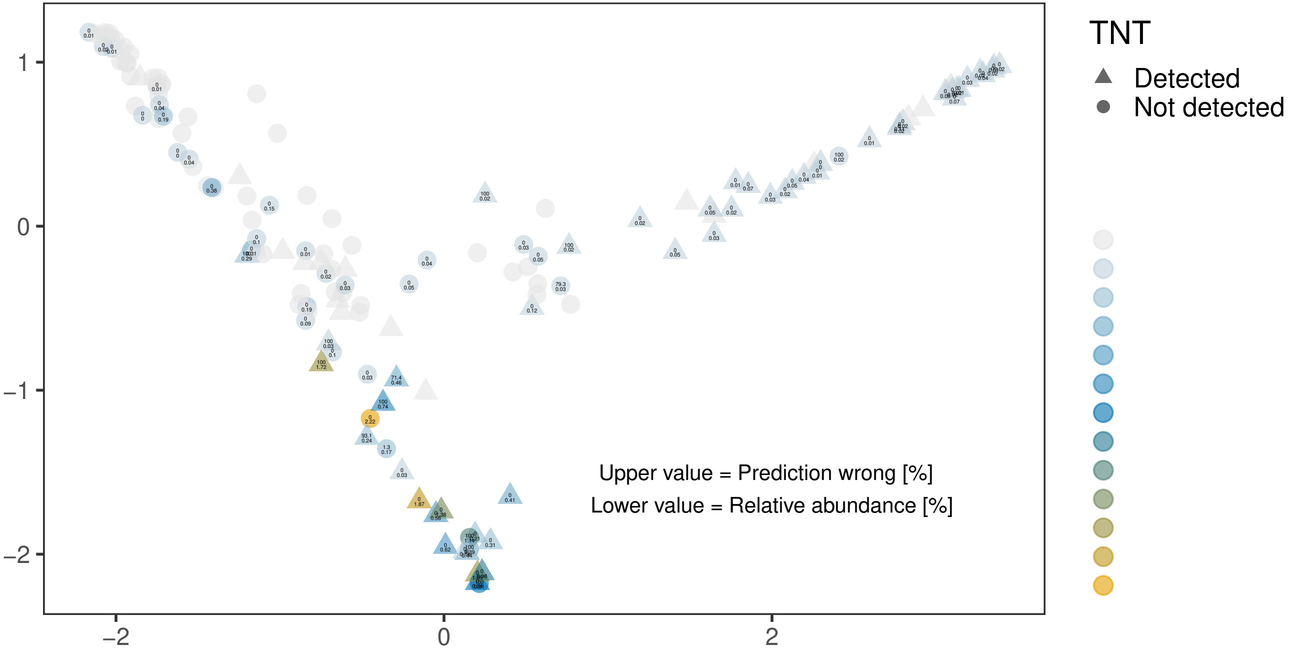

G

Bacteria_Proteobacteria_Gammaproteobacteria_Alteromonadales_Colwelliaceae_NA

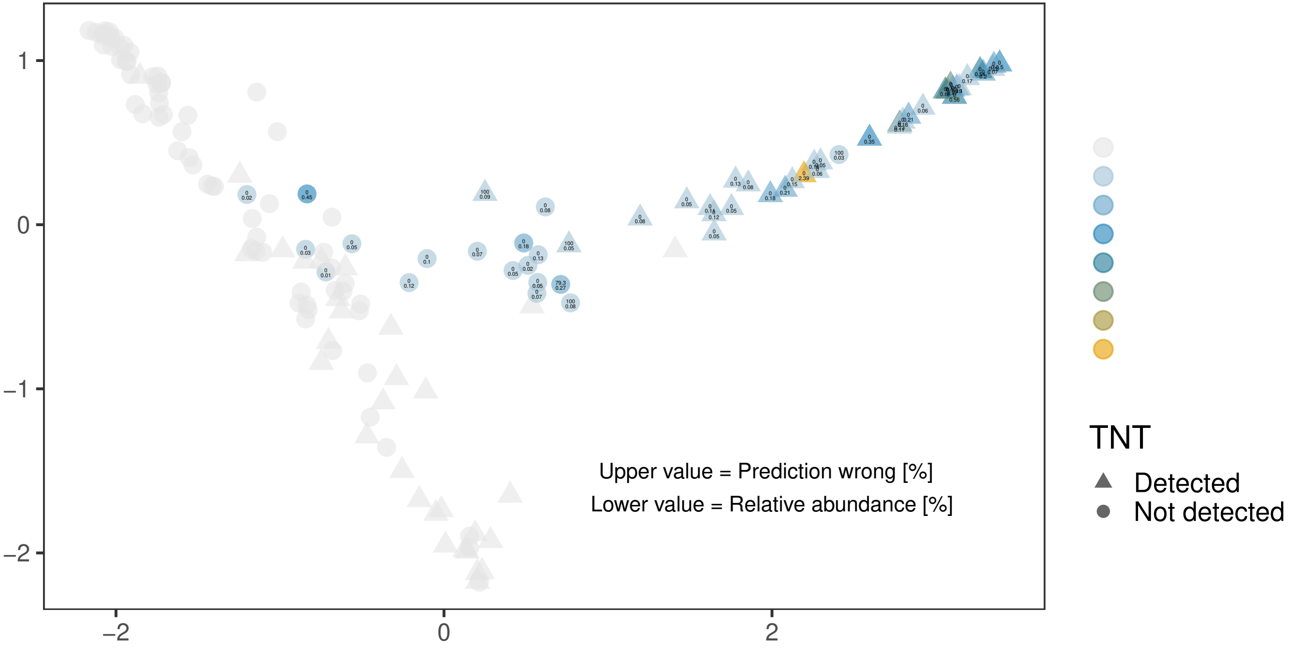

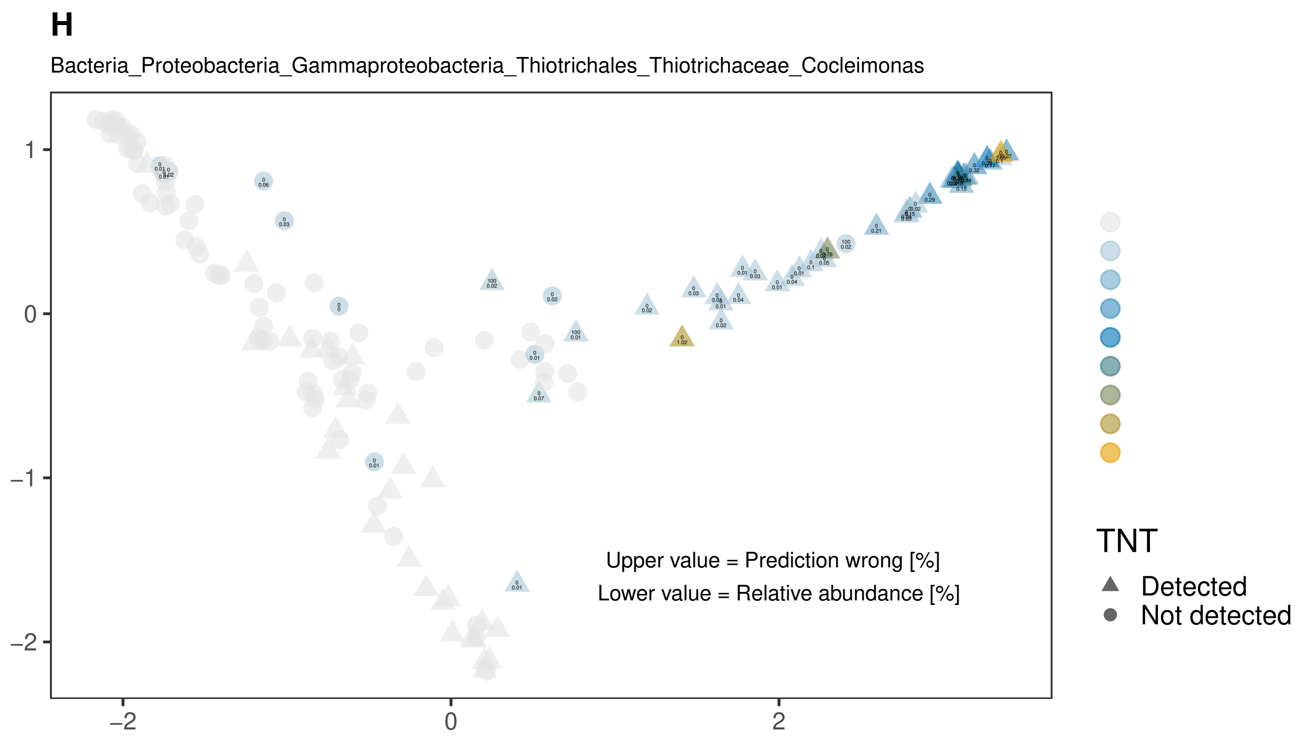
H
Bacteria_Proteobacteria_Gammaproteobacteria_Thiotrichales_Thiotrichaceae_Cocleimonas
Upper value = Prediction wrong [%]
Lower value = Relative abundance [%]
TNT
Detected
Not detected
1
0
−1
−2
−2
0
2

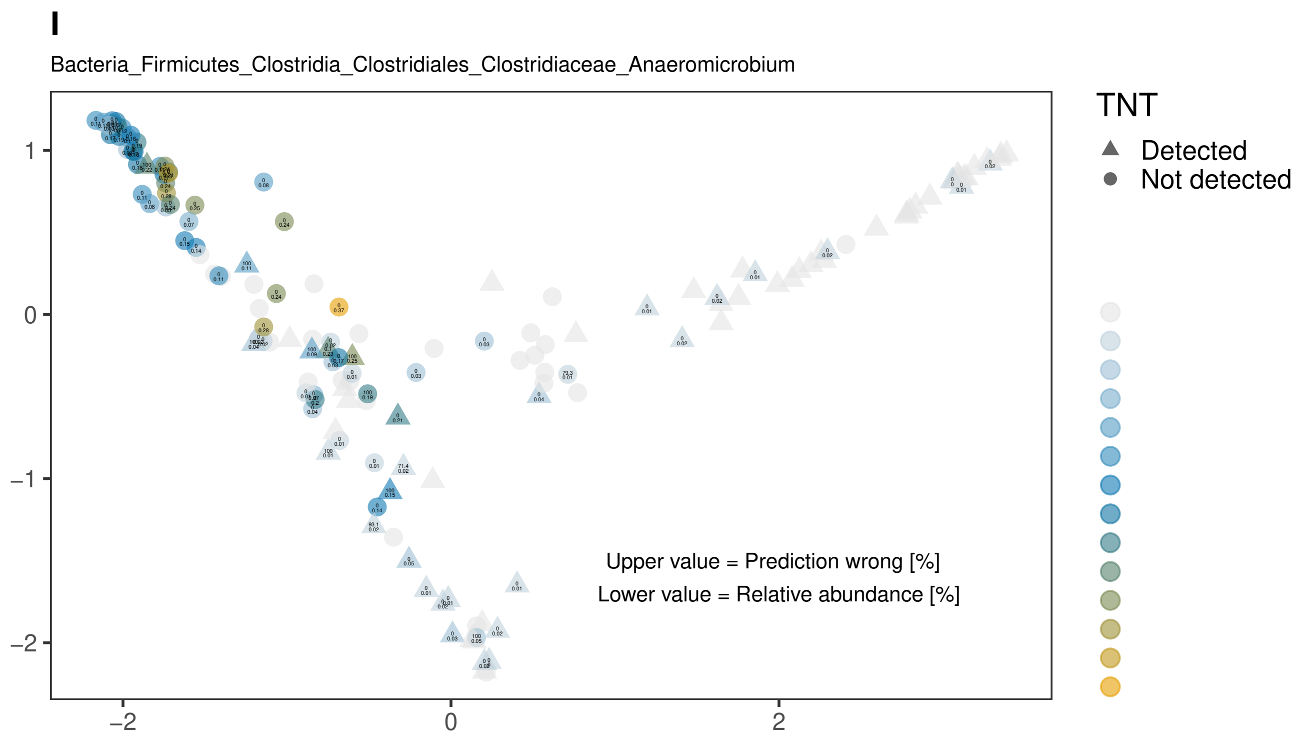
I
Bacteria_Firmicutes_Clostridia_Clostridiales_Clostridiaceae_Anaeromicrobium
TNT
Detected
Not detected
Upper value = Prediction wrong [%]
Lower value = Relative abundance [%]
1
0
−1
−2
−2
0
2

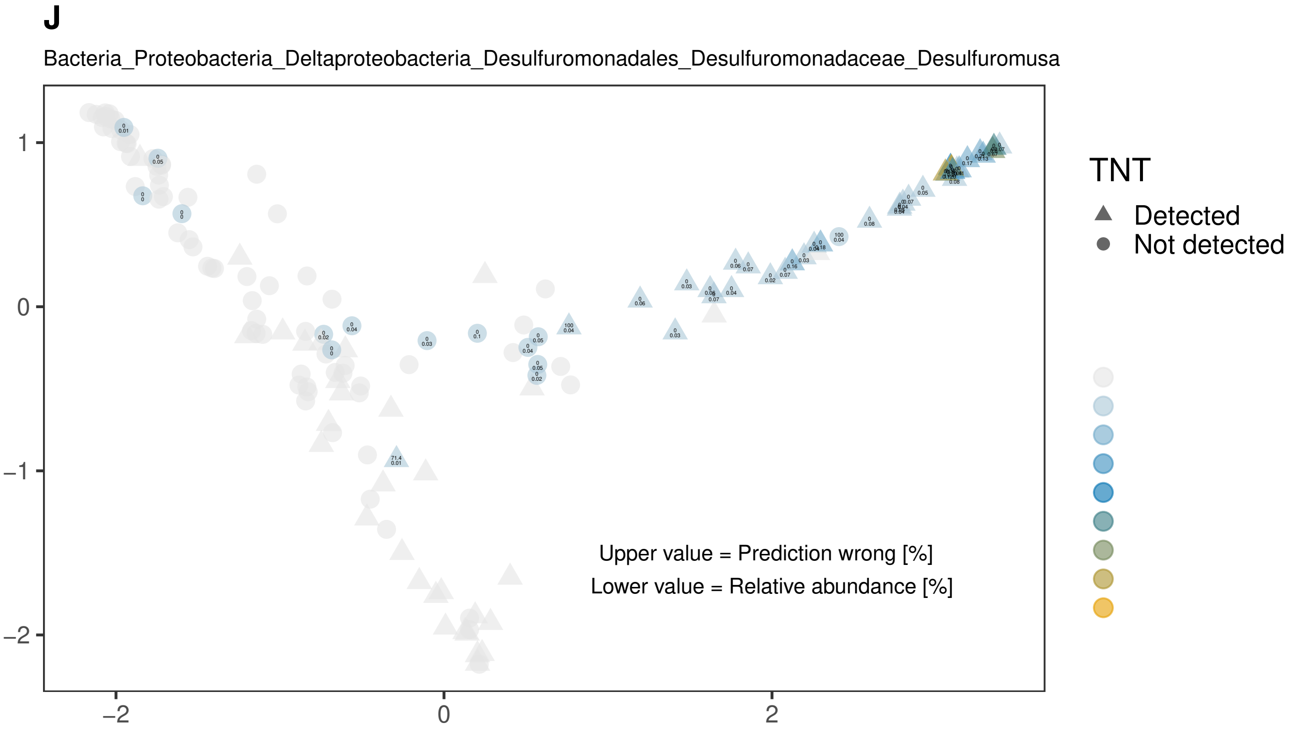
J
Bacteria_Proteobacteria_Deltaproteobacteria_Desulfuromonadales_Desulfuromonadaceae_Desulfuromusa
TNT
Detected
Not detected
Upper value = Prediction wrong [%]
Lower value = Relative abundance [%]

K

Bacteria_Proteobacteria_Deltaproteobacteria_Desulfobacterales_Desulfobulbaceae_MSBL7

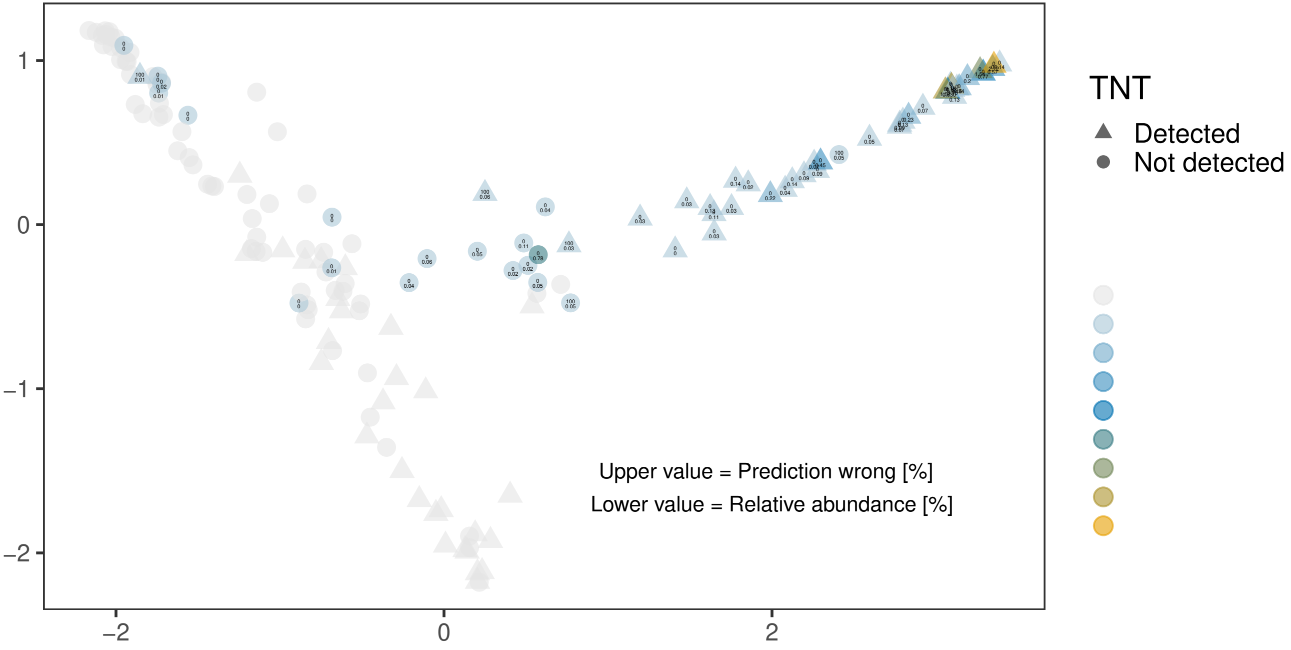

L

Bacteria_Proteobacteria_Deltaproteobacteria_SAR324_clade(Marine_group_B)_NA_NA

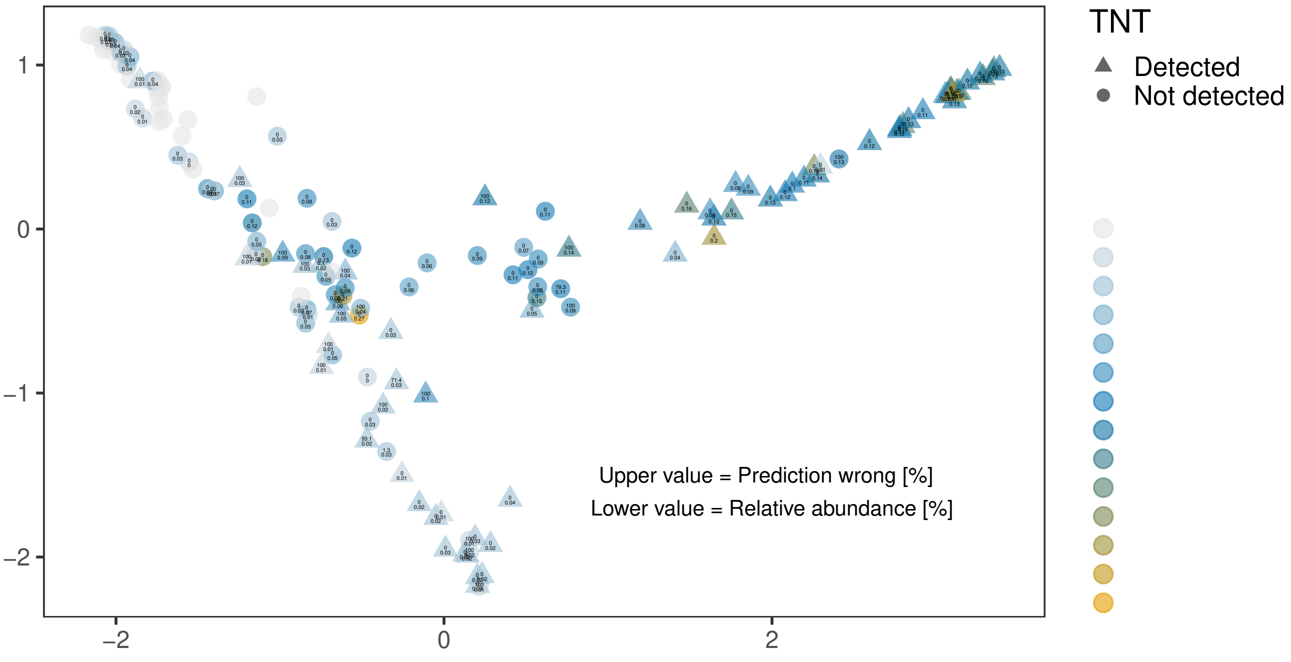

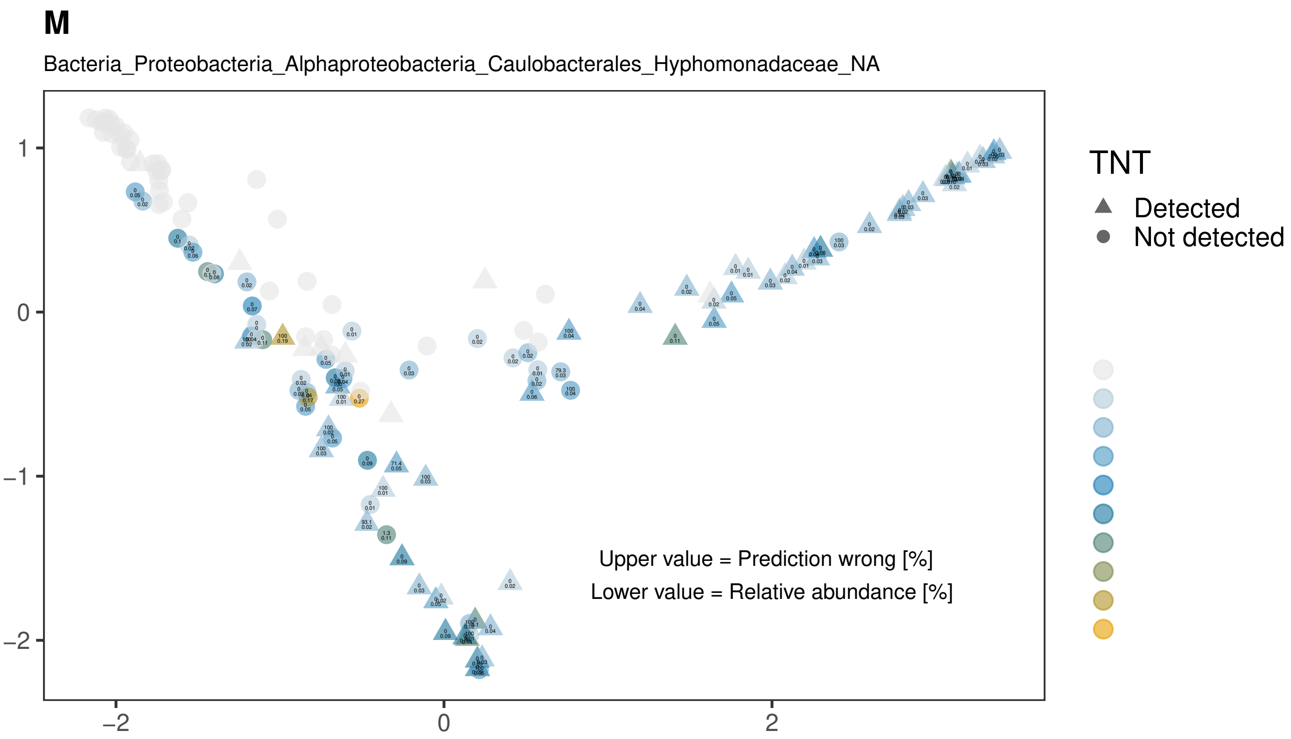
M
Bacteria_Proteobacteria_Alphaproteobacteria_Caulobacterales_Hyphomonadaceae_NA
TNT
Detected
Not detected
Upper value = Prediction wrong [%]
Lower value = Relative abundance [%]
1
0
−1
−2
−2
0
2

N

Bacteria_Proteobacteria_Deltaproteobacteria_Bdellovibrionales_Bdellovibrionaceae_OM27_clade

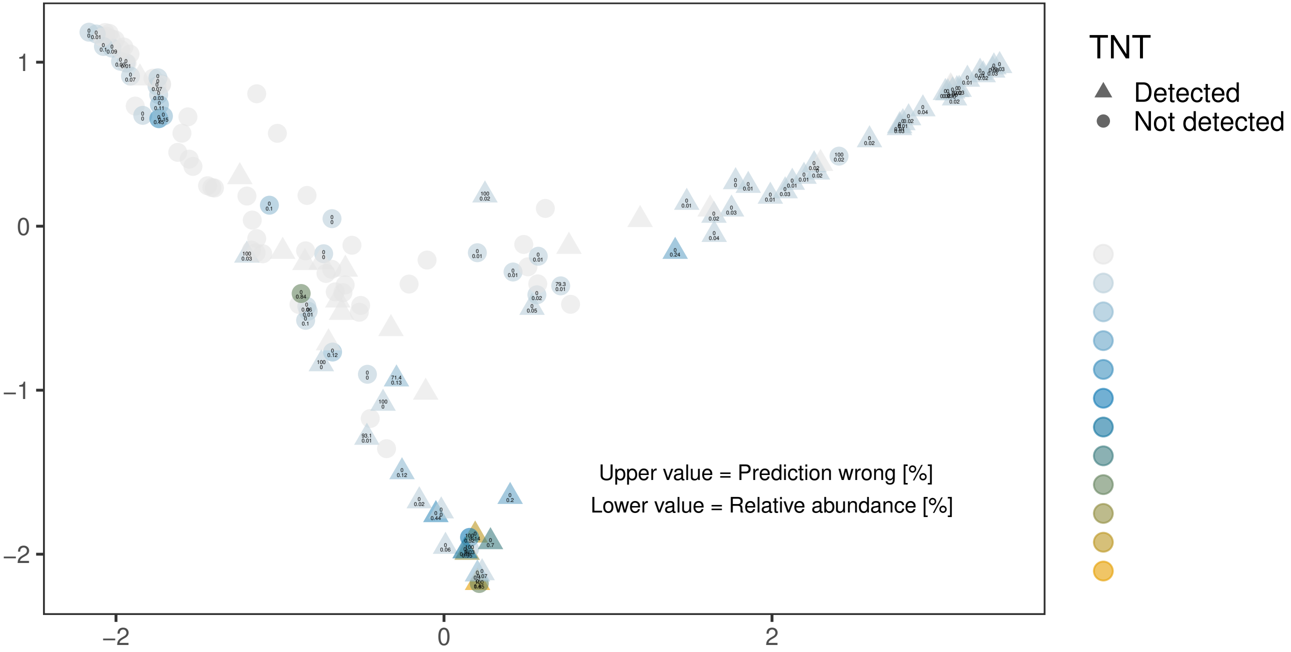

O

Bacteria_Gemmatimonadetes_Gemmatimonadetes_Gemmatimonadales_Gemmatimonadaceae_NA

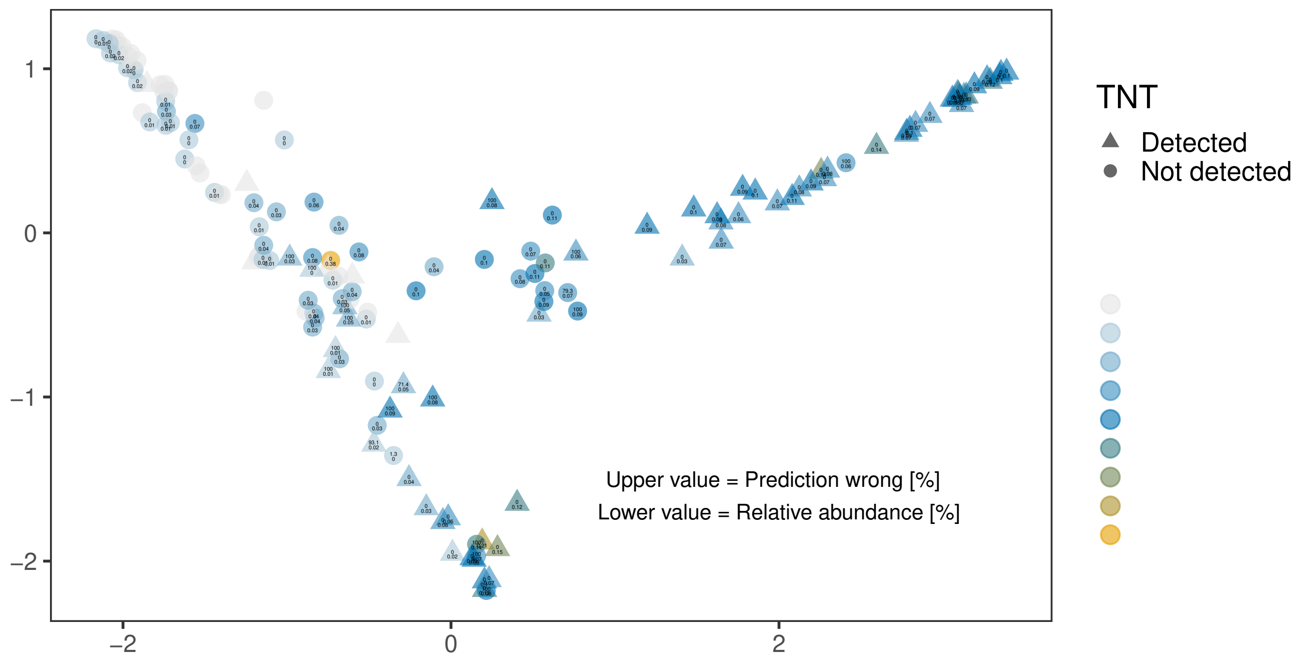

P

Bacteria_Chloroflexi_Anaerolineae_SBR1031_A4b_NA

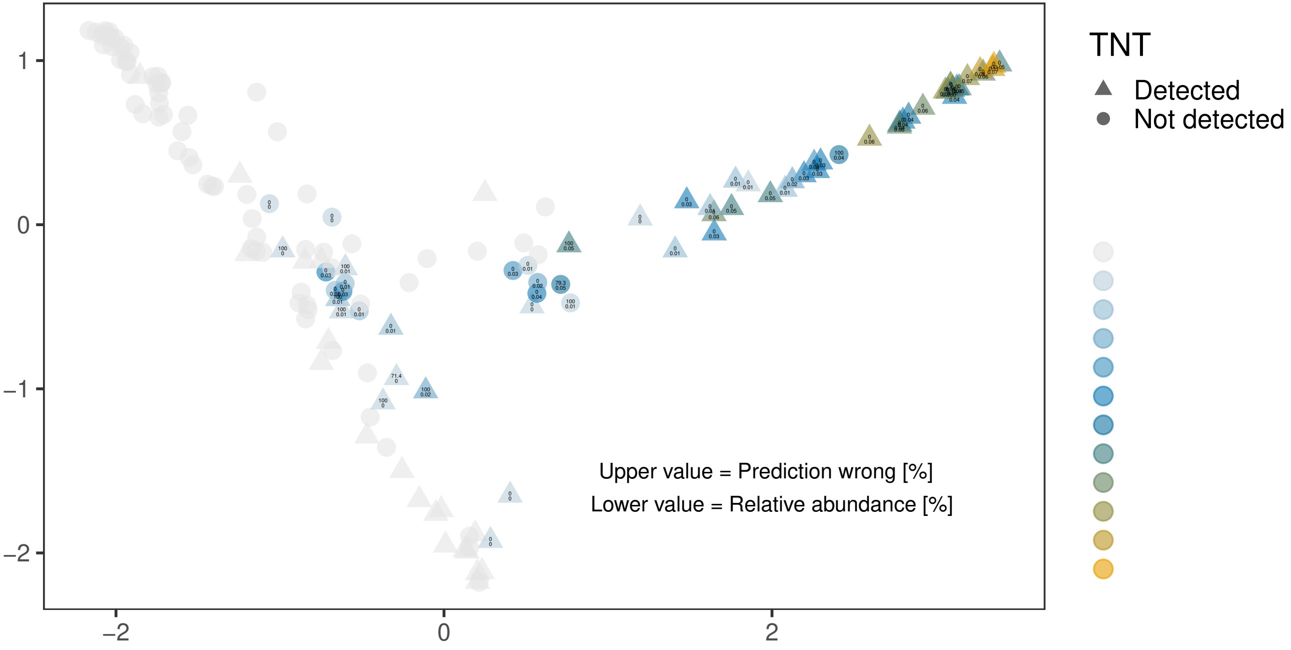

Q

Bacteria_Proteobacteria_Deltaproteobacteria_Desulfobacterales_Desulfobulbaceae_Desulfocapsa

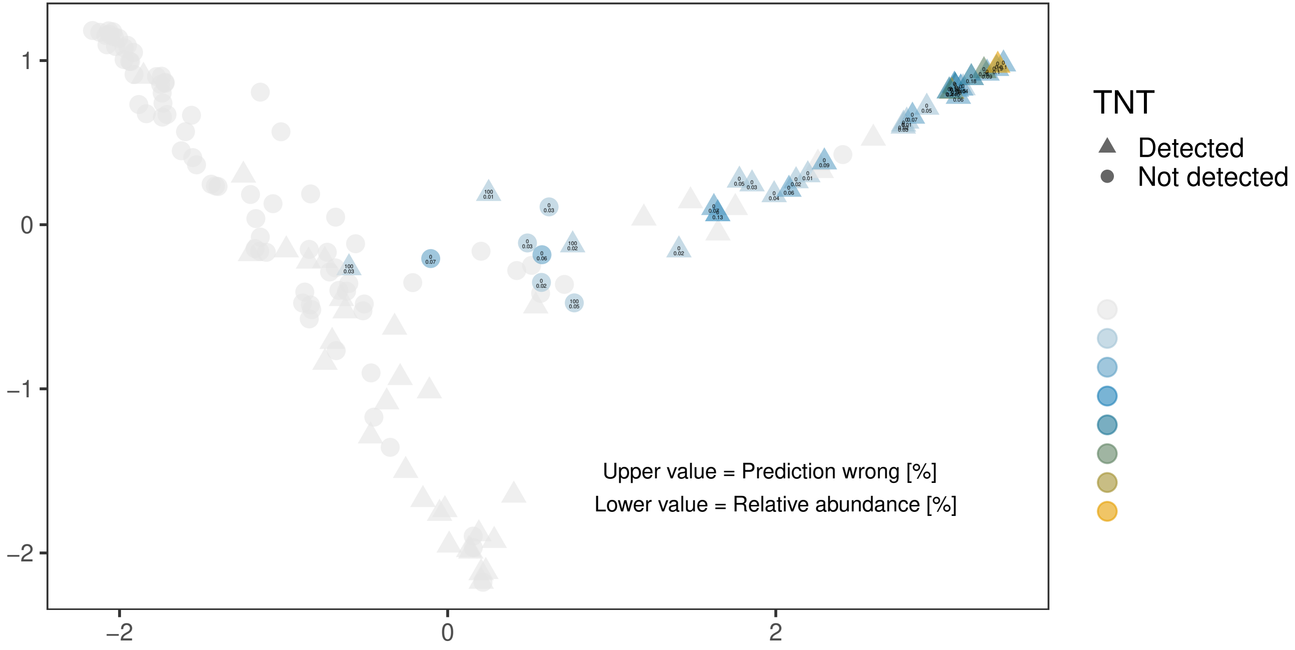

R

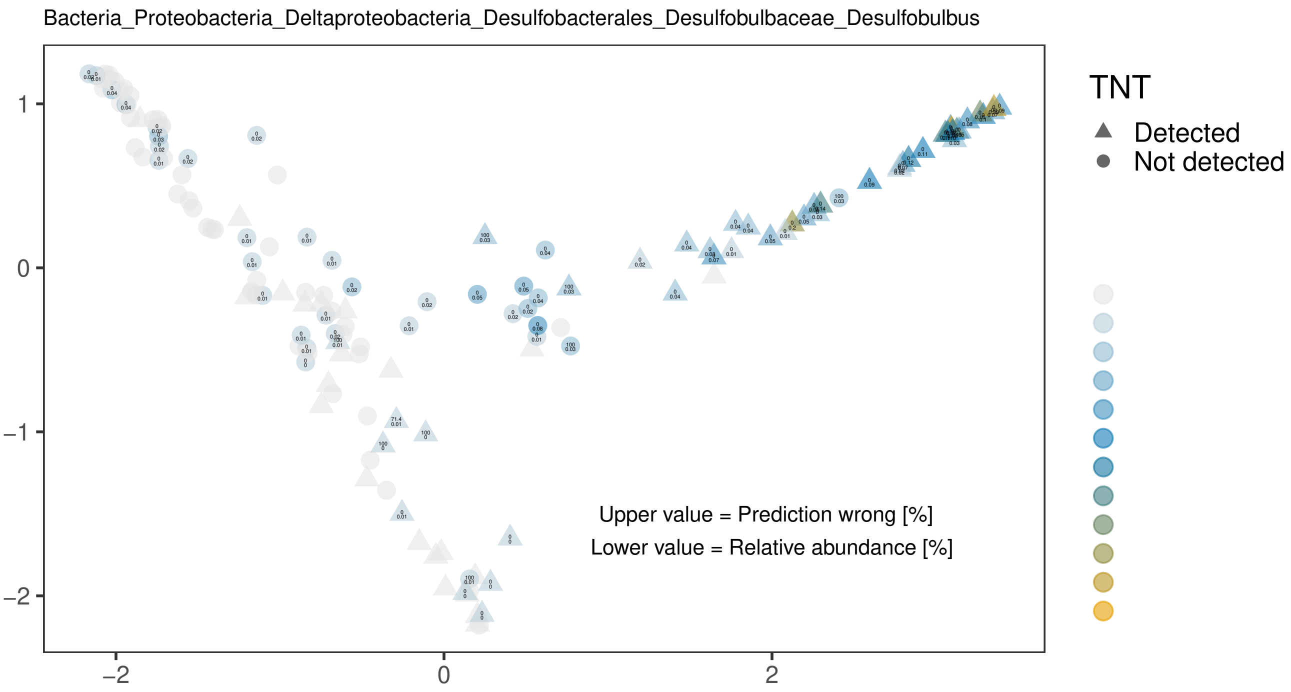

Bacteria_Proteobacteria_Deltaproteobacteria_Desulfobacterales_Desulfobulbaceae_Desulfobulbus
TNT
Detected
Not detected
Upper value = Prediction wrong [%]
Lower value = Relative abundance [%]

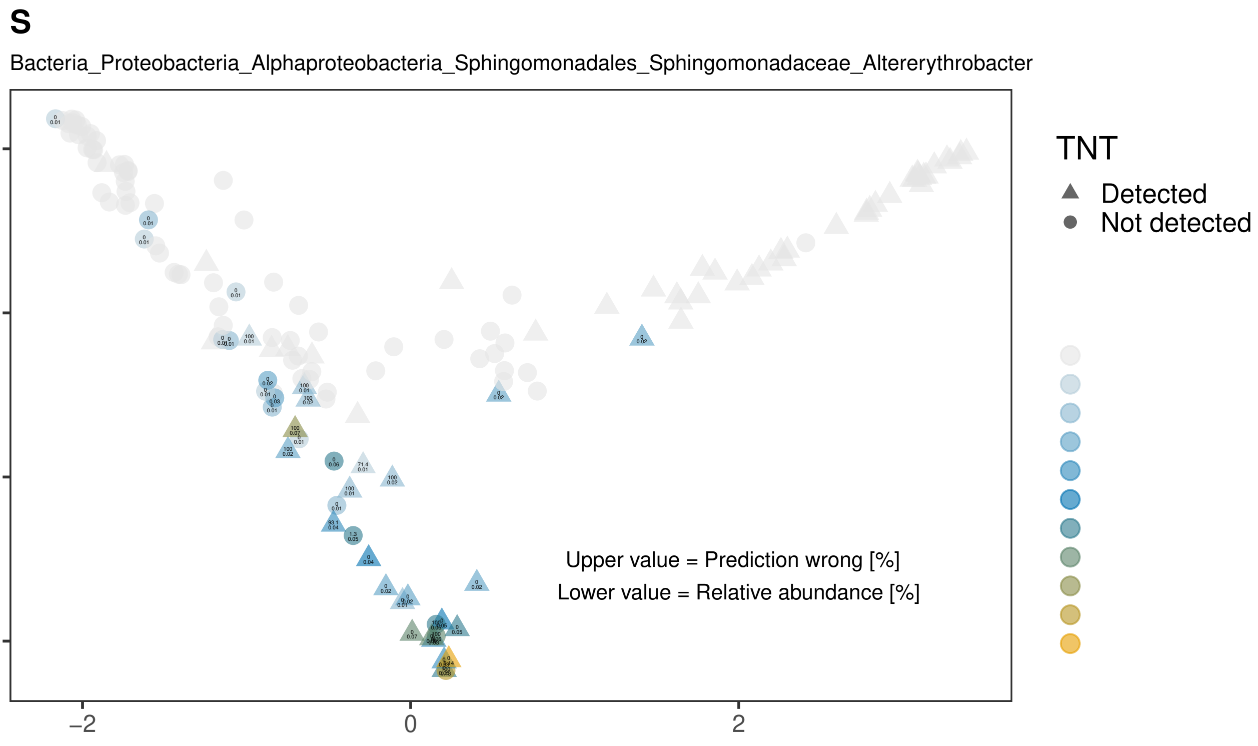
S
Bacteria_Proteobacteria_Alphaproteobacteria_Sphingomonadales_Sphingomonadaceae_Altererythrobacter
1
0
−1
−2
−2
0
2
Upper value = Prediction wrong [%]
Lower value = Relative abundance [%]
TNT
Detected
Not detected

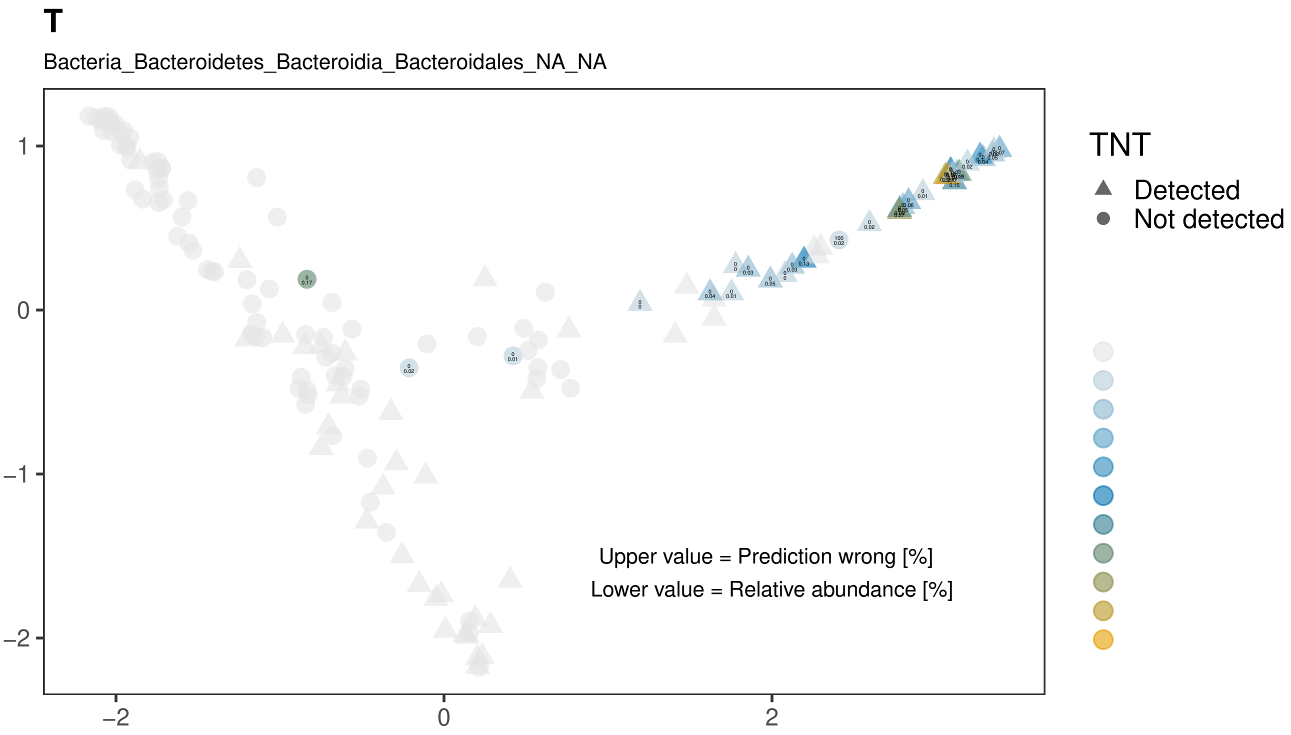
T
Bacteria_Bacteroidetes_Bacteroidia_Bacteroidales_NA_NA
TNT
Detected
Not detected
Upper value = Prediction wrong [%]
Lower value = Relative abundance [%]
1
0
−1
−2
−2
0
2

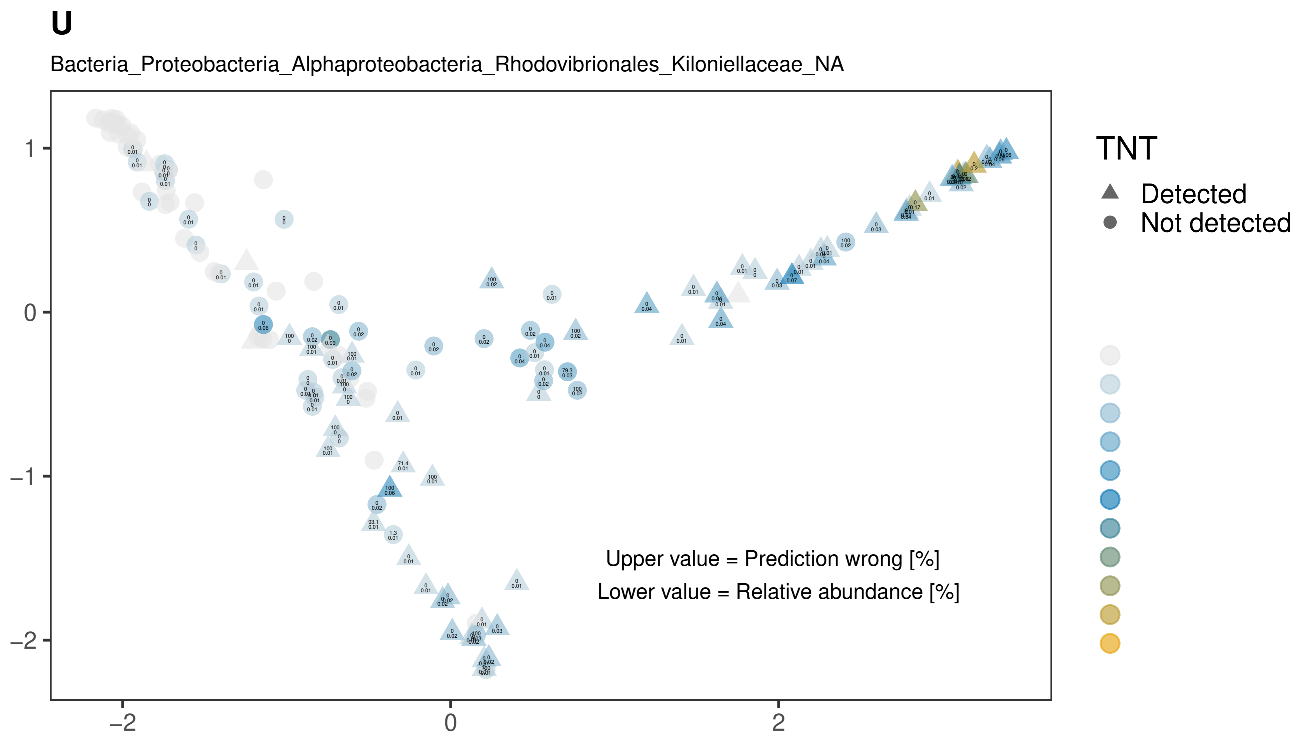
U
Bacteria_Proteobacteria_Alphaproteobacteria_Rhodovibrionales_Kiloniellaceae_NA
1
0
−1
−2
−2
0
2
Upper value = Prediction wrong [%]
Lower value = Relative abundance [%]
TNT
Detected
Not detected

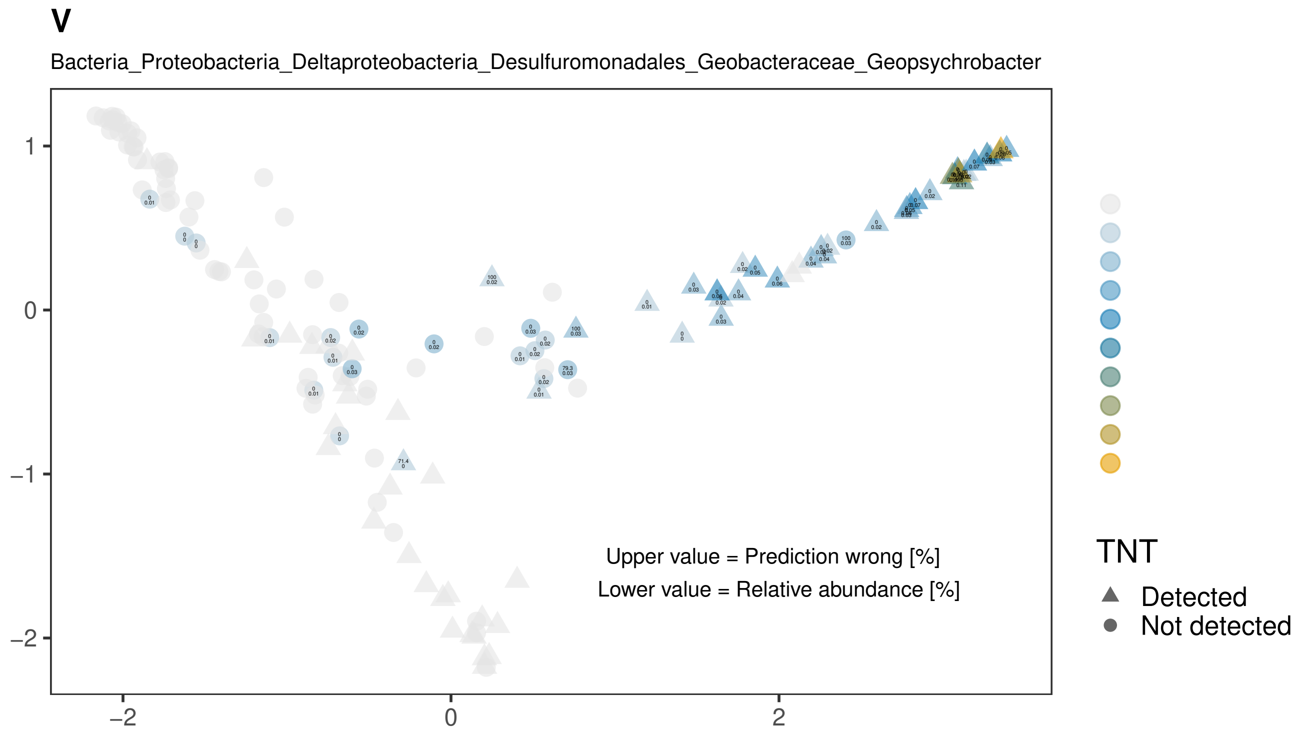
V
Bacteria_Proteobacteria_Deltaproteobacteria_Desulfuromonadales_Geobacteraceae_Geopsychrobacter
1
0
−1
−2
−2
0
2
Upper value = Prediction wrong [%]
Lower value = Relative abundance [%]
TNT
Detected
Not detected

W

Archaea_Euryarchaeota_Methanobacteria_Methanobacteriales_Methanobacteriaceae_Methanobacterium

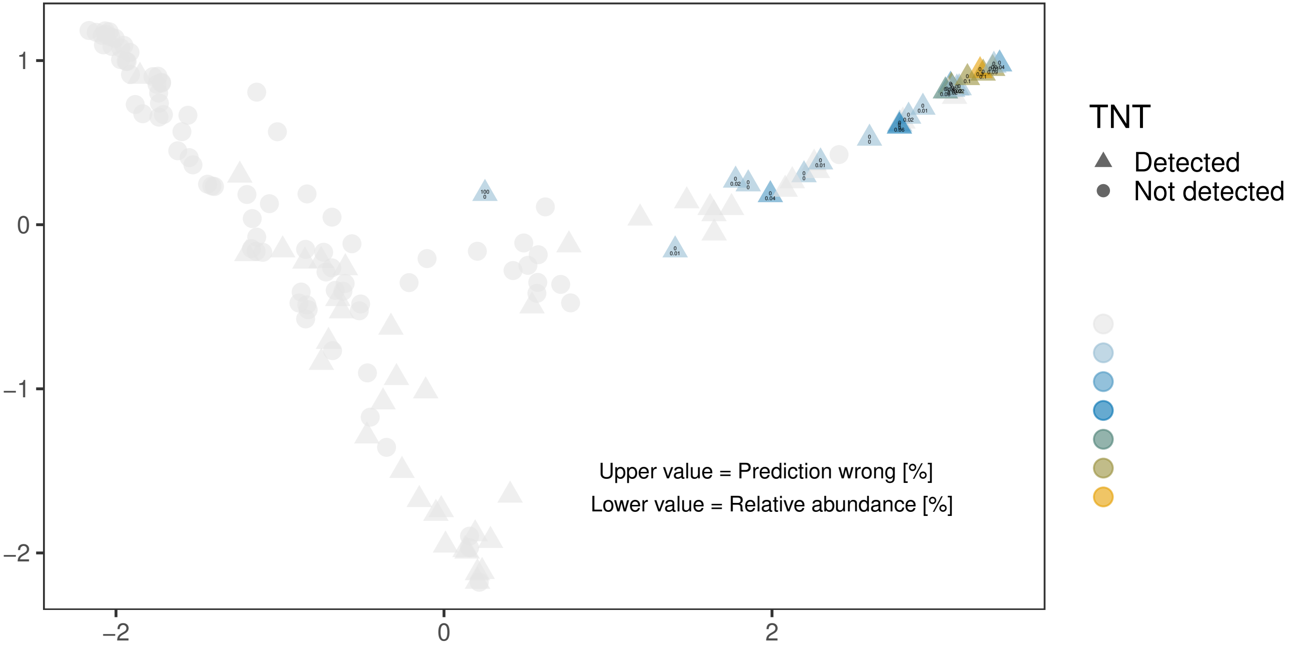

X

Bacteria_Planctomycetes_Planctomycetacia_Planctomycetales_Gimesiaceae_NA

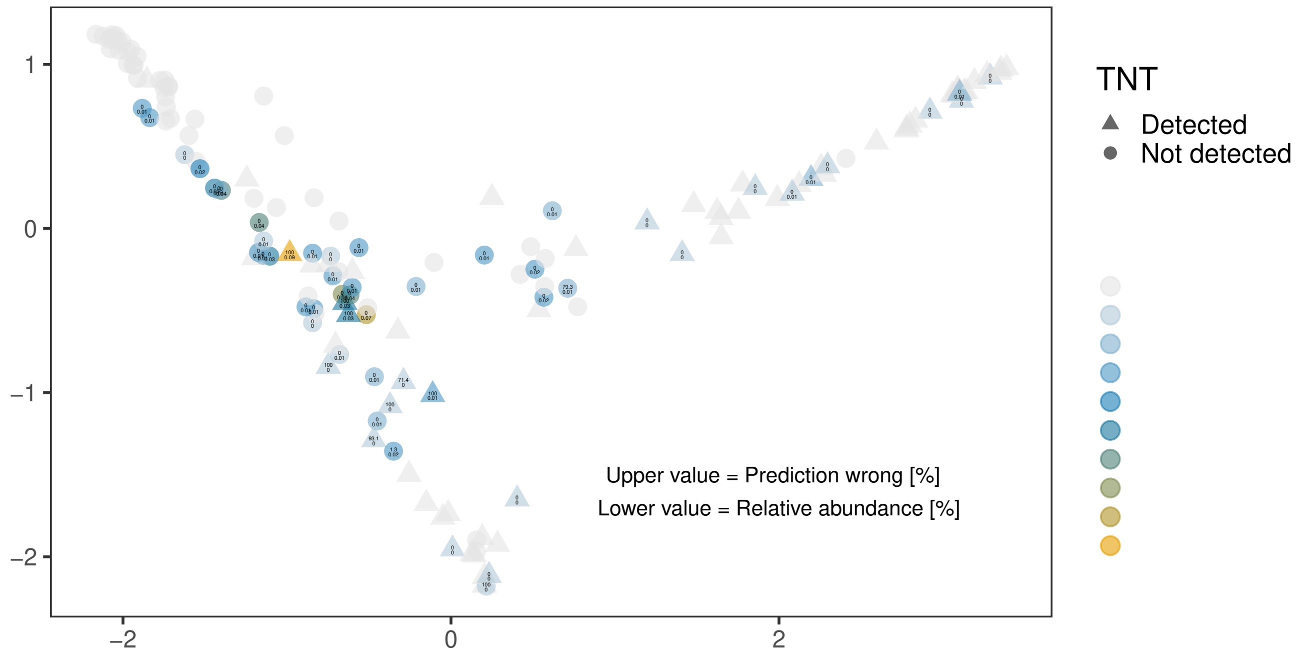

Y

Bacteria_TA06_NA_NA_NA_NA

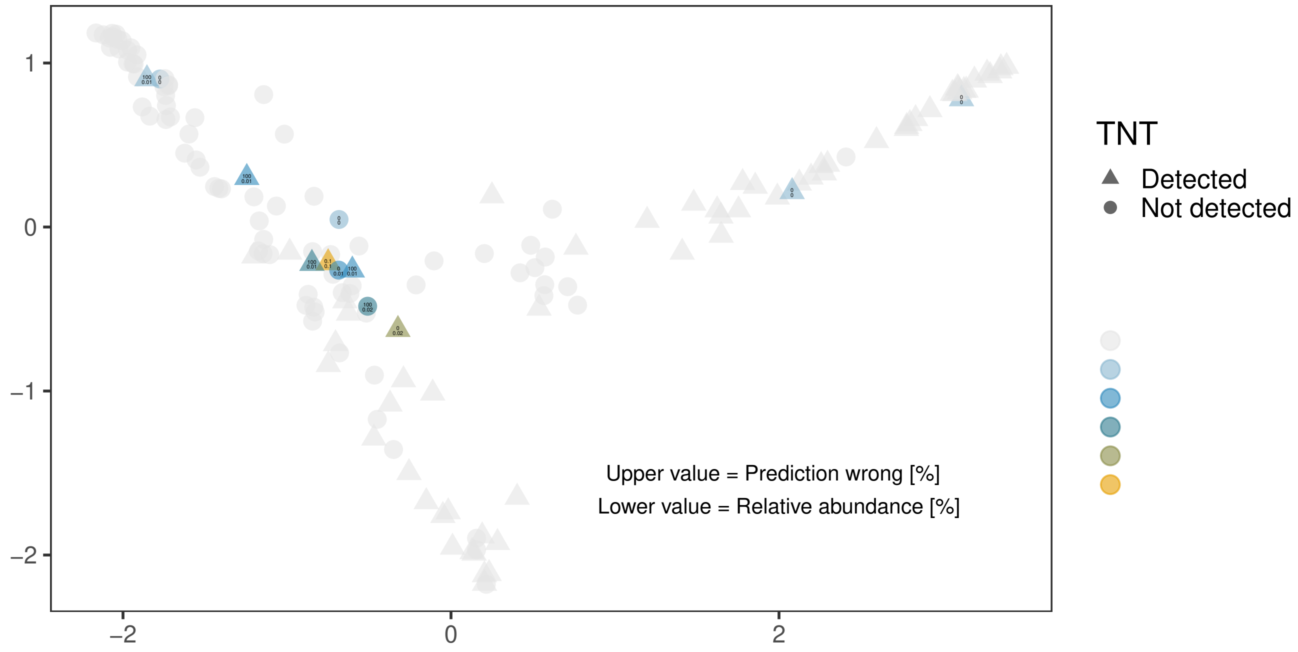

Supplement: Supplementary file 1 [file Data_Sheet_1.zip › Supplements_update_09_28/Supplementary_Figure_09_Ordinations_supervised_abundance_genus.pdf]
