# Supplementary material for: Machine Learning Predicts the Presence of 2,4,6-Trinitrotoluene in Sediments of a Baltic Sea Munitions Dumpsite Using Microbial Community Compositions
Source: Front Microbiol. 2021 Sep 29;12:626048. doi: 10.3389/fmicb.2021.626048 (PMC8513674; doi:10.3389/fmicb.2021.626048)
Supplement: Supplementary file 1 [file Data_Sheet_1.zip › Supplements_update_09_28/Supplementary_Figure_10_Ordinations_unsupervised_abundance_sediment.pdf]

Supplementary Figures 10: The following plots display the prevalence of selected sediment parameters including the nine most important for TNT classification. The samples were arranged using a proximity matrix of an unsupervised random forest model with Top25 community as input data. Each plot is identifiable via a letter in the top left, additionally the parameter name was provided. For additional context, these plots can be compared with the unsupervised PCA ordination colored by sampling area.

The samples were labelled with two values. The upper value indicates the amount of times this sample was wrongly predicted by the prediction robustness analysis, expressed as a percentage. The lower number displays together with the color gradient the fraction or concentration, yellow being the highest value. The corresponding unit is indicated behind the parameter name. Both values were rounded. Only samples where a specific parameter was found were labelled.

Please note that it is possible to zoom into the pdf-files without loss of resolution.

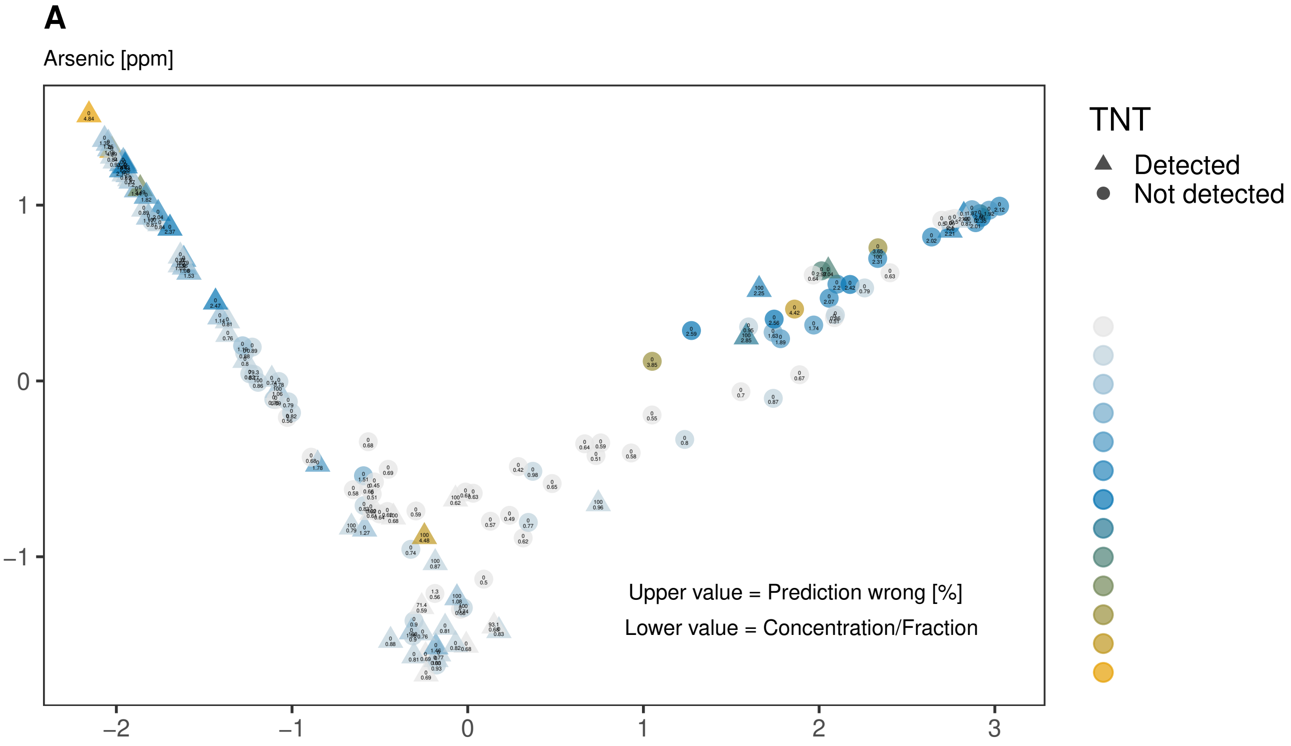
A
Arsenic [ppm]
TNT
Detected
Not detected
Upper value = Prediction wrong [%]
Lower value = Concentration/Fraction
1
0
−1
−2
−1
0
1
2
3

B

63 – 125 µm fraction [%]

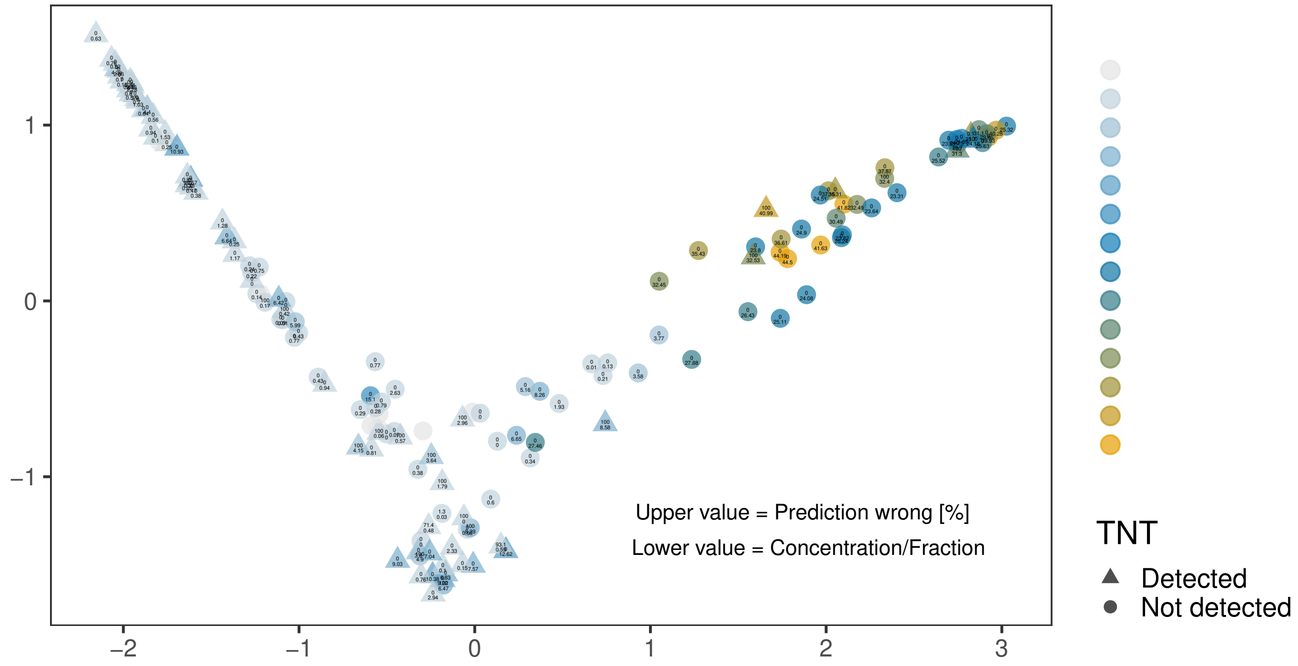

C

250 – 500 µm fraction [%]

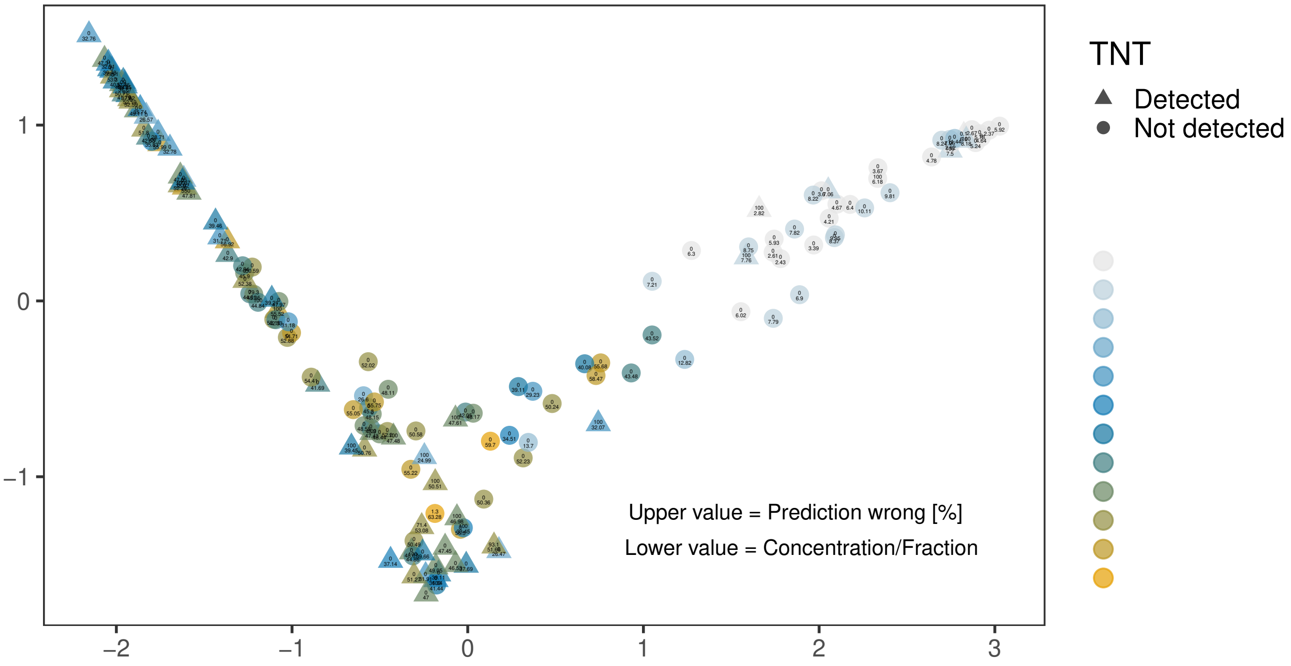

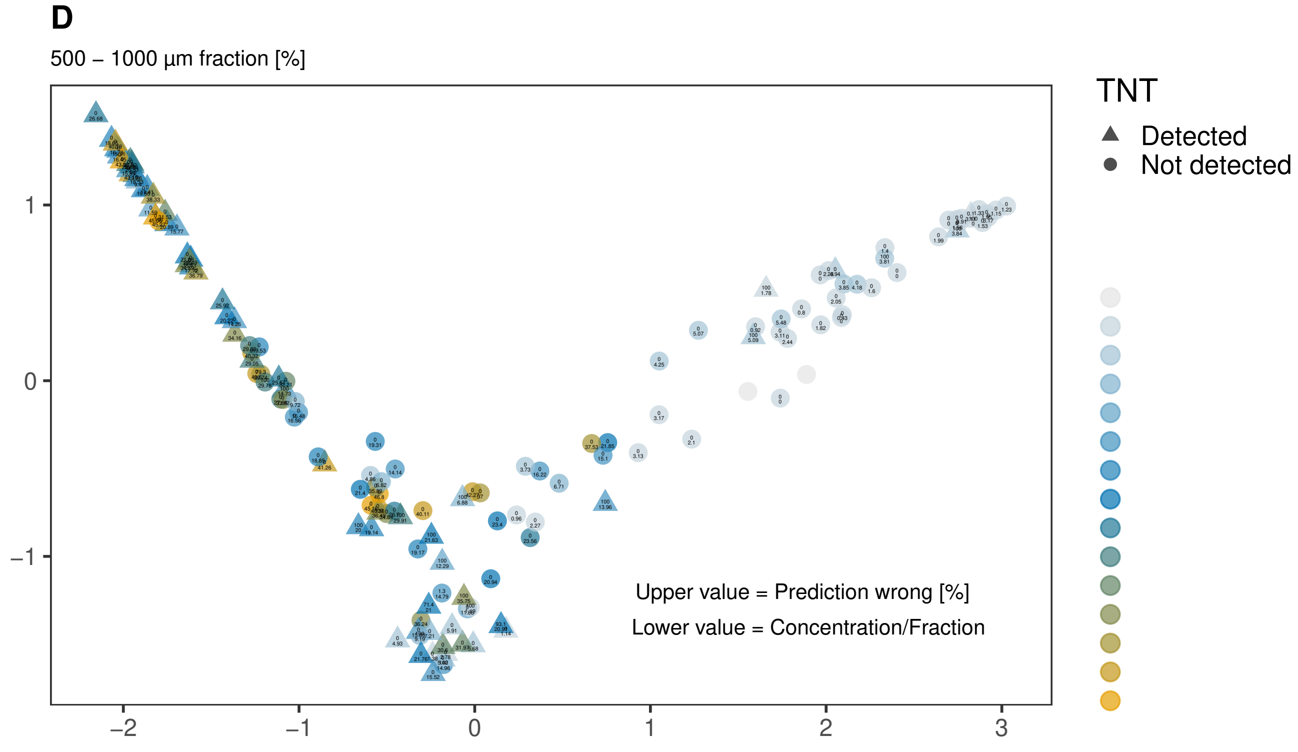
D
500 – 1000 µm fraction [%]
TNT
Detected
Not detected
Upper value = Prediction wrong [%]
Lower value = Concentration/Fraction
1
0
−1
−2
−1
0
1
2
3

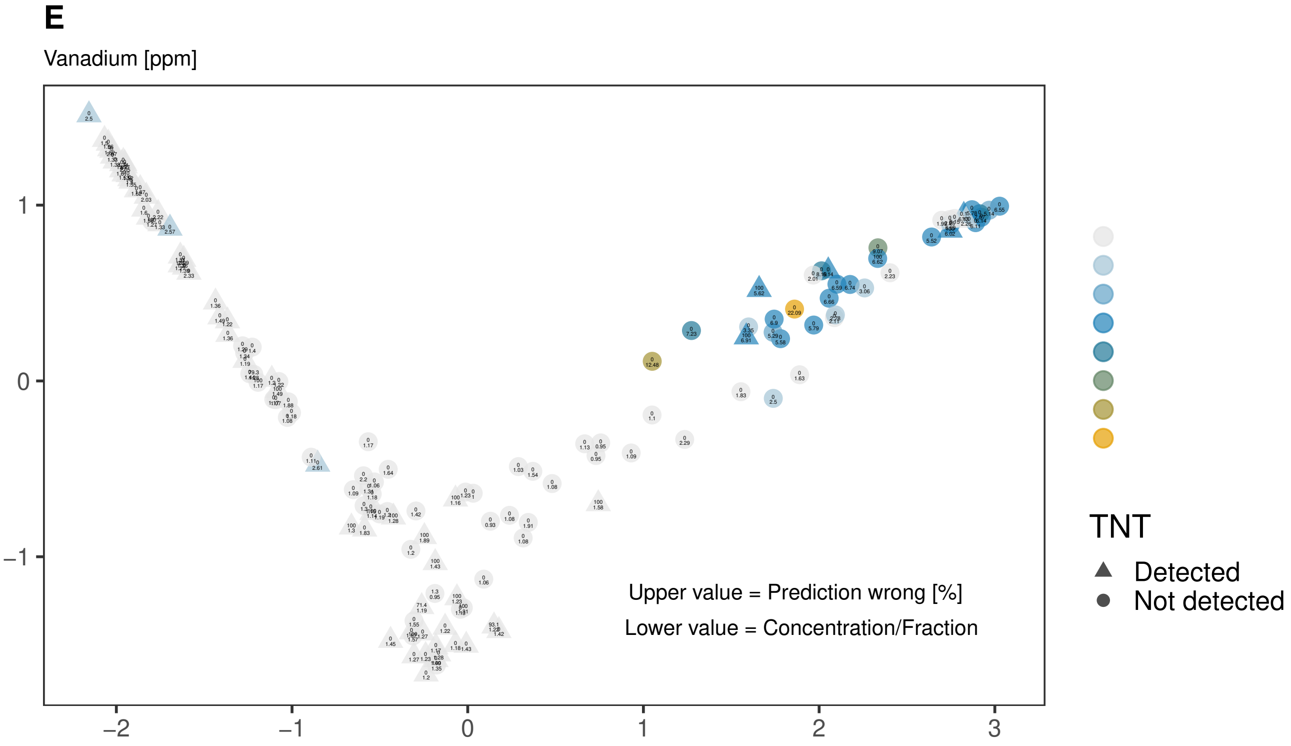
E
Vanadium [ppm]
Upper value = Prediction wrong [%]
Lower value = Concentration/Fraction
TNT
Detected
Not detected

F

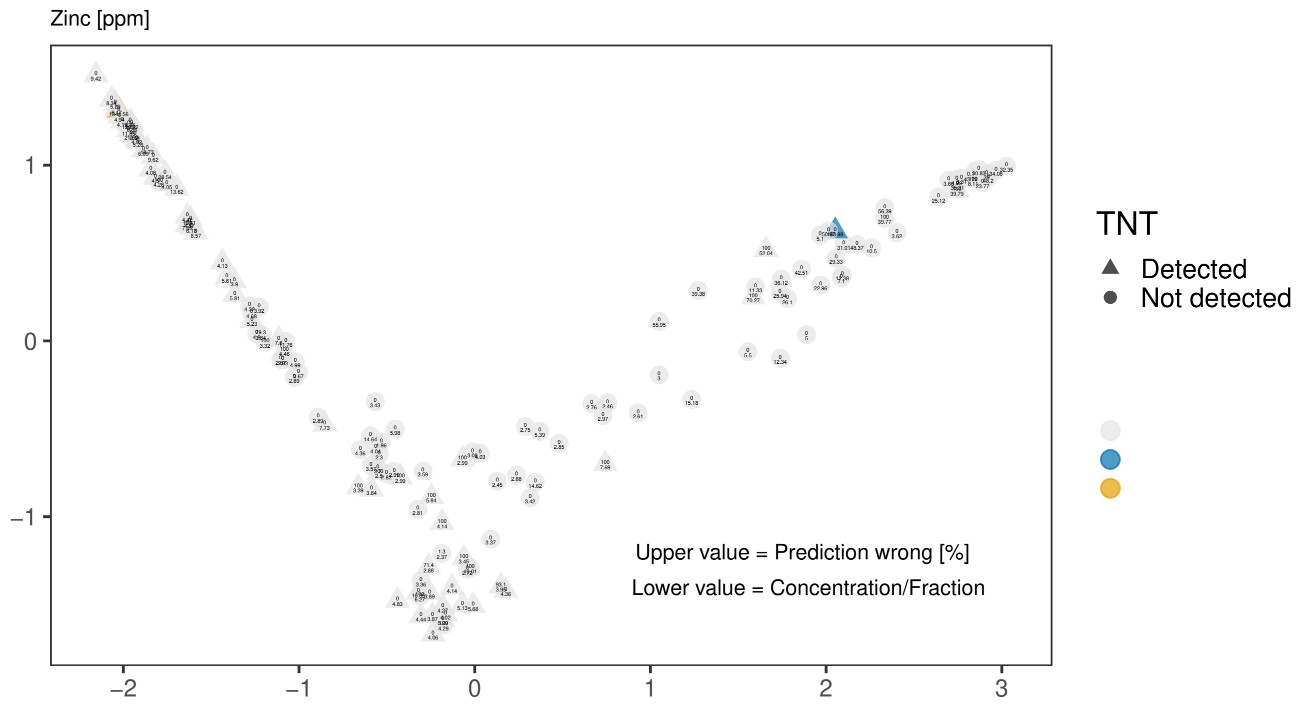

Zinc [ppm]
TNT
Detected
Not detected
Upper value = Prediction wrong [%]
Lower value = Concentration/Fraction

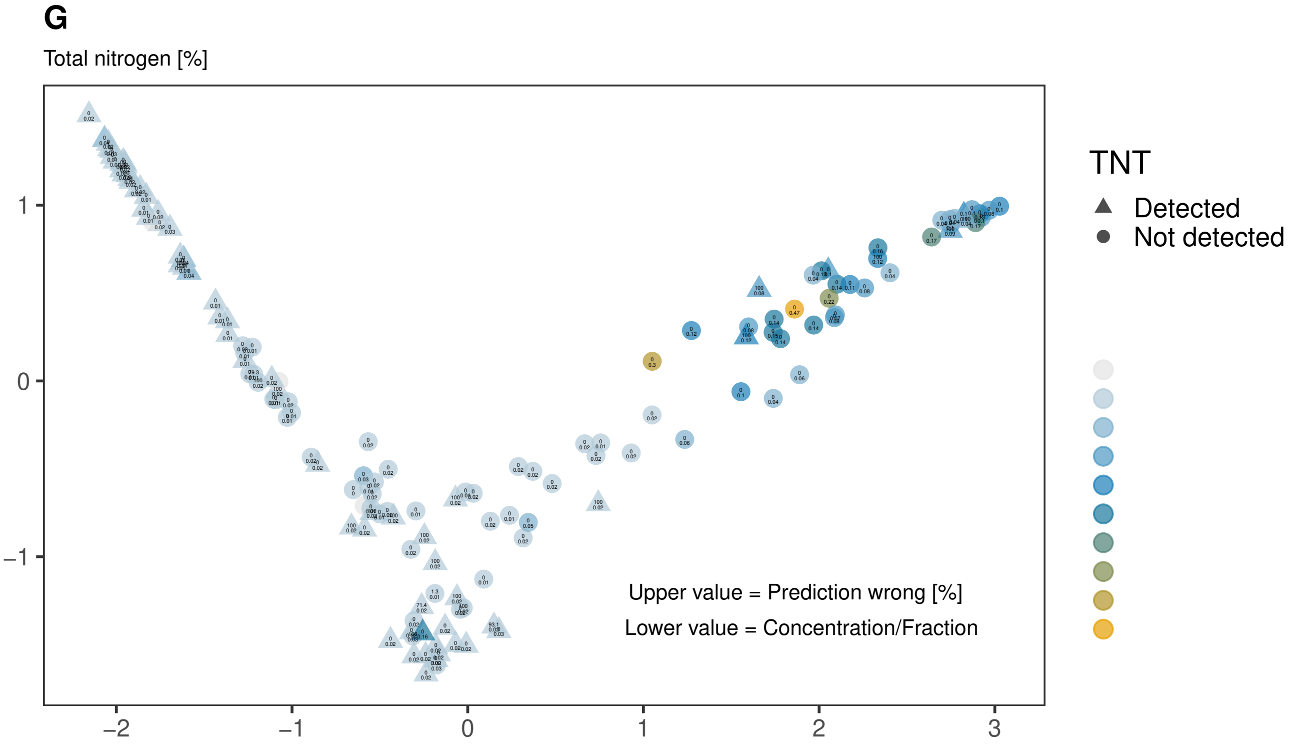
G
Total nitrogen [%]
TNT
Detected
Not detected
Upper value = Prediction wrong [%]
Lower value = Concentration/Fraction
−2
−1
0
1
2
3

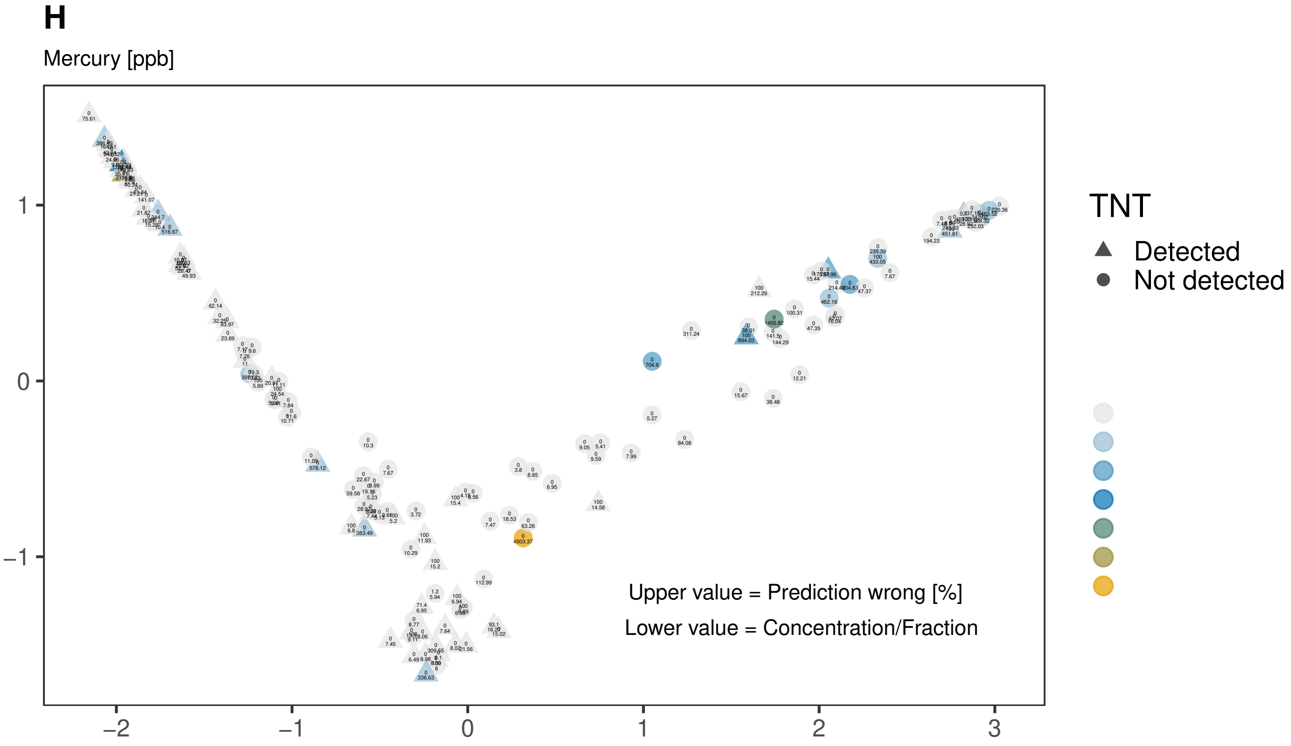
H
Mercury [ppb]
TNT
Detected
Not detected
Upper value = Prediction wrong [%]
Lower value = Concentration/Fraction
1
0
−1
−2
−1
0
1
2
3

I

Cobalt [ppm]

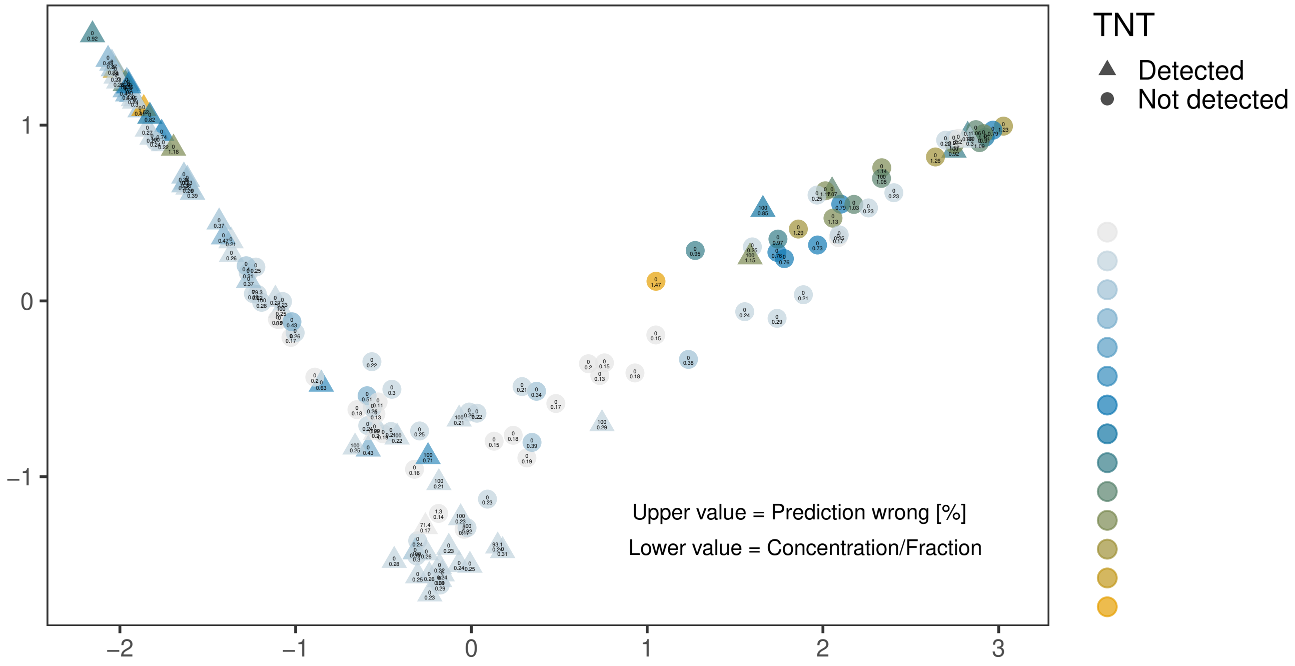
TNT
Detected
Not detected
Upper value = Prediction wrong [%]
Lower value = Concentration/Fraction
1
0
−1
−2
−1
0
1
2
3

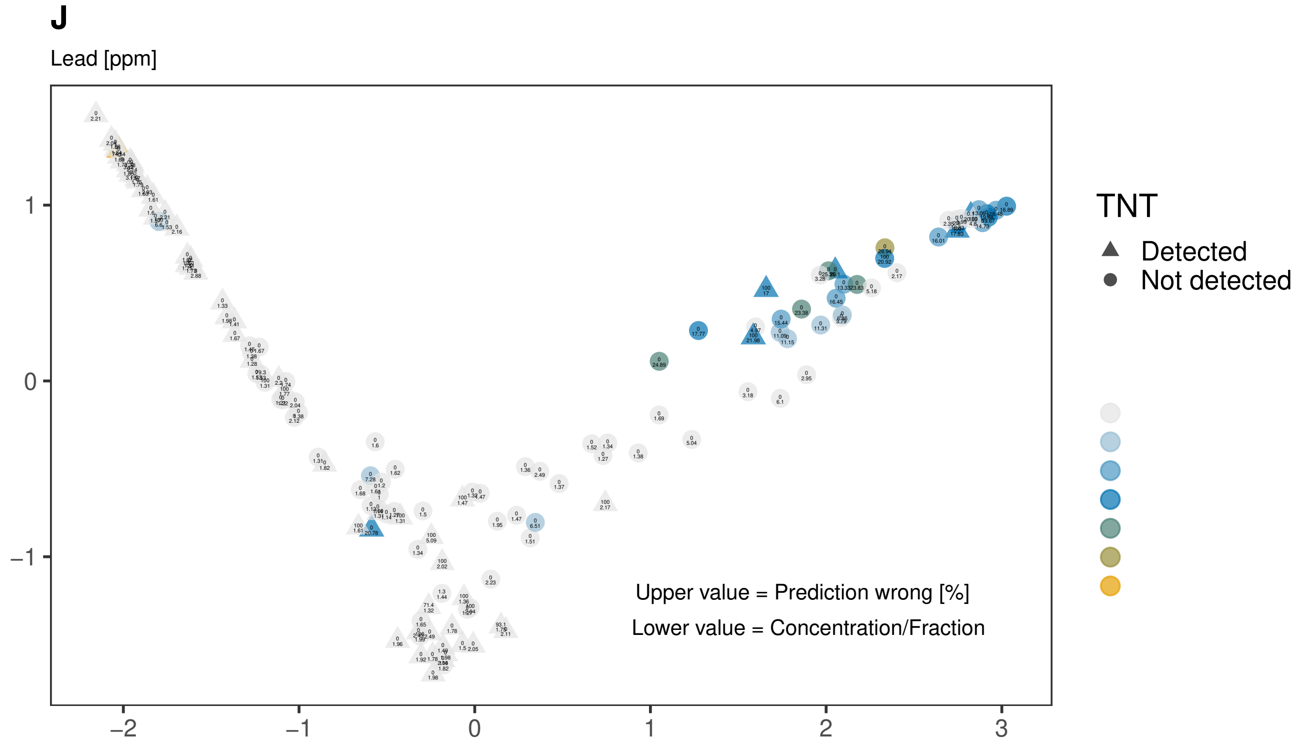
J
Lead [ppm]
TNT
Detected
Not detected
Upper value = Prediction wrong [%]
Lower value = Concentration/Fraction

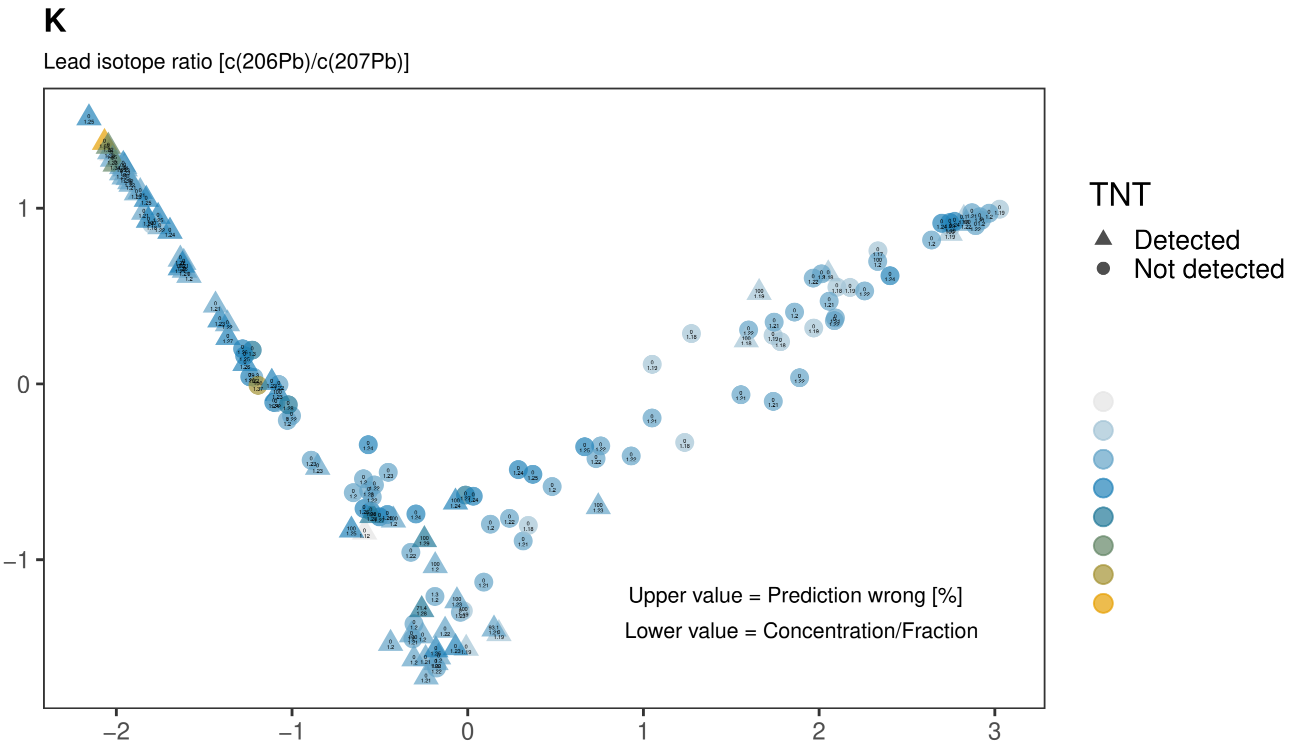
K
Lead isotope ratio [c(206Pb)/c(207Pb)]
TNT
Detected
Not detected
Upper value = Prediction wrong [%]
Lower value = Concentration/Fraction
−2
−1
0
1
2
3
1
0
−1
