# Supplementary material for: Machine Learning Predicts the Presence of 2,4,6-Trinitrotoluene in Sediments of a Baltic Sea Munitions Dumpsite Using Microbial Community Compositions
Source: Front Microbiol. 2021 Sep 29;12:626048. doi: 10.3389/fmicb.2021.626048 (PMC8513674; doi:10.3389/fmicb.2021.626048)
Supplement: Supplementary file 1 [file Data_Sheet_1.zip › Supplements_update_09_28/Supplementary_Figure_11_supervised_PCA_genus_area_noline.docx]

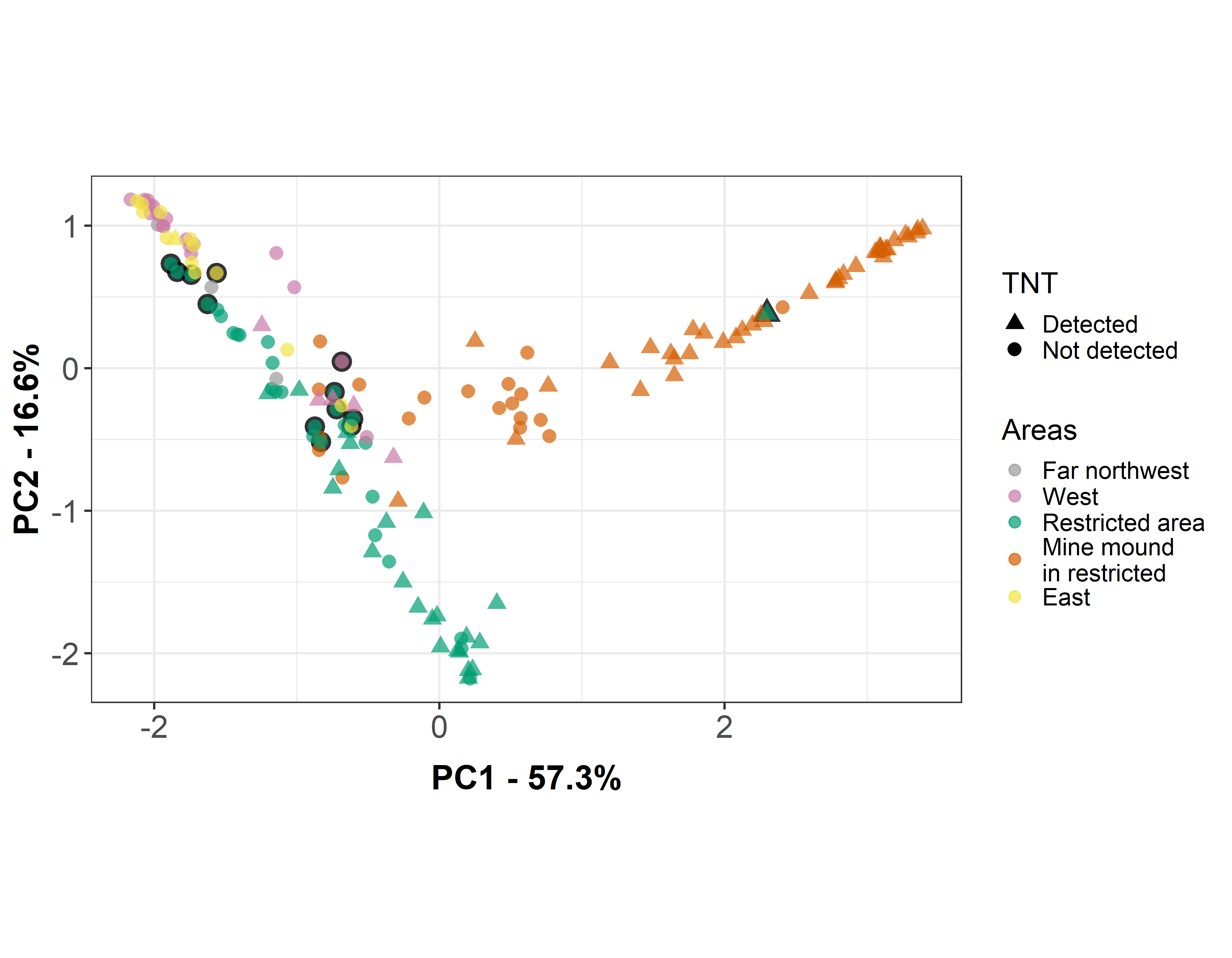


Supplementary Figure 11: PCA ordination based on the abundance of the most important 25 genera. Dissimilarity calculated using the proximity matrix of TNT-classifying supervised random forest. The microbial communities were colored by sample area and shaped indicating the presence of TNT. The East (yellow) and West (purple) samples with a black outline were not multicorer samples. The restricted area samples with a black outline were not part of a transect. Sediments containing TNT could be separated to the top right and to the center bottom, absent samples were located in the top left. The central area contained sediments with and without TNT.
