# Supplementary material for: Machine Learning Predicts the Presence of 2,4,6-Trinitrotoluene in Sediments of a Baltic Sea Munitions Dumpsite Using Microbial Community Compositions
Source: Front Microbiol. 2021 Sep 29;12:626048. doi: 10.3389/fmicb.2021.626048 (PMC8513674; doi:10.3389/fmicb.2021.626048)
Supplement: Supplementary file 1 [file Data_Sheet_1.zip › Supplements_update_09_28/Supplementary_Figure_12_mercury_arsenic_lead_pointsource_noline.docx]

Supplementary Figure 12: This document contains further information on the presence of certain heavy metals within all collected sediments (not limited to the 150 selected samples) and more specifically along depth profiles and in defined distances to mines.

**Results and** **discussion:**

In 167 sediments, Hg ranged from 3.7 to 4503.4 µg Hg∙kg^−1^ dry sediment, with a median of 20.5 µg and 15 samples exceeding 450 µg. The maximal concentration was found during a line transect, where the neighboring samples in 20 m distance contained 8 and 12 µg. Arsenic was detected from 0.4 to 4.8 ppm with a median of 0.8 and lead ranged from 1 to 75 ppm with a median of 2.

UXO have been proposed as point sources of heavy metals, especially mercury and lead. They were installed as highly toxic primary explosives mercury(II) fulminate, lead azide and lead styphnate. The mercury background in the Baltic Sea was estimated at 20 to 50 µg Hg∙kg^−1^ dry sediment (Leipe et al., 2013). They also mentioned 250 µg∙kg^−1^ as highest surface value in the northern Baltic sea and 450 µg∙kg^−1^ several cm deeper of. A similar trend was shown within the western multicorer cores, although concentrations at 10 cm depth reached up to 900 µg Hg∙kg^−1^.


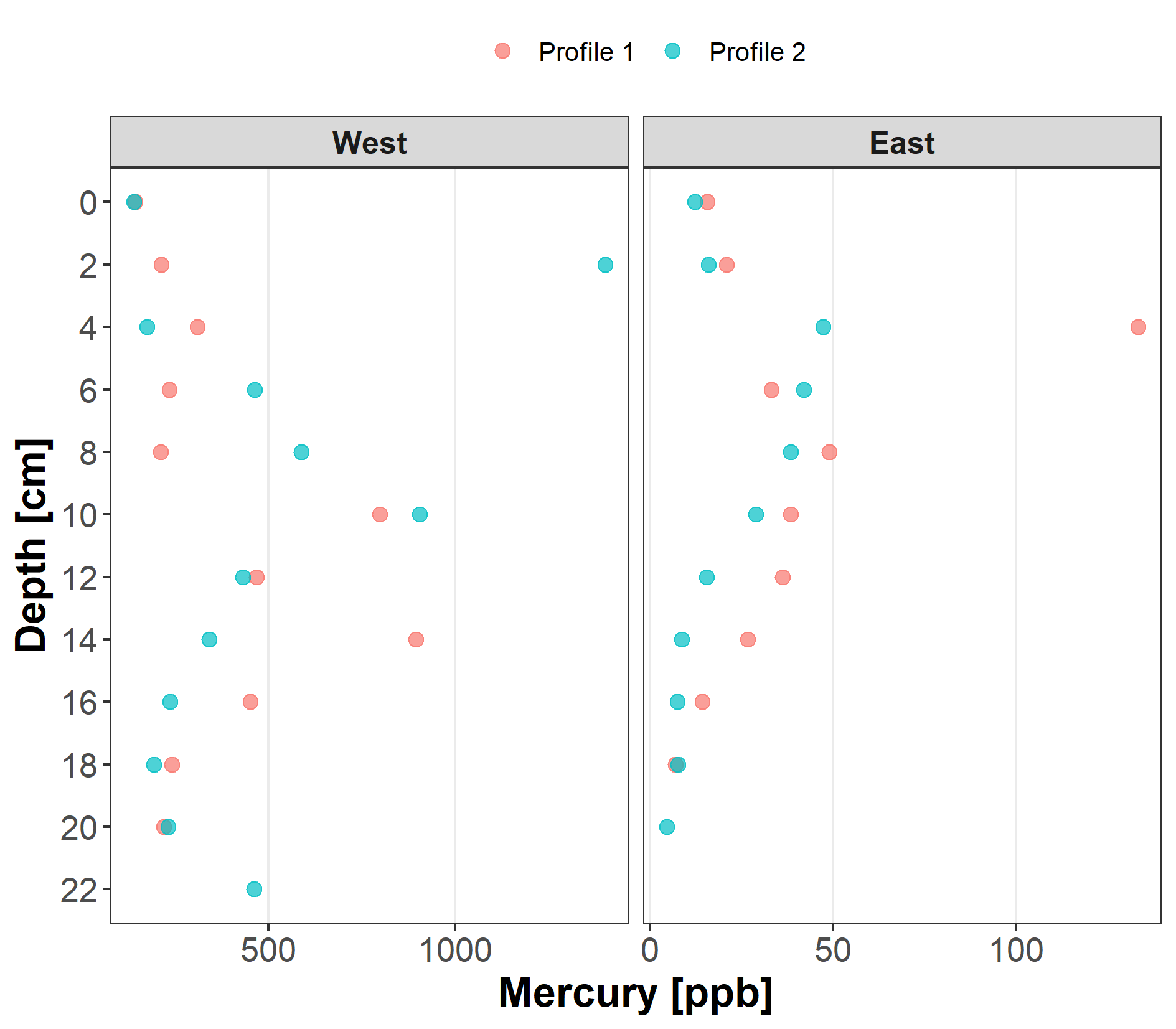


Mercury concentrations along the depth profiles taken with a multicorer east and west of the Kolberger Heide. Please not the different scales on the x axis.

Bełdowski et al. (2019) detected a maximal concentration of 322.2 µg Hg∙kg^−1^ mercury in 8 Kolberger Heide top layer sediments with high variance between sediments. In our study spottily occurring high values of up to 4503 µg Hg∙kg^−1^ were detected, too. Within 2 m of a mine the highest values were measured at 0.5 m distance (mean 329 µg Hg∙kg^−1^, median 75.6 µg Hg∙kg^−1^) distance. However, the other sediments within 2 m radius of the same mine did not show such elevated levels (Supplementary Material 2), potentially because the mines at Kolberger Heide are classified as discarded munition material. In comparison to unexploded ordnance, those were not fused and therefore should not contain mercury(II) fulminate.


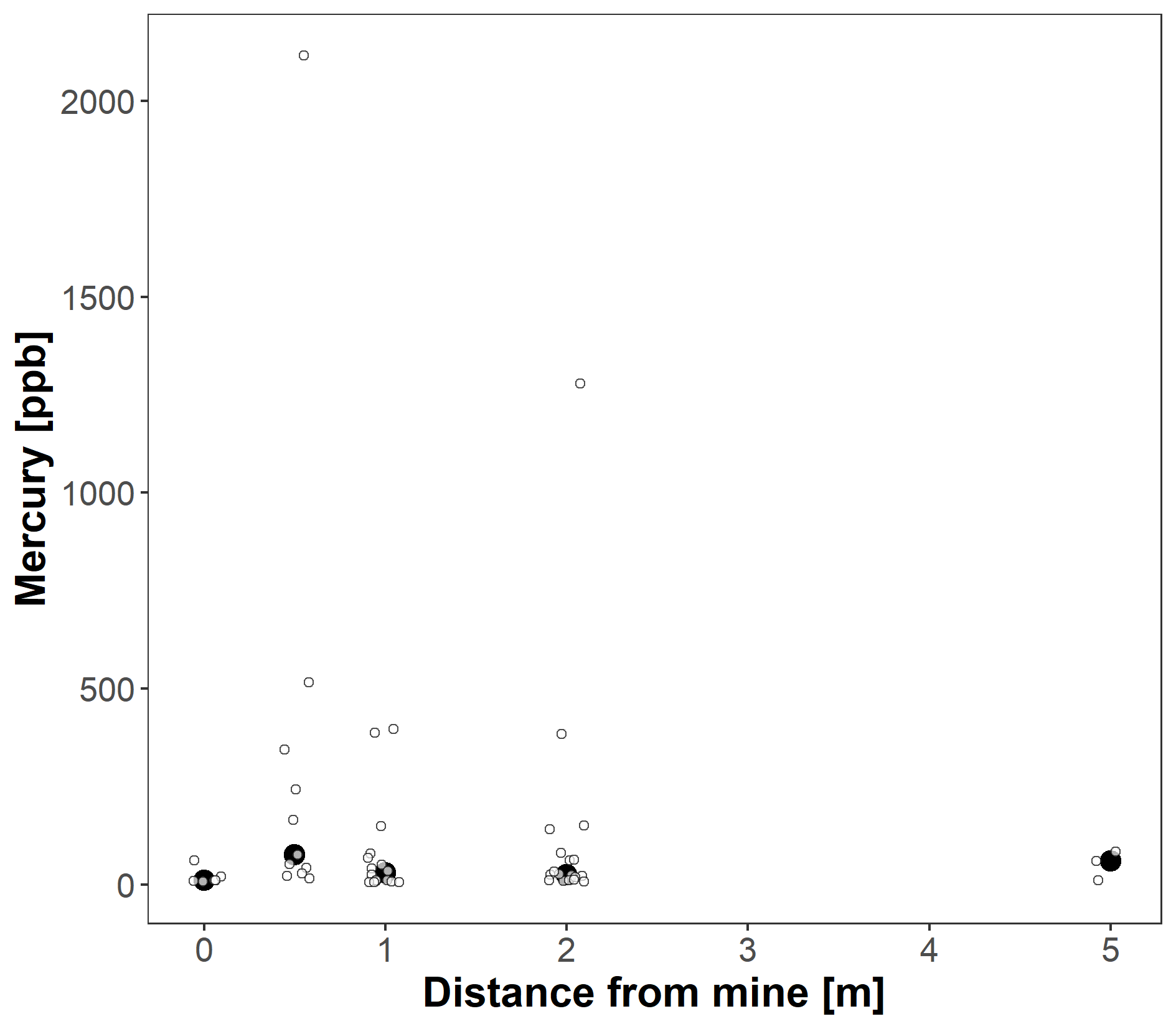


Mercury concentrations in sediments sampled in a maximal distance of 5 m around mines. The black filled dot is the median concentration. Samples from 0.5 to 2 m distance originated from a cardinal direction wise sampling around 3 distinct mines. Sediments of 0 and 5 m stem from a linear distance sampling. All sampling took place in the mine mound area.

It has yet to be determined why rare samples demonstrate such high concentrations. There was no significant correlation over all sediment samples for Hg with TNT (Supplementary Material 7), though both substances would likely be transported differently if originating from the same mine.

Lead concentrations fitted within expected Baltic Sea sediment background (Zaborska, 2014).

The important variable arsenic caught our attention, as it also is a compound of chemical warfare agents. However, its median concentration did not exceed e.g. the average southeastern Baltic Sea background of 3.4 ppm (Garnaga et al., 2006). and chemical warfare agents were to our knowledge not disposed of in the Kolberger Heide (Böttcher et al., 2011; Bełdowski et al., 2016).

**References:**

Bełdowski, J., Szubska, M., Emelyanov, E., Garnaga, G., Drzewińska, A., Bełdowska, M., et al. (2016). Arsenic concentrations in Baltic Sea sediments close to chemical munitions dumpsites. *Deep. Res. Part II Top. Stud. Oceanogr.* 128, 114–122. doi:10.1016/j.dsr2.2015.03.001.

Bełdowski, J., Szubska, M., Siedlewicz, G., Korejwo, E., Grabowski, M., Bełdowska, M., et al. (2019). Sea-dumped ammunition as a possible source of mercury to the Baltic Sea sediments. *Sci. Total Environ.* 674, 363–373. doi:10.1016/j.scitotenv.2019.04.058.

Böttcher, C., Knobloch, T., Rühl, N.-P., Sternheim, J., Wichert, U., and Wöhler, J. (2011). Munitionsbelastung der deutschen Meeresgewässer – Bestandsaufnahme und Empfehlungen. Available at: www.munition-im-meer.de.

Garnaga, G., Wyse, E., Azemard, S., Stankevičius, A., and de Mora, S. (2006). Arsenic in sediments from the southeastern Baltic Sea. *Environ. Pollut.* 144, 855–861. doi:10.1016/j.envpol.2006.02.013.

Leipe, T., Moros, M., Kotilainen, A., Vallius, H., Kabel, K., Endler, M., et al. (2013). Mercury in Baltic Sea sediments - Natural background and anthropogenic impact. *Geochemistry* 73, 249–259. doi:10.1016/j.chemer.2013.06.005.

Zaborska, A. (2014). Anthropogenic lead concentrations and sources in Baltic Sea sediments based on lead isotopic composition. *Mar. Pollut. Bull.* 85, 99–113. doi:10.1016/j.marpolbul.2014.06.013.
