# Supplementary material for: Machine Learning Predicts the Presence of 2,4,6-Trinitrotoluene in Sediments of a Baltic Sea Munitions Dumpsite Using Microbial Community Compositions
Source: Front Microbiol. 2021 Sep 29;12:626048. doi: 10.3389/fmicb.2021.626048 (PMC8513674; doi:10.3389/fmicb.2021.626048)
Supplement: Supplementary file 1 [file Data_Sheet_1.zip › Supplements_update_09_28/Supplementary_Figure_13_Classifications_RF_ANN_noline.docx]

Supplementary Figure 13: A comparison of achieved classification scores of Random Forest and Artificial neural nets. In case of community data, Random Forest used the relative abundances directly, whereas the values for ANN were z-score transformed (zero mean with unit variance). The ANN hyperparameters are described in the methods section. The ANN performed slightly poorer in almost all analyses, but were more demanding to train and hardly to interpret and offer a lot more options to choose from.


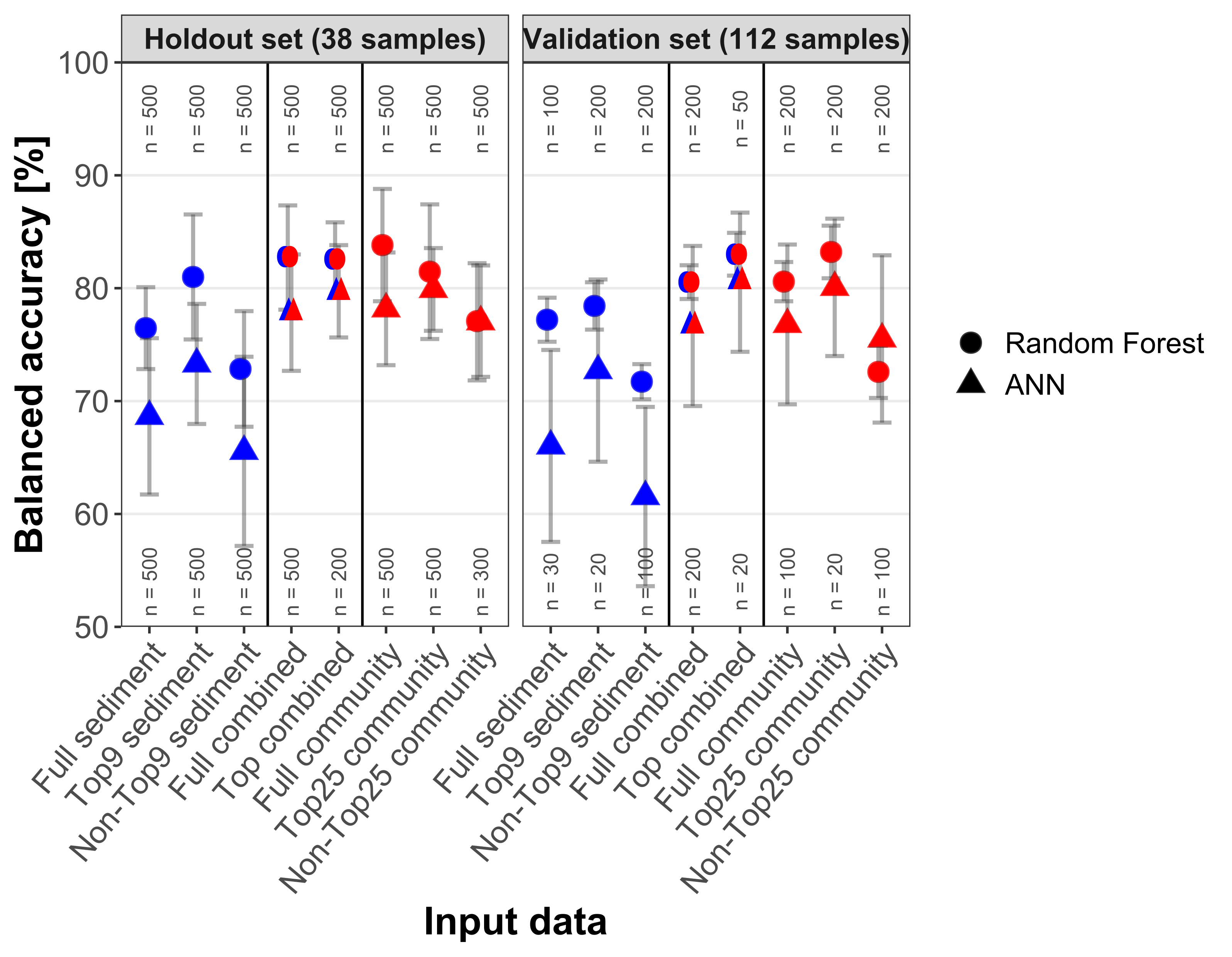


Correct TNT classification per input data in the validation and holdout test set for RF (dot) and ANN (triangle). Red indicates community data, blue symbolizes sediment data and red-blue combined variables. Of each data type, either all variables were utilized by the model (“Full”), or only the best variables best on variable importance (“Top”) or all variables except Top (“Non-Top”). Classification performance is displayed as mean and standard deviation of balanced accuracy, the classification results of the six different data sets were averaged. The RF validation values are out-of-bag estimates. n indicates the number of RF (top) and ANN (bottom) models calculated. Figure modified after Janßen (2020).

**References:**

Janßen, R. (2020). Machine learning classification of microbial community compositions to predict anthropogenic pollutants in the Baltic Sea. doi:https://doi.org/10.18453/rosdok_id00002897.
